# Lanostane–Meroterpene Conjugates with Unusual Aryl Ether Linkage and a Lanostane Dimer from Artificially Cultivated Fruiting Bodies of *Ganoderma* cf. *hochiminhense*

**DOI:** 10.1021/acsomega.5c06826

**Published:** 2025-10-13

**Authors:** Malipan Sappan, Panida Chinthanom, Kitlada Srichomthong, Tuksaporn Thummarukcharoen, Rattaket Choeyklin, Aphidech Sangdee, Masahiko Isaka

**Affiliations:** † National Center for Genetic Engineering and Biotechnology (BIOTEC), National Science and Technology Development Agency (NSTDA), 111 Thailand Science Park, Phahonyothin Road, Klong Luang, Pathumthani 12120, Thailand; ‡ National Biobank of Thailand, National Science and Technology Development Agency (NSTDA), 111 Thailand Science Park, Phahonyothin Road, Klong Luang, Pathumthani 12120, Thailand; § Department of Biology, Faculty of Science, 54783 Mahasarakham University, Khamriang, Kantarawichai, Maha Sarakham 44150, Thailand

## Abstract

Ten previously undescribed compounds, including eight lanostane–meroterpene conjugates, ganohochimins A–H (**1**–**8**), a lanostane dimer, ganohochimate A (**9**), and a meroterpenoid, fornicin F (**10**), were isolated from the cultivated mushroom *Ganoderma cf. hochiminhense*­(strain TBRC-BCC 22084). The 24*S*-configuration of ganohochimin A (**1**) was determined by cleavage of the aryl ether under acidic conditions. The structure of ganohochimate A (**9**) was confirmed by alkaline hydrolysis to cleave into two lanostane units. Ganohochimins exhibited cytotoxicity against NCI-H187 (small-cell lung cancer) cells (IC_50_ 7.7–33 μM) and noncancerous Vero cells (IC_50_ 6.3–30 μM).

## Graphical abstract

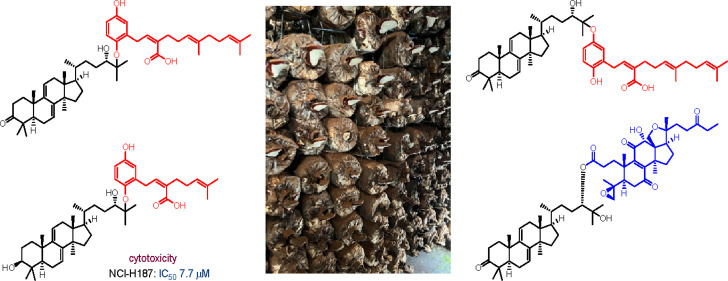

## Introduction

Polypores of the genus *Ganoderma* are popular medicinal mushrooms.^1^ *Ganoderma* species in the *Ganoderma lucidum* complex,^2^ such as *G. lucidum*, *G. sichuanense*, and *G. lingzhi*, are traditional Chinese medicines known as Lingzhi and have been widely used in supplement products.^3^ *Ganoderma* species are sources of lanostane-type triterpenoids,^1,4,5^ which have been considered as key ingredients related to the various health improving properties of the medicinal mushrooms. *Ganoderma* triterpenoids have been reported to exhibit antioxidation, anti-inflammatory, immune regulation, antihyperglycemic, antihyperlipidemic, antitumor, and antiviral effects.^1,4–6^ However, there are still many species in this mega genus that remain chemically unexplored or not well investigated. The quantities of natural mushroom specimens for such species are usually limited and not enough for authentic chemical investigations. Our current research is based on the artificial fruiting body cultivations of these minor *Ganoderma* species by applying the protocols for the factory productions of the medicinal mushroom Lingzhi (*G. lucidum*).

*Ganoderma hochiminhense* Karunarathna, Mortimer, & Luangharn was described in 2021.^7^ Recently, we reported isolation of highly modified lanostanes from natural mushroom specimens of*G. cf. hochiminhense*, that were collected from trunk of a dead oil palm (*Elaeis guineensis*), Krabi Province, Thailand.^8^ The results demonstrated that this species is a source of structurally intriguing lanostane-type triterpenoids. In continuation of this research, artificial fruiting body cultivation was conducted using another strain (TBRC-BCC 22084) of *G. cf. hochiminhense*, collected at a different location. Its chemical investigation led to the isolation of ten undescribed compounds, including eight lanostane–meroterpene conjugates, gamohochimins A–H (**1**–**8**), a lanostane dimer, ganohochimate A (**9**), and a meroterpenoid, fornicin F (**10**) (Figure 1). Twenty-five known compounds (**11**–**35**, Figure S1), including ganomycin B (**11**),^9^ ganodermanondiol (**12**),^10^ and lucidumol B (**13**),^11^ were also isolated.

**Figure 1:**
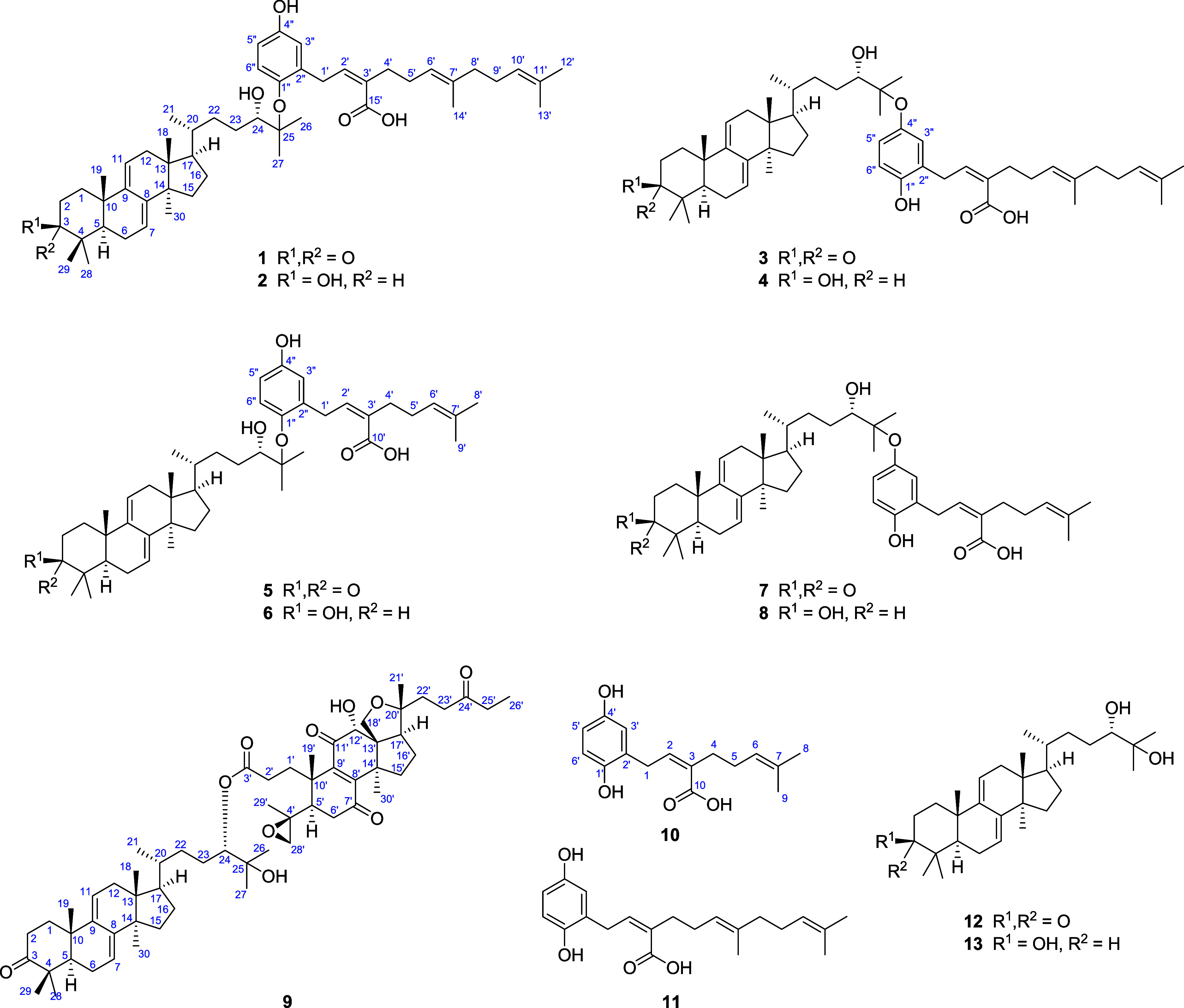
Structures of compounds **1**–**13**.

## Results and Discussion

The CH_2_Cl_2_ extract from fruiting bodies of *G. cf. hochiminhense*was fractionated by silica gel column chromatography, and the column fractions were further separated by preparative HPLC (ODS column) to furnish five new compounds, **1**, **2**, **7**, **8**, and **10**. Twenty-five known compounds, **11**–**35**, were also isolated from the same mushroom extract. Their chemical structures and corresponding references are shown in the Supporting Information (Figure S1). Considering the intriguing chemical structures of the new compounds, the fungus was cultivated again in a 4-fold larger scale. Nine new compounds, **1**–**9**, were isolated from the batch 2 cultivation material (Figure S2).

Ganohochimin A (**1**) was obtained as a colorless solid. The molecular formula was determined as C_51_H_74_O_6_ based on its HRMS (ESI-TOF) data. The ^1^H and ^13^C NMR, DEPT, and HSQC data of **1** suggested a lanostane–meroterpene conjugate structure (Table 1). The lanostane skeleton was deduced from interpretation of COSY and HMBC spectra (Figure 2). The lanostane unit was elucidated to be the same as the cometabolite, ganodermanondiol (**12**),^10^ whereas the meroterpene unit was identical to ganomycin B (**11**).^11^ The C-25 oxygenated carbon (δ_C_ 83.9) of the lanostane unit was downfield shifted when compared with that of the isolate **12** (δ_C_ 73.2, C-25). There were significant differences in the ^1^H and ^13^C chemical shifts data for the hydroquinone ring of the meroterpene unit when compared with those of **11** (Figure 3). In particular, the hydroquinone carbons, C-2″ and C-6″, and H_2_-1′ were downfield shifted in **1**. These data strongly suggested the aryl ether linkage at C-1″ of the meroterpene (Figure 3). Also, an ester linkage between 25-OH of ganodermanondiol and C-15′ carboxylic acid of ganomycin B was ruled out. NOESY correlations between H_3_-26/H-6″, H_3_-27/H-6″, H-24/H-6″ (weak), and H-24/H_2_-1′ (weak) further supported the aryl ether linkage between C-25 of the lanostane and C-1″ of the meroterpene. The location of the linkage at C-25 and the absolute configuration of the oxymehine C-24 were revealed by degradation of **1** under acidic conditions. Treatment of **1** in CDCl_3_ with *p*-TsOH·H_2_O afforded a mixture of two lanostanes **36** and **37** (Figure 4). These lanostanes should be produced via C-25 carbonium ion intermediate with elimination of H-26 (to form an exomethylene) and H-24 (to form an enol), respectively. Compound **36** was previously isolated in our laboratory from fruiting bodies of *Ganoderma* sp.,^12^ and its 24*S*-configulation was determined using the modified Mosher method. Consequently, the 24*S*-configuration of **1** was established.

**Table 1:** NMR Spectroscopic Data for Ganohochimins A–D (**1**–**4**) in CDCl_3_

	ganohochimin A (**1**)		ganohochimin B (**2**)		ganohochimin C (**3**)		ganohochimin D (**4**)	
no.	δ_C_, type	δ_H_, mult. (*J* in Hz)	δ_C_, type	δ_H_, mult. (*J* in Hz)	δ_C_, type	δ_H_, mult. (*J* in Hz)	δ_C_, type	δ_H_, mult. (*J* in Hz)
1	36.6, CH_2_	α 1.75, m; β2.28, m	35.7, CH_2_	α 1.44, m; β1.99, m	36.6, CH_2_	α 1.76, m; β2.28, m	35.7, CH_2_	α 1.44, m; β1.99, m
2	34.8, CH_2_	α 2.34, m; β2.78, m	27.8, CH_2_	α 1.71, m; β 1.65, m	34.8, CH_2_	α 2.35, m; β2.78, m	27.9, CH_2_	α 1.72, m; β 1.65, m
3	216.9, C		79.0, CH	3.25, dd (11.4, 4.4)	217.1, C		79.0, CH	3.26, dd (11.5, 4.4)
4	47.5, C		38.7, C		47.5, C		38.7, C	
5	50.7, CH	1.53, dd (11.9, 3.7)	49.2, CH	1.09, dd (11.1, 4.6)	50.7, C	1.54, dd (11.7, 3.7)	49.1, CH	1.09, m
6	23.7, CH_2_	α 2.05, m; β 2.20, m	23.0, CH_2_	α 2.08, m; β 2.07, m	23.7, CH_2_	α 2.06, m; β 2.20, m	23.0, CH_2_	α 2.09, m; β 2.08, m
7	119.9, CH	5.50, d (6.5)	120.2, CH	5.47, d (4.8)	119.9, CH	5.51, d (6.6)	120.2, CH	5.48, d (5.6)
8	142.9, C		142.7 C		142.9, C		142.6, C	
9	144.5, C		146.0, C		144.5, C		145.9, C	
10	37.2, C		3740, C		37.2, C		37.3, C	
11	117.3, CH	5.38, d (6.0)	116.3, CH	5.31, d (6.1)	117.3, CH	5.39, d (5.9)	116.3, CH	5.31, d (6.2)
12	37.9, CH_2_	α 2.22, m; β 2.10, m	37.9, CH_2_	α 2.20, br d (17.6); β 2.08, m	37.9, CH_2_	α 2.23, m; β 2.12, m	37.8, CH_2_	α 2.21, br d (17.6); β 2.10, m
13	43.8, C		43.8, C		43.8, C		43.7, C	
14	50.3, C		50.3, C		50.3, C		50.3, C	
15	31.5, CH_2_	α 1.38, m; β 1.61, m	31.5, CH_2_	α 1.38, m; β 1.60, m	31.5, CH_2_	α 1.41, m; β 1.64, m	31.5, CH_2_	α 1.40, m; β 1.61, m
16	27.8, CH_2_	1.97, m; 1.37, m	27.8, CH_2_	1.97, m; 1.38, m	27.9, CH_2_	2.04, m; 1.41, m	27.7, CH_2_	2.02, m; 1.39, m
17	51.0, CH	1.60, m	51.1, CH	1.58, m	51.1, CH	1.60, m	51.0, CH	1.60, m
18	15.7, CH_3_	0.58, s	15.7, CH_3_	0.56, s	15.7, CH_3_	0.60, s	15.7, CH_3_	0.57, s
19	22.0, CH_3_	1.19, s	22.7, CH_3_	0.98, s	22.0, CH_3_	1.20, s	22.7, CH_3_	0.98, s
20	36.6, CH	1.42, m	36.6, CH	1.43, m	36.6, CH	1.43, m	36.6, CH	1.44, m
21	18.6, CH_3_	0.91, d (6.5)	18.6, CH_3_	0.91, d (6.4)	18.6, CH_3_	0.93, d (6.4)	18.6, CH_3_	0.92, d (6.5)
22	33.6, CH_2_	1.89, m; 1.06, m	33.6, CH_2_	1.89, m; 1.05, m	33.6, CH_2_	1.89, m; 1.03, m	33.6, CH_2_	1.91, m; 1.04, m
23	28.5, CH_2_	1.62, m; 1.26, m	28.5, CH_2_	1.62, m; 1.38, m	28.4, CH_2_	1.63, m; 1.27, m	28.4. CH_2_	1.63, m; 1.27, m
24	79.5, CH	3.61, dd (10.1, 1.5)	79.4, CH	3.60, dd (10.1, 1.6)	78.7, CH	3.54, dd (10.0, 1.2)	78.6, CH	3.53, dd (10.1, 1.8)
25	83.9, C		84.0, C		83.0, C			
26	22.6, CH_3_	1.15, s	22.7, CH_3_	1.15, s	23.0, CH_3_	1.18, s	23.0, CH_3_	1.17, s
27	21.0, CH_3_	1.27, s	21.0, CH_3_	1.26, s	20.4, CH_3_	1.18, s	20.4, CH_3_	1.17, s
28	25.4, CH_3_	1.09, s	28.1, CH_3_	1.01, s	25.4, CH_3_	1.09, s	28.1, CH_3_	1.01, s
29	22.4, CH_3_	1.13, s	15.8, CH_3_	0.88, s	22.4, CH_3_	1.13, s	15.8, CH_3_	0.88, s
30	25.4, CH_3_	0.87, s	25.6, CH_3_	0.87, s	25.4, CH_3_	0.88, s	25.6, CH_3_	0.88, s
1′	31.0, CH_2_	3.82 (2H), d (7.5)	30.9, CH_2_	3.81 (2H), d (7.4)	32.0, CH_2_	3.65 (2H), d (8.7)	32.0, CH_2_	3.64 (2H), d (8.7)
2′	142.5, CH	6.09, t (7.5)	142.3, CH	6.06, t (7.4)	141.0, CH	5.97, t (8.7)	140.9, CH	5.97, t (8.7)
3′	131.3,^a^ C		131.3, C		131.1, C		131.0, C	
4′	34.6, CH_2_	2.31 (2H), m	34.6, CH_2_	2.31 (2H), m	34.3, CH_2_	2.30 (2H), m	34.3, CH_2_	2.31 (2H), m
5′	27.6, CH_2_	2.16 (2H), m	27.6, CH_2_	2.16 (2H), m	27.4, CH_2_	2.14 (2H), m	27.3, CH_2_	2.14 (2H), m
6′	123.1, CH	5.10, m	123.1, CH	5.10, m	122.6, CH	5.04, m	122.5, CH	5.04, m
7′	136.1, C		136.1, C		136.5, C		136.5, C	
8′	39.7, CH_2_	1.95 (2H), m	39.7, CH_2_	1.95 (2H), m	39.6, CH_2_	1.92 (2H), m	39.6, CH_2_	1.91 (2H), m
9′	26.7, CH_2_	2.03 (2H), m	26.7, CH_2_	2.03 (2H), m	26.6, CH_2_	2.00 (2H), m	26.6, CH_2_	2.00 (2H), m
10′	124.3, CH	5.08, m	124.3, CH	5.08, m	124.3, CH	5.05, m	124.3, CH	5.05, m
11′	131.4,^a^ C		131.4, C		131.3, C		131.3, C	
12′	25.7, CH_3_	1.67, s	25.7, CH_3_	1.67, s	25.7, CH_3_	1.66, s	25.7, CH_3_	1.67, s
13′	17.7, CH_3_	1.59, s	17.7, CH_3_	1.59, s	17.7, CH_3_	1.58, s	17.7, CH_3_	1.58, s
14′	16.1, CH_3_	1.57, s	16.1, CH_3_	1.57, s	16.0, CH_3_	1.54, s	16.0, CH_3_	1.54, s
15′	171.7, C		171.1, C		173.0, C		172.6, C	
1″	146.1, C		146.2, C		151.9, C		152.0, C	
2″	135.1, C		135.2, C		123.3, C		123.2, C	
3″	116.8, CH	6.72, d (3.1)	116.8, CH	6.70, d (3.1)	126.3, CH	6.74, s	126.3, CH	6.74, d (1.6)
4″	151.7, C		151.6, C		146.8, C		146.7, C	
5″	113.6, CH	6.61, dd (8.7, 3.1)	113.6, CH	6.61, dd (8.7, 3.1)	124.1, CH	6.76,^b^ s	124.1, CH	6.76,^c^ m
6″	123.8, CH	6.88, d (8.7)	123.9, CH	6.88, d (8.7)	116.4, CH	6.76,^b^ s	116.4, CH	6.76,^c^ m

a The assignment of carbons may be interchanged.

b The aromatic proton signals were overlapped.

c The aromatic proton signals were overlapped.

**Figure 2:**
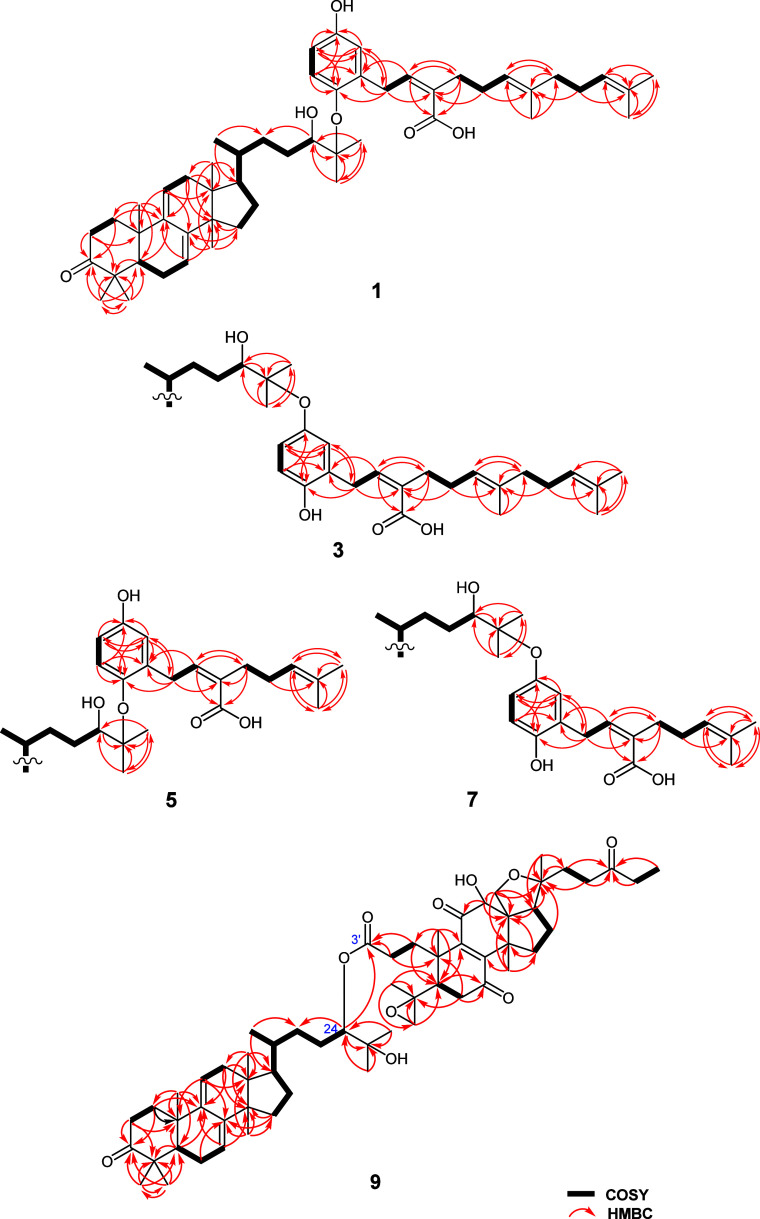
COSY and HMBC correlations for **1**, **3**, **5**, **7**, and **9**.

**Figure 3:**
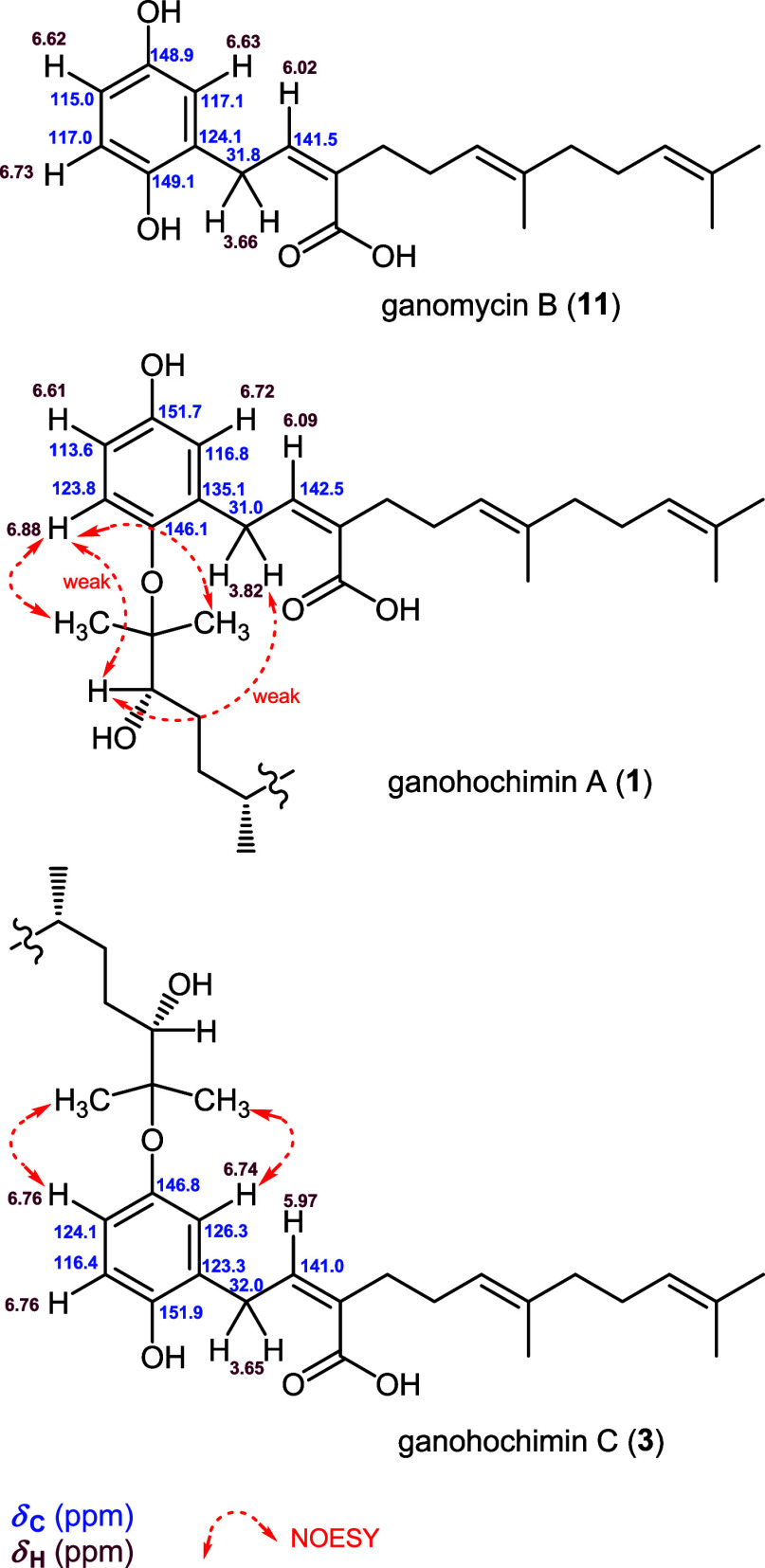
Comparison of the partial ^1^H and ^13^C NMR chemical shifts (CDCl_3_) for **1**, **3**, and ganomycin B (**11**) and key NOESY correlations.

**Figure 4:**
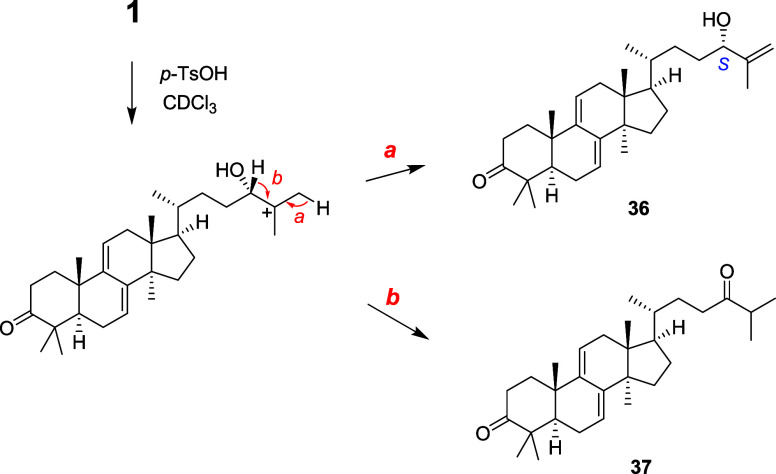
Acidic degradation of ganohochimin A (**1**).

Ganohochimin B (**2**) was given the molecular formula of C_51_H_76_O_6_ from HRMS (ESI-TOF). The NMR data (Table S1 and Figure S3) were similar to those of **1**. The only structural difference was the C-3 oxymethine (δ_C_ 79.0; δ_H_ 3.25, dd, *J* = 11.4, 4.4 Hz) instead of the C-3 ketone of **1**. The axial (α) orientation of the secondary alcohol proton H-3 was indicated based on its vicinal coupling constant values and NOESY correlations between H-3/H-5, H-3/H_α_-1, and H-3/H_3_-28 (Figure S4). Thus, the lanostane unit of **2** was identified to be the same as the cometabolite lucidumol B (**13**).

The elemental formula of ganohochimin C (**3**) was determined to be C_51_H_74_O_6_ from the HRESIMS data. Analysis of the 2D NMR spectroscopic data (Figure S3) led to the same lanostane and meroterpene units as **1**. There were differences in the chemical shifts of protons and carbons for the hydroquinone ring of the meroterpene with those of **1** and ganomycin B (**11**) (Figure 3). The hydroquinone carbons, C-3″ and C-5″, were downfield shifted in **3**, which suggested the aryl ether linkage at C-4″ of the meroterpene. NOESY cross-peaks of the singlet methyl signals of H_3_-26 and H_3_-27, overlapped at δ_H_ 1.26, with both H-3″ (δ_H_ 6.74) and H-5″ (δ_H_ 6.76), further supported the aryl ether linkage between C-25 of the lanostane and C-4″ of the meroterpene.

The elemental formula of ganohochimin D (**4**) was determined as C_51_H_76_O_6_ by HRESIMS. Its lanostane unit was elucidated to be the same as lucidumol B (**13**), whereas the NMR data for the meroterpene unit were close to those of **3**. Therefore, ganohochimin D (**4**) was identified to be the 3β-hydroxy derivative of ganohochimin C (**3**).

The elemental formula of ganohochimin E (**5**) was elucidated to be C_46_H_66_O_6_ based on the HRESIMS data. The NMR spectroscopic data (Table 2) revealed the same lanostane unit as ganohochimin A (**1**), whereas the meroterpene unit was smaller (5 less carbons) than that of ganomycin B (**11**). The meroterpene unit of **5**, a geranyl-hydroquinone, was deduced from the COSY and HMBC spectroscopic data (Figure 2). The 2′*Z*-configuration was determined based on the NOESY correlation between H-2′/H_2_-4′ (Figure S4). In the present study, the undescribed corresponding meroterpene, fornicin F (**10**), was also isolated (Figures S3 and S4). The aryl ether linkage between C-25 and C-1″ of **5** was indicated by the similarity of the NMR data for the hydroquinone ring with those of **1**, and the NOESY correlations between H_3_-26/H-6″ and H_3_-27/H-6″.

**Table 2:** NMR Spectroscopic Data for Ganohochimins E–H (**5**–**8**) in CDCl_3_

	ganohochimin E (**5**)		ganohochimin F (**6**)		ganohochimin G (**7**)		ganohochimin H (**8**)	
no.	δ_C_, type	δ_H_, mult. (*J* in Hz)	δ_C_, type	δ_H_, mult. (*J* in Hz)	δ_C_, type	δ_H_, mult. (*J* in Hz)	δ_C_, type	δ_H_, mult. (*J* in Hz)
1	36.6, CH_2_	α 1.75, m; β2.28, m	35.7, CH_2_	α 1.44, m; β1.99, m	36.6, CH_2_	α 1.76, m; β2.28, m	35.7, CH_2_	α 1.44, m; β2.00, m
2	34.8, CH_2_	α 2.34, m; β2.78, m	27.8, CH_2_	α 1.72, m; β1.65, m	34.8, CH_2_	α 2.35, m; β2.78, m	27.8,^a^ CH_2_	α 1.72, m; β1.65, m
3	216.9, C		79.0, CH	3.26, dd (11.4, 4.4)	216.9, C		79.0, CH	3.26, dd (11.5, 4.4)
4	47.5, C		38.7, C		47.5, C		38.7, C	
5	50.7, CH	1.53, dd (11.8, 3.7)	49.1, CH	1.09, dd (1110, 4.6)	50.7, CH	1.54, m	49.1, CH	1.09, dd (11.0, 4.7)
6	23.7, CH_2_	α 2.05, m; β2.20, m	23.0, CH_2_	α 2.08, m; β2.07, m	23.7, CH_2_	α 2.06, m; β2.21, m	23.0, CH_2_	α 2.10, m; β2.09, m
7	119.9, CH	5.50, d (6.5)	120.2, CH	5.47, d (5.7)	119.9, CH	5.51, d (6.7)	120.2, CH	5.48, d (5.6)
8	142.9, C		142.6, C		142.9, C		142.7, C	
9	144.5, C		145.9, C		144.5, C		146.0, C	
10	37.2, C		37.3, C		37.2, C		37.9, C	
11	117.3, CH	5.38, d (5.9)	116.2, CH	5.31, d (6.9)	117.3, CH	5.39, br d (6.3)	116.3, CH	5.32, br d (6.1)
12	37.9, CH_2_	α 2.22, m; β2.12, m	37.8, CH_2_	α 2.20, br d (17.2); β 2.08, m	37.9, CH_2_	α 2.23, m; β2.12, m	37.9, CH_2_	α 2.22, br d (17.6); β 2.10, m
13	43.8, C		43.7, C		43.8, C		43.8, C	
14	50.3, C		50.3, C		50.3, C		50.3, C	
15	31.5, CH_2_	α 1.38, m; β1.61, m	31.5, CH_2_	α 1.38, m; β1.59, m	31.5, CH_2_	α 1.42, m; β1.64, m	31.5, CH_2_	α 1.39, m; β1.64, m
16	27.8, CH_2_	1.98, m; 1.37, m	27.8, CH_2_	1.98, m; 1.37, m	27.9, CH_2_	2.04, m; 1.42, m	27.9,^a^ CH_2_	2.03, m; 1.40, m
17	51.0, CH	1.58, m	51.0, CH	1.58, m	51.1, CH	1.61, m	51.1, CH	1.60, m
18	15.7, CH_3_	0.58, s	15.7, CH_3_	0.56, s	15.7, CH_3_	0.60, s	15.7, CH_3_	0.58, s
19	22.0, CH_3_	1.19, s	22.6, CH_3_	0.98, s	22.0, CH_3_	1.20, s	22.7, CH_3_	0.98, s
20	36.5, CH	1.42, m	36.5, CH	1.41, m	36.6, CH	1.44, m	36.6, CH	1.43, m
21	18.6, CH_3_	0.90, d (6.4)	18.6, CH_3_	0.91, d (6.4)	18.7, CH_3_	0.93, d (6.5)	18.7, CH_3_	0.92, d (6.5)
22	33.6, CH_2_	1.89, m; 1.04, m	33.6, CH_2_	1.89, m; 1.04, m	33.6, CH_2_	1.90, m; 1.06, m	33.6, CH_2_	1.89, m; 1.05, m
23	28.5, CH_2_	1.62, m; 1.26, m	28.5, CH_2_	1.62, m’ 1.26, m	28.5, CH_2_	1.63, m; 1.27, m	28.5, CH	1.61, m; 1.26, m
24	79.5, CH	3.62, dd (10.1, 1.3)	79.4, CH	3.61, dd (9.9, 0.8)	78.7, CH	3.53, dd (10.1, 1.8)	78.7, CH	3.53, dd (10.1, 1.8)
25	83.9, C		84.0, C		83.0, C		83.0, C	
26	22.7, CH_3_	1.15, s	22.7, CH_3_	1.15, s	23.1, CH_3_	1.18, s	23.1, CH_3_	1.18, s
27	21.0, CH_3_	1.27, s	20.9, CH_3_	1.27, s	20.4, CH_3_	1.18, s	20.5, CH_3_	1.18, s
28	25.4, CH_3_	1.08, s	28.1, CH_3_	1.01, s	25.4, CH_3_	1.09, s	28.1, CH_3_	1.01, s
29	22.4, CH_3_	1.13, s	15.8, CH_3_	0.88, s	22.5, CH_3_	1.13, s	15.8, CH_3_	0.88, s
30	25.4, CH_3_	0.86, s	25.6, CH_3_	0.87, s	25.5, CH_3_	0.88, s	25.6, CH_3_	0.88, s
1′	30.9, CH_2_	3.81 (2H), d (7.4)	30.9, CH_2_	3.81 (2H), d (7.4)	31.9, CH_2_	3.64 (2H), d (8.7)	31.9, CH_2_	3.63 (2H), d (8.7)
2′	142.4, CH	6.07, t (7.4)	142.3, CH	6.06, t (7.4)	140.9, CH	5.95, t (8.7)	140.6, CH	5.93. t (8,7)
3′	131.3, C		131.2, C		131.0, C		131.2, C	
4′	34.6, CH_2_	2.29 (2H), m	34.6, CH_2_	2.30 (2H), m	34.4, CH_2_	2.31–2.30 (2H), m	34.4, CH_2_	2.30 (2H), br t (7.4)
5′	27.7, CH_2_	2.15 (2H), m	27.6, CH_2_	2.15 (2H), m	27.4, CH_2_	2.14–2.11 (2H), m	27.4, CH_2_	2.12 (2H), m
6′	123.3, CH	5.07, m	123.2, CH	5.08, m	122.8, CH	5.01, m	122.8, CH	5.01, m
7′	132.5, C		132.5, C		132.9, C		132.8, C	
8′	25.6, CH_3_	1.65, s	25.7, CH_3_	1.66, s	25.6, CH_3_	1.60, s	25.6, CH_3_	1.60, m
9′	17.7, CH_3_	1.57, s	17.7, CH_3_	1.57, s	17.6, CH_3_	1.54, s	17.6, CH_3_	1.53, m
10′	171.7, C		171.0, C		172.5, C		172.6, C	
1″	146.0, C		146.1, C		151.9, C		151.9, C	
2″	135.1, C		135.2, C		123.3, C		123.4, C	
3″	116.8, CH	6.72, d (3.0)	116.7, CH	6.71, d (3.1)	126.3, CH	6.74, d (2.0)	126.3, CH	6.73, d (1.6)
4″	151.7, C		151.6, C		146.8, C		146.8, C	
5″	113.6, CH	6.61, dd (8.7, 3.0)	113.5, CH	6.61, dd (8.7, 3.1)	124.1, CH	6.76,^b^ m	124.1, CH	6.75,^c^ s
6″	123.8, CH	6.87, d (8.7)	123.9, CH	6.88, d (8.7)	116.4, CH	6.76,^b^ m	116.4, CH	6.75,^c^ s

a The assignment of carbons may be interchanged.

b The aromatic proton signals were overlapped.

c The aromatic proton signals were overlapped.

The elemental formula of ganohochimin F (**6**) was determined as C_46_H_68_O_6_ by HRESIMS. It was identified as the 3β-hydroxy derivative of **5.**

Ganohochimin G (**7**) was assigned the molecular formula of C_46_H_66_O_6_ by HRESIMS. The NMR data for the lanostane unit were similar to those of ganohochimin C (**3**). The meroterpene unit was elucidated to be fornicin F (**10**). The chemical shifts of protons and carbons for the hydroquinone ring were similar to those of **3.**

The molecular formula of ganohochimin H (**8**) was determined as C_46_H_68_O_6_ based on the HRESIMS data. It was identified as the 3β-hydroxy derivative of **7.**

Several lanostane–meroterpene conjugates, including ganosinensins A–C,^13^ ganoleucoins M–P,^14^ ganoleucinins A–C,^15^ ganocalidoins A and B,^16^ and ganoleucocontins K and L,^17^ have been isolated from *Ganoderma*. They are conjugates of ester linkage by condensation of 24-OH or 26-OH of ganodermanontriol or its (24*R*,25*S*)-isomer with C-15 carboxylic acid of ganomycin B (**11**) or its modified variants. Ganohochimins represent unique aryl ether linkage patterns.

Plausible biosynthetic pathways for ganohochimins A (**1**) and C (**3**) are shown in Figure 5. A C-25 carbonium ion intermediate, generated from a 24,25-epoxy derivative, could be the precursor for the conjugates **1** (path *d*) and **3** (path *e*). Ganodermanondiol (**12**), one of the major metabolites, could be produced either from the 24,25-epoxide via path *c* or by some different pathway from a C-24/C-25 double bond derivative. Compound **12** could also serve as the source of the C-25 carbonium ion intermediate (reversed path *c*) in the production of the conjugates (**1** and **3**).

**Figure 5:**
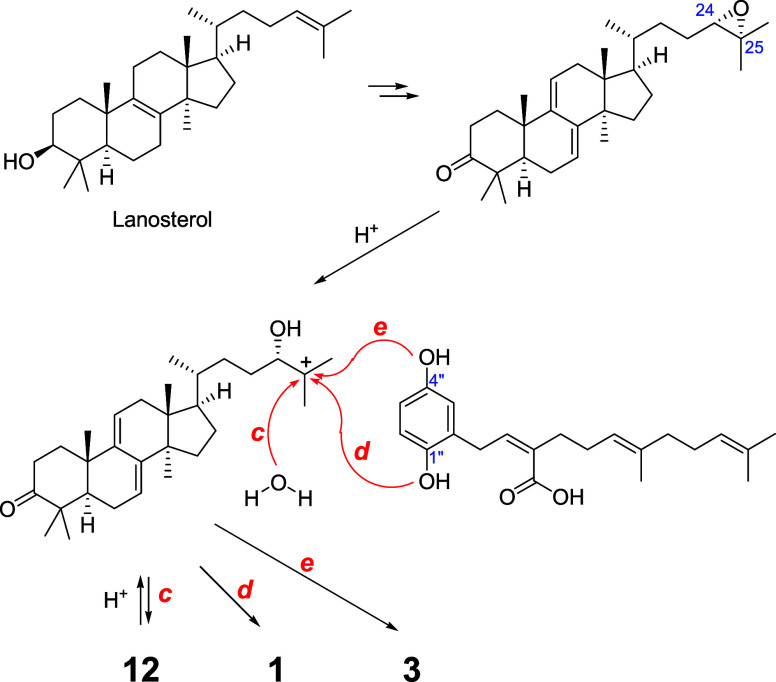
Plausible biosynthetic pathways for ganohochimins A (**1**) and C (**3**).

Ganohochimate A (**9**) was obtained as a colorless solid. The elemental formula was determined to be C_59_H_86_O_10_ based on the HRESIMS data. The NMR spectroscopic data of **9** (Table 3) suggested a lanostane dimer structure by condensation of different structural types of lanostane units. The structure was elucidated by analysis of the 2D NMR spectroscopic data (Figure 2) and comparison with those of the lanostane cometabolites. One of the lanostane units (C-1–C-30) of **9** was assigned to be ganodermanondiol (**12**). The other unit (C-1′–C-30′, lacking C-27′) was elucidated to be the same as the known 27-norlanostane, ganoboninone C (**38**),^18^ which is the 12α-hydroxy derivative of the cometabolite, ganoboninone E (**17**)^18,19^ (Figure S1). The ^1^H and ^13^C chemical shifts of the C-20–C-27 side chain were different between **9** and **12**. In particular, the oxymethine proton H-24 (δ_H_ 4.75, dd, *J* = 10.0, 2.5 Hz) of **9** was downfield shifted compared with **12** (δ_H_ 3.29, H-24). An ester linkage to condense the two lanostane units was revealed by the HMBC correlation from H-24 of ganodermanondiol to C-3′ (δ_C_ 173.0) of ganoboninone C. The NOESY correlations (Figure 6) were consistent with the same relative configurations of the two lanostane units with those of **12** and **38**. The structure of lanostane dimer **9** was confirmed by hydrolysis (Figure 7). Alkaline hydrolysis of **9** in 1 M aqueous NaOH – MeOH gave **12** and **38**, which also established the 24*S*-configuration of **9.**

**Table 3:** NMR Spectroscopic Data for Ganohochimate A (**9**) in CDCl_3_

no.	δ_C_, type	δ_H_, mult. (*J* in Hz)	nο.	δ_C_, type	δ_H_, mult. (*J* in Hz)
1	36.6, CH_2_	α 1.76, m; β2.28, m	1′	35.1, CH_2_	2.58, m; 2.01, m
2	34.8, CH_2_	α 2.34, m; β2.77, m	2′	30.1, CH_2_	2.42, m; 2.29, m
3	216.8, C		3′	173.0, C	
4	47.5, C		4′	58.0, C	
5	50.7, CH	1.53, dd (12.0, 3.7)	5′	47.2, CH	1.67, m
6	23.7, CH_2_	α 2.05, m; β 2.19, m	6′	37.4, CH_2_	2.80–2.77 (2H), m
7	120.0, CH	5.50, br d (6.5)	7′	196.3, C	
8	142.8, C		8′	152.8 C	
9	144.5, C		9′	150.0, C	
10	37.2, C		10′	38.7, C	
11	117.2, CH	5.37, d (5.7)	11′	205.0, C	
12	37.8, CH_2_	α 2.20, m; β 2.09, m	12′	78.5, CH	4.11, s
13	43.7, C		13′	63.1, C	
14	50.3, C		14′	45.9, C	
15	31.4, CH_2_	α 1.38, m; β 1.61, m	15′	34.0, CH_2_	α 2.28, m; β 2.03, m
16	27.9, CH_2_	1.97, m; 1.28, m	16′	23.8, CH_2_	1.94–1.91 (2H), m
17	50.7, CH	1.55, m	17′	46.5, CH	2.71, m
18	15.7, CH_3_	0.56, s	18′	76.4, CH_2_	α 3.35, d (9.2); β 3.47, d (9.2)
19	22.0, CH_3_	1.19, s	19′	20.5, CH_3_	1.28, s
20	36.4, CH	1.36, m	20′	86.9, C	
21	18.6, CH_3_	0.88, d (6.5)	21′	23.0, CH_3_	1.21, s
22	32.7, CH_2_	1.38, m; 1.00, m	22′	36.6, CH_2_	2.02, m; 1.78, m
23	26.5, CH_2_	1.72, m; 1.37, m	23′	37.6, CH_2_	2.57, m; 2.43, m
24	81.1, CH	4.75, dd (10.0, 2.5)	24′	211.6, C	
25	72.5, C		25′	36.1, CH_2_	2.46 (2H), m
26	26.8, CH_3_	1.20, s	26′	7.9, CH_3_	1.04, t (7.3)
27	24.9, CH_3_	1.20, s			
28	25.4, CH_3_	1.08, s	28′	57.1, CH_2_	2.69, d (4.3); 2.65, d (4.3)
29	22.4, CH_3_	1.12, s	29′	21.8, CH_3_	1.14, s
30	25.4, CH_3_	0.85, s	30′	28.6, CH_3_	1.12, s

**Figure 6:**
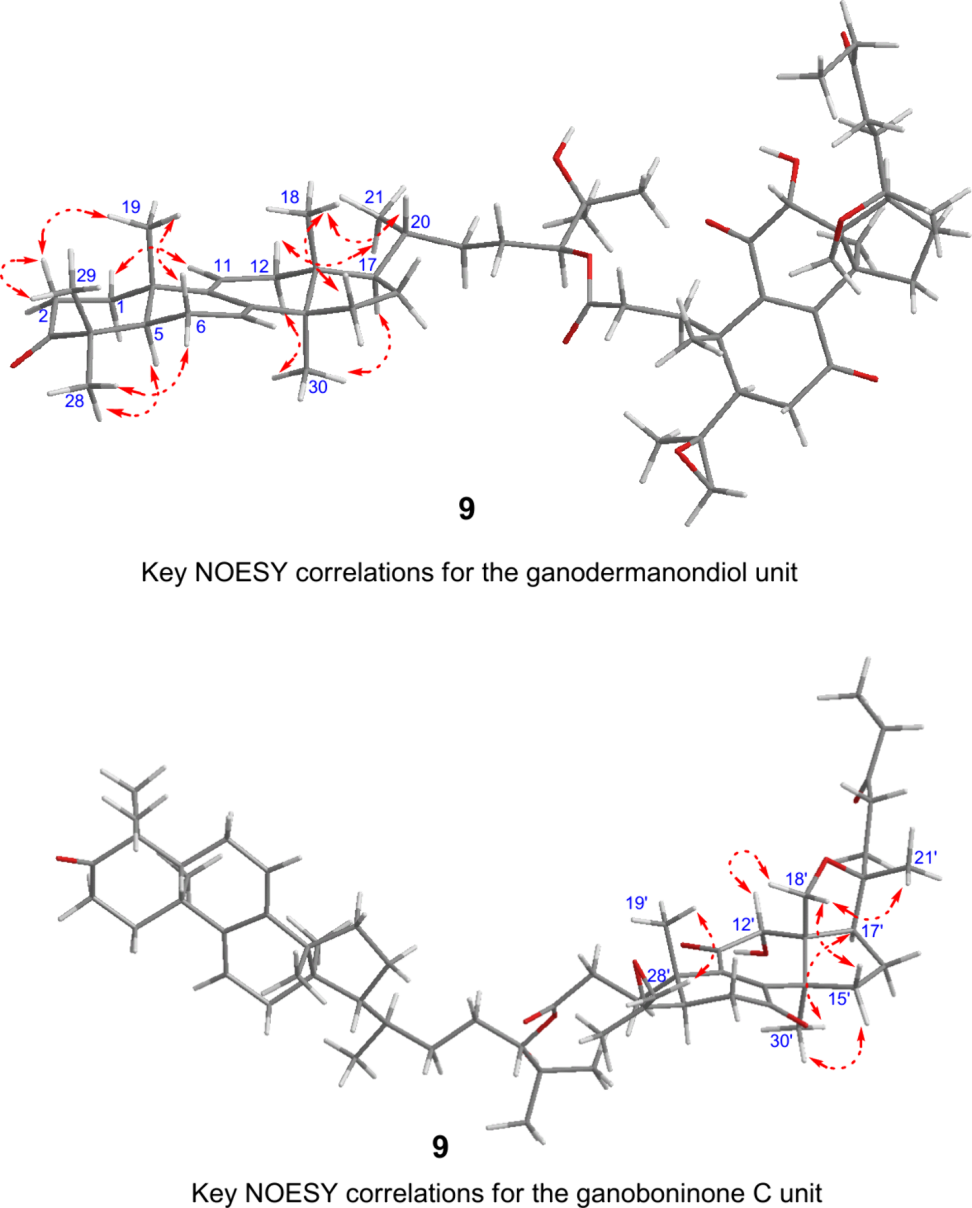
NOESY correlations for ganohochimate A (**9**).

**Figure 7:**
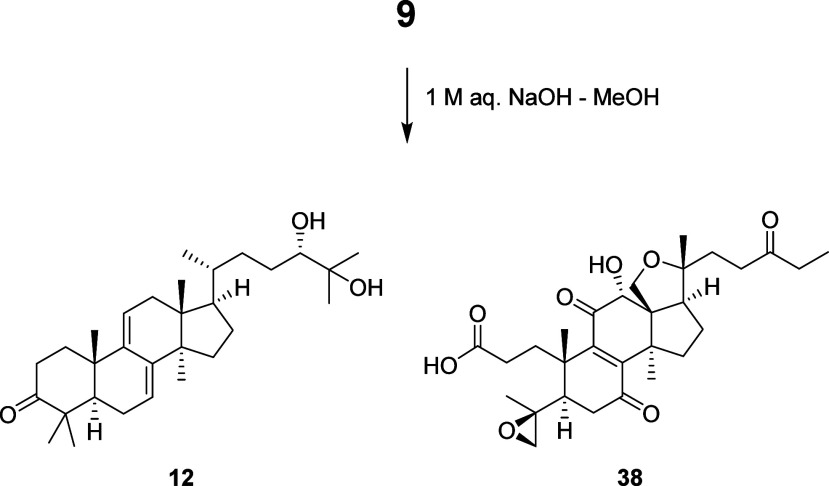
Alkaline hydrolysis of ganohochimate A (**9**).

Lanostane dimers are rare from *Ganoderma*. Ganoweberianones and isoganoweberianones, isolated from *G. weberianum*, are antimalarial lanostane dimers wherein either 24-OH or 26-OH of ganodermanontriol formed ester by condensation with C-26 carboxylic acid of another highly oxygenated C-30 lanostane.^20,21^ Recently, lanostane dimers linked by 3-hydroxy-3-methylglutaryl (HMG), ganoleucocontins M–R, were isolated from *G. leucocontextum*.^17^ The dimerization pattern of ganohochimate A (**9**) is different from these reported compounds.

In the quest for novel bioactive compounds with medicinal potentials from medicinal and edible mushroom sources in Thailand, we have been evaluating the isolated compounds for antitubercular, antimalarial, and antibacterial activitie.^20–22^ However, ganohochimins were inactive in the antitubercular (*Mycobacterium tuberculosis* H37Ra),^23^ antimalarial (*Plasmodium falciparum* K1), and antibacterial (*Bacillus cereus*, *Staphylococcus aureus* MSSA and MRSA, *Escherichia coli*, and *Salmonella* Typhi) activity assays. The isolated new compounds, **1**–**10**, were also evaluated for cytotoxicity to NCI-H187 (small-cell lung cancer) and MCF-7 (breast cancer) cell-lines and nonmalignant Vero cells (African green monkey kidney fibroblasts). Ganohochimins showed moderate to weak cytotoxicity to NCI-H187 cells, but, they were also cytotoxic to Vero cells (Table 4). Among these, ganohochimin F (**6**) showed lowest IC_50_ values: NCI-H187 cells, IC_50_ 7.7 μM; Vero cells, IC_50_ 6.3 μM. Ganoboninketals A–C and G–K, previously isolated from natural fruiting bodies of *G. cf. hochiminhense*­(collected from different location with the fungus used in this study), were shown to exhibit moderate antimalarial activity, but, they were noncytotoxic to Vero cells.^8^

## Conclusions

In conclusion, eight new lanostane–meroterpene conjugates, ganohochimins A–H (**1**–**8**), and a new lanostane dimer, ganohochimate A (**9**), were isolated from *G. cf. hochiminhense*. Ganohochimins possess unique aryl ether linkage between lanostane and meroterpene units, and they showed cytotoxic activities. Compound **9** is a new structural type of lanostane dimer. The present results demonstrate structural uniqueness and diversity of *Ganoderma* triterpenoids.

## Experimental Section

### General Experimental Procedures

Optical rotations were measured using a JASCO P-2000 digital polarimeter. UV spectra were obtained using a JASCO V-730 spectrophotometer. IR spectra were recorded on a Bruker ALPHA spectrometer. NMR spectra were acquired using Bruker Avance III HD 400 or 500 MHz spectrometer. High-resolution ESITOFMS measurements were performed on a Bruker micrOTOF mass spectrometer.

### Fungal Material

The natural mushroom specimen was collected from trunk of a dead oil palm *E. guineensis* in private plantation field, Khlong Thom District, Krabi Province, Thailand, in June 2006. The mushroom collection was deposited in the BIOTEC Bangkok Herbarium & Fungarium (code: BBH 19074). The culture was deposited in the Thailand Bioresource Research Center (strain code: TBRC-BCC 22084) on June 30, 2006. This fungus was recently identified to be *G. cf. hochiminhense*­(Polyporaceae) based on its gene sequence data of ITS rDNA (GenBank accession code: PP988496).

### Cultivation (Batch 1)

*G. cf. hochiminhense*TBRC-BCC 22084 was maintained on potato dextrose agar (PDA). The fungal colony was cut from the agar plate and transferred into a glass bottle containing sterile millet (7 × 17 cm), and incubated at 25 °C for 10 days. The spawn was inoculated into 120 cultivation plastic bags, each containing ca. 1 kg of substrate: composition, sawdust (92.5%), rice bran (4.6%), calcium sulfate (1.8%), calcium oxide (0.9%), and magnesium sulfate (0.2%). The substrate bags were incubated for mycelial growth in a cultivation house at 25 °C–30 °C for 31 days. Then, the caps on the top of the plastic bags were opened, and incubated additional 78 days. Fruiting bodies were induced from all substrate bags and were grown to suitable size (7–10 cm stalk long). They were harvested (picked) for chemical studies.

### Extraction and Isolation (Cultivation Batch 1)

Dried mushroom materials (0.79 kg) were cut into small pieces, and extracted with CH_2_Cl_2_ (6 L) at room temperature for 7 days. This extraction was repeated once to obtain the combined CH_2_Cl_2_ extract (22.3 g, brown gum). This extract was fractionated by column chromatography (CC) on silica gel (8 × 15 cm, EtOAc/hexane, step gradient elution 0:100, 20:80, 40:60, 60:40, 80:20, and 100:0, and then with 100% acetone) to obtain 19 fractions (Fr-1–Fr-19). The major components of Fr-1–Fr-7 (8.3 g in total) were lipids. Several known lanostanes were isolated by chromatographic fractionations of Fr-8–Fr-16 (6.4 g in total). Fr-17 (2.6 g) and Fr-18 (2.7 g) were subjected to preparative HPLC using an ODS column (Waters SunFire Prep C_18_ OBD, 19 × 250 mm, 10 μm; MeCN–H_2_O, gradient from 40:60 to 100:0 over 30 min, then, 100% MeCN for 30 min; flow rate 12 mL/min; room temperature; detection wavelength, 210 and 254 nm) to furnish **1** (13 mg), **2** (15 mg), **7** (3.7 mg), and **8** (3.9 mg). Fr-19 (1.2 g) was further fractionated by preparative HPLC (MeCN–H_2_O, gradient from 40:60 to 100:0, over 30 min) to furnish **10** (17 mg).

Known compounds **11**–**35** (Figure S1) were also isolated from the same CH_2_Cl_2_ extract. Their structures, isolated amounts, and corresponding references are shown in the Supporting Information (Figure S1).

### Cultivation (Batch 2), Extraction, and Isolation

The batch 2 cultivation was performed in a larger scale than batch 1, using 500 cultivation plastic bags. Dried fruiting bodies (1.75 kg) were extracted with CH_2_Cl_2_ (16 L, twice) at room temperature for 7 days. The combined CH_2_Cl_2_ extract (89.0 g) was subjected to silica gel CC (10 × 15 cm, EtOAc/hexane, step gradient elution 0:100, 20:80, 40:60, 60:40, 80:20, and 100:0, and then with 100% acetone) to obtain 14 fractions (Fr-1–Fr-14). The major components of Fr-1–Fr-5 (29 g in total) were lipids. Fr-6–Fr-9 (27 g in total).provided several known lanostanes. Fr-10 (6.5 g), Fr-11 (4.0 g), Fr-12 (3.1 g), and Fr-13 (5.2 g) were separated by preparative HPLC (Waters SunFire Prep C_18_ OBD, 19 × 250 mm, 10 μm; MeCN–H_2_O, gradient from 40:60 to 100:0, over 30 min, then, 100% MeCN for 50 min; flow rate 12 mL/min; room temperature; detection wavelength, 210 and 254 nm) to furnish **1** (20 mg), **2** (6.4 mg), **3** (41 mg), **4** (7.0 mg), **5** (22 mg), **6** (6.4 mg), **7** (3.7 mg), **8** (15 mg), and **9** (16 mg) (Figure S2). The preparative HPLC fractions were concentrated using rotary evaporators with gentle warming on a water bath (40–45 °C).

#### Ganohochimin A (**1**)

Colorless solid; [α]^25^_D_ + 23 (*c* 0.10, CHCl_3_); UV (MeOH) λ_max_ (log ε) 229 (4.39), 287 (3.54) nm; IR (ATR) ν_max_ 3396, 1701, 1493, 1376, 1214 cm^–1^; ^1^H NMR (500 MHz, CDCl_3_) and ^13^C NMR (125 MHz, CDCl_3_) spectroscopic data (Table S1); HRESIMS *m*/*z* 805.5374 [M + Na]^+^ (calcd for C_51_H_74_O_6_Na, 805.5378).

#### Ganohochimin B (**2**)

Colorless solid; [α]^25^_D_ + 28 (*c* 0.10, CHCl_3_); UV (MeOH) λ_max_ (log ε) 229 (4.32), 287 (3.60) nm; IR (ATR) ν_max_ 3402, 1707, 1462, 1376, 1214 cm^–1^; ^1^H NMR (500 MHz, CDCl_3_) and ^13^C NMR (125 MHz, CDCl_3_) spectroscopic data (Table S1); HRESIMS *m*/*z* 807.5536 [M + Na]^+^ (calcd for C_51_H_76_O_6_Na, 807.5534).

#### Ganohochimin C (**3**)

Colorless solid; [α]^26^_D_ + 28 (*c* 0.10, CHCl_3_); UV (MeOH) λ_max_ (log ε) 227 (4.46), 234 (4.45), 243 (4.41), 252 (4.22), 287 (3.65) nm; IR (ATR) ν_max_ 3310, 1693, 1495, 1375, 1237, 1138 cm^–1^; ^1^H NMR (500 MHz, CDCl_3_) and ^13^C NMR (125 MHz, CDCl_3_) spectroscopic data (Table S1); HRESIMS *m*/*z* 805.5375 [M + Na]^+^ (calcd for C_51_H_74_O_6_Na, 805.5378).

#### Ganohochimin D (**4**)

Colorless solid; [α]^26^_D_ + 34 (*c* 0.10, CHCl_3_); UV (MeOH) λ_max_ (log ε) 227 (4.39), 234 (4.38), 243 (4.34), 252 (4.16), 286 (3.58) nm; IR (ATR) ν_max_ 3374, 1687, 1495, 1445, 1373, 1237, 1139 cm^–1^; ^1^H NMR (500 MHz, CDCl_3_) and ^13^C NMR (125 MHz, CDCl_3_) spectroscopic data (Table S1); HRESIMS *m*/*z* 807.5532 [M + Na]^+^ (calcd for C_51_H_76_O_6_Na, 807.5534).

#### Ganohochimin E (**5**)

Colorless solid; [α]^26^_D_ + 27 (*c* 0.10, CHCl_3_); UV (MeOH) λ_max_ (log ε) 228 (4.37), 234 (4.38), 243 (4.33), 251 (4.14), 287 (3.54) nm; IR (ATR) ν_max_ 3376, 1694, 1493, 1450, 1375, 1215, 1138 cm^–1^; ^1^H NMR (500 MHz, CDCl_3_) and ^13^C NMR (125 MHz, CDCl_3_) spectroscopic data (Table S2); HRESIMS *m*/*z* 737.4755 [M + Na]^+^ (calcd for C_46_H_66_O_6_ Na,737.4752).

#### Ganohochimin F (**6**)

Colorless solid; [α]^26^_D_ + 25 (*c* 0.10, CHCl_3_); UV (MeOH) λ_max_ (log ε) 228 (4.28), 234 (4.28), 243 (4.24), 252 (4.05), 288 (3.46) nm; IR (ATR) ν_max_ 3347, 1687, 1493, 1446, 1373, 1215 cm^–1^; ^1^H NMR (500 MHz, CDCl_3_) and ^13^C NMR (125 MHz, CDCl_3_) spectroscopic data (Table S2); HRESIMS *m*/*z* 739.4901 [M + Na]^+^ (calcd for C_46_H_68_O_6_Na, 739.4908).

#### Ganohochimin G (**7**)

Colorless solid; [α]^25^_D_ + 29 (*c* 0.10, CHCl_3_); UV (MeOH) λ_max_ (log ε) 222 (4.40), 286 (3.64) nm; IR (ATR) ν_max_ 3382, 1706, 1376 cm^–1^; ^1^H NMR (500 MHz, CDCl_3_) and ^13^C NMR (125 MHz, CDCl_3_) spectroscopic data (Table S2); HRESIMS *m*/*z* 737.4755 [M + Na]^+^ (calcd for C_46_H_66_O_6_Na, 737.4752).

#### Ganohochimin H (**8**)

Colorless solid; [α]^25^_D_ + 40 (*c* 0.10, CHCl_3_); UV (MeOH) λ_max_ (log ε) 222 (4.47), 286 (3.67) nm; IR (ATR) ν_max_ 3405, 1699, 1374 cm^–1^; ^1^H NMR (500 MHz, CDCl_3_) and ^13^C NMR (125 MHz, CDCl_3_) spectroscopic data (Table S2); HRESIMS *m*/*z* 739.4913 [M + Na]^+^ (calcd for C_46_H_68_O_6_Na, 739.4908).

#### Ganohochimate A (**9**)

Colorless solid; [α]^26^_D_ + 35 (*c* 0.10, CHCl_3_); UV (MeOH) λ_max_ (log ε) 235 (4.31), 243 (4.33), 252 (4.22), 271 (3.87) nm; IR (ATR) ν_max_ 3427, 1707, 1676, 1455, 1376, 1175, 1111, 735 cm^–1^; ^1^H NMR (500 MHz, CDCl_3_) and ^13^C NMR (125 MHz, CDCl_3_) spectroscopic data (Table S3); HRESIMS *m*/*z* 977.6115 [M + Na]^+^ (calcd for C_59_H_86_O_10_Na, 977.6113).

#### Fornicin F (**10**)

Colorless gum; UV (MeOH) λ_max_ (log ε) 266 (3.84) nm; IR (ΑΤΡ) ν_max_ 3294, 1684, 1636, 1451, 1231, 1203 cm^–1^; ^1^H NMR (400 MHz, CDCl_3_) δ 6.73 (1H, d, *J* = 8.4 Hz, H-6′), 6.64 (1H, d, *J* = 3.0 Hz, H-3′), 6.62 (1H, dd, *J* = 8.4, 3.0 Hz, H-5′), 6.01 (1H, t, *J* = 8.7 Hz, H-2), 5.02 (1H, m, H-6), 3.65 (2H, d, *J* = 8.7 Hz, H-1), 2.31 (2H, m, H-4), 2.13 (2H, m, H-5), 1.62 (3H, s, H-8), 1.54 (3H, s, H-9); ^13^C NMR (100 MHz, CDCl_3_) δ 173.3 (C, C-10), 149.1 (C, C-1′), 148.9 (C, C-4′), 141.5 (CH, C-2), 132.9 (C, C-7), 130.8 (C, C-3), 124.1 (C, C-2′), 122.8 (CH, C-6), 117.1 (CH, C-3′), 116.9 (CH, C-6′), 114.9 (CH, C-5′), 34.4 (CH_2_, C-4), 31.8 (CH_2_, C-1), 27.4 (CH_3_, C-5), 25.5 (CH_3_, C-8), 17.6 (CH_3_, C-9); HRESIMS *m*/*z* 299.1258 [M + Na]^+^ (calcd for C_16_H_20_O_4_Na, 299.1254).

### Acidic Degradation of Ganohochimin A (**1**)

To a solution of ganohochimin A (**1**, 2.0 mg) in CDCl_3_ (0.5 mL) was added a saturated solution of *p*-TsOH·H_2_O in CDCl_3_ (0.5 mL) in a NMR sample tube. The solution was analyzed by ^1^H NMR, which suggested slow conversion of **1**. After standing for 2 days, the solution was poured into a saturated aqueous NaHCO_3_ (0.5 mL), and partially concentrated using a rotary evaporator. The residue was diluted with saturated aqueous NaHCO_3_ (2 mL) and extracted with EtOAc (2 × 4 mL). The combined EtOAc solution was concentrated using a rotary evaporator to obtain a pale brown gum, which was subjected to preparative HPLC (VDS optilab, VDSpher PUR 100 C18-E, 10 × 250 mm, 10 mm; MeCN–H_2_O, 60:40; flow rate 4 mL/min) to furnish **36** (0.4 mg, 36%) and **37** (0.5 mg, 45%).

#### 3,24-Dioxolanosta-7,9­(11)-diene (**37**)

Colorless solid; ^1^H NMR (500 MHz, CDCl_3_) δ 5.51 (1H, br d, *J* = 6.6 Hz, H-7), 5.38 (1H, br d, *J* = 6.2 Hz, H-11), 2.77 (1H, m, H_β_-1), 2.62 (1H, m, H-25), 2.49 (1H, m, H_a_-23), 2.38 (1H, m, H_b_-23), 2.34 (1H, m, H_α_-2), 2.28 (1H, m, H_β_-1), 2.22 (1H, m, H_α_-12), 2.20 (1H, m, H_β_-6), 2.10 (1H, m, H_β_-12), 2.04 (1H, m, H_α_-6), 2.02 (1H, m, H_a_-16), 1.77 (1H, m, H_a_-22), 1.76 (1H, m, H_α_-1), 1.63 (1H, m, H_β_-15), 1.56 (1H, m, H-17), 1.53 (1H, dd, *J* = 12.3, 3.7 Hz, H-5), 1.40 (1H, m, H-20), 1.38 (2H, m, H_α_-15 and H_b_-16), 1.25 (1H, m, H_b_-22), 1.19 (3H, s, H-19), 1.12 (3H, s, H-29), 1.09 (6H, d, *J* = 6.9 Hz, H-26 and H-27), 1.08 (3H, s, H-28), 0.89 (3H, d, *J* = 6.1 Hz, H-21), 0.87 (3H, s, H-30), 0.58 (3H, s, H-18); ^13^C NMR (125 MHz, CDCl_3_) δ 216.7 (C, C-3), 215.3 (C, C-24), 144.6 (C, C-9), 142.8 (C, C-8), 120.0 (CH, C-7), 117.2 (CH, C-11), 51.0 (CH, C-17), 50.7 (CH, C-5), 50.3 (C, C-14), 47.5 (C, C-4), 43.8 (C, C-13), 40.9 (CH, C-25), 37.8 (CH_2_, C-12), 37.5 (CH_2_, C-23), 37.2 (C, C-10), 36.6 (CH_2_, C-1), 35.9 (CH, C-20), 34.8 (CH2, C-2), 31.5 (CH_2_, C-15), 30.1 (CH_2_, C-22), 27.8 (CH_2_, C-16), 25.4 (CH_3_, C-28), 25.4 (CH_3_, C-30), 23.7 (CH_2_, C-6), 22.5 (CH_3_, C-29), 22.1 (CH_3_, C-19), 18.4 (CH_3_, C-21), 18.3 (CH_3_, C-26), 18.3 (CH_3_, C-27), 15.7 (CH_3_, C18); HRESIMS *m*/*z* 461.3392 [M + Na]^+^ (calcd for C_30_H_46_O_2_Na, 461.3390).

### Alkaline Hydrolysis of Ganohochimate A (**9**)

Ganohochimate A (**9**, 1.0 mg) was hydrolyzed in 1 M aqueous NaOH (50 μL) – MeOH (0.5 mL) at room temperature for 30 min. The mixture was acidified with 1 M HCl (0.1 mL) and partially concentrated using a rotary evaporator. The residue was dissolved in EtOAc and washed with H_2_O. The organic phase was concentrated using a rotary evaporator. The residual pale yellow gum was separated by preparative HPLC (Waters SunFire Prep C18 OBD, 19 × 150 mm, 5 μm; MeCN–H_2_O, gradient from 60:40 to 100:0 over 30 min, then, 100% MeCN for 10 min; flow rate 6 mL/min) to furnish ganodermanondiol (**12**, 0.5 mg, *t*_R_ 30.4 min) and ganoboninone C (**38**, 0.3 mg, *t*_R_ 4.5 min). The ^1^H NMR (pyridine-*d*_5_) spectroscopic data and HRESIMS data of the reaction product **38** were consistent with the literatura data for ganoboninone C.

### Biological Assays

The cytotoxic activities against NCI–H187 (ATCC CRL-5804) and MCF-7 (ATCC HTC-22) cells were performed using the resazurin fluorescent method.^23^ The cytotoxicity to Vero cells (ATCC CCL-81) was evaluated using the green fluorescent protein detection method.^24^ The antimycobacterial activity against *M. tuberculosis* H37Ra was also performed using the green fluorescent protein detection method.^25^ Rifampicin (MIC 0.025 μg/mL) and isoniazid (MIC 0.375 μg/mL) were used as standard drugs. The microculture radioisotope technique was used for the antiplasmodial assay against *P. falciparum* K1.^26^ Dihydroartemisinin (IC_50_ 1.4 nM) and chloroquine (IC_50_ 0.58 μM) were used as standard drugs. Antibacterial activity was determined in duplicate using the resazurin microdilution assay.^27^ Tetracycline (250 μg/mL) was used as positive control.

**Table 4:** Cytotoxic Activities of Compounds **1**–**10**

compound	NCI-H187 IC_50_, μM	MCF-7 IC_50_, μM	Vero IC_50_, μM
ganohochimin A (**1**)	19	>64	17
ganohochimin B (**2**)	33	>64	28
ganohochimin C (**3**)	21	>64	30
ganohochimin D (**4**)	16	>64	28
ganohochimin E (**5**)	21	>70	13
ganohochimin F (**6**)	7.7	>70	6.3
ganohochimin G (**7**)	21	>70	14
ganohochimin H (**8**)	18	>70	24
ganohochimate A (**9**)	>52	>52	52
fornicin F (**10**)	134	>181	>181
doxorubicin^a^	0.15	11	
tamoxifen^a^		36	
ellipticine^a^	7.6		6.0

a Standard compounds for the cytotoxicity assay.

## Supplementary Material



## Notes

1D and 2D NMR and HRESIMS spectra of the new compounds **1**–**10** and **37** (PDF)
