# Supplementary material for: Lanostane–Meroterpene Conjugates with Unusual Aryl Ether Linkage and a Lanostane Dimer from Artificially Cultivated Fruiting Bodies of Ganoderma cf. hochiminhense
Source: ACS Omega. 2025 Oct 13;10(41):48818–28. doi: 10.1021/acsomega.5c06826 (PMC12547551; doi:10.1021/acsomega.5c06826)

Supporting Information

Lanostane-Meroterpene Conjugates with Unusual Aryl Ether Linkages and a Lanostane Dimer from Artificially Cultivated Fruiting Bodies of *Ganoderma* cf. *hochiminhense*

Malipan Sappan,[†] Panida Chinthanom,[†] Kitlada Srichomthong,[†] Tuksaporn Thummarukcharoen,[‡] Rattaket Choeyklin,[‡] Aphidech Sangdee,[§] and Masahiko Isaka*,[†]

[†]National Center for Genetic Engineering and Biotechnology (BIOTEC), National Science and Technology Development Agency (NSTDA), 111 Thailand Science Park, Phahonyothin Road, Klong Luang, Pathumthani 12120, Thailand

[‡]National Biobank of Thailand, National Science and Technology Development Agency (NSTDA), 111 Thailand Science Park, Phahonyothin Road, Klong Luang, Pathumthani 12120, Thailand

[§]Department of Biology, Faculty of Science, Khamriang, Kantarawichai, Mahasarakham University, Maha Sarakham 44150, Thailand

* Corresponding author. *E-mail address:*isaka@biotec.or.th

Figure S1. Structures of the compounds isolated from fruiting bodies of *Ganoderma* cf. *hochiminhense* (Cultivation Batch 1). Isolated amounts (mg) are shown in parentheses. References for the known compounds (**11–35**) are listed below.

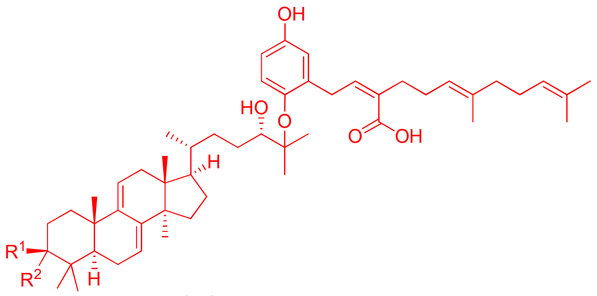

1 R^1,R^2 = O (13 mg)
2 R^1 = OH, R^2 = H (15 mg)

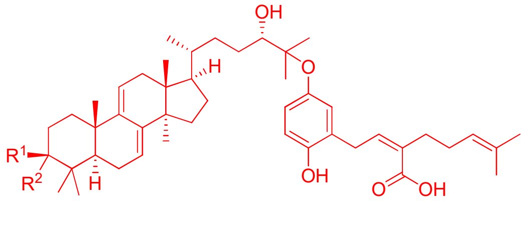

7 R^1,R^2 = O (3.7 mg)
8 R^1 = OH, R^2 = H (3.9 mg)

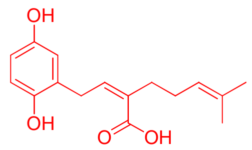

10 (17 mg)

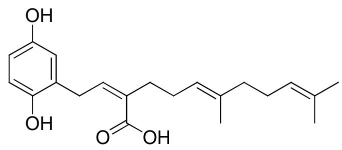

11 : ganomycin B (246 mg)

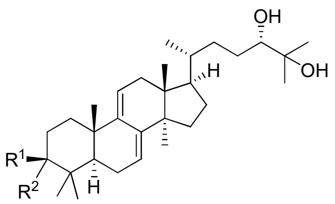

12 R^1,R^2 = O : ganodermanondiol (1.58 g)
13 R^1 = OH, R^2 = H : lucidumol B (714 mg)

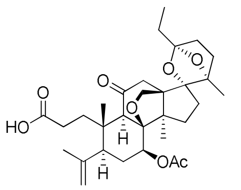

14 : ganoboninketal D (836 mg)

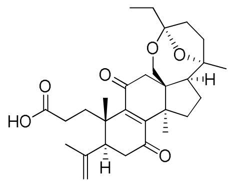

15 : ganoboninketal E (777 mg)

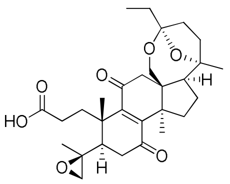

16 : ganoboninketal F (768 mg)

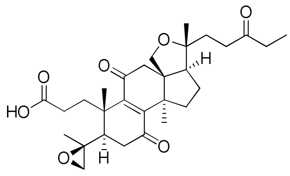

17 : ganoboninone E (630 mg)

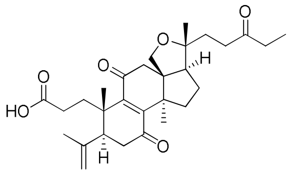

18 : ganoboninone G (219 mg)

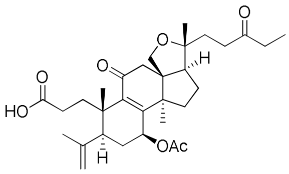

19 : ganoboninone H (13 mg)

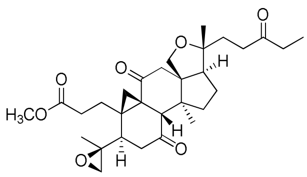

20 : ganorbifateD (18 mg)

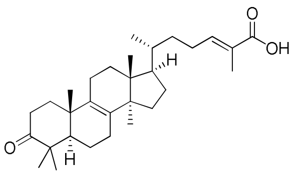

21 : (24*E*)-3-oxolanosta-8,24-dien -26-oic acid (68 mg)

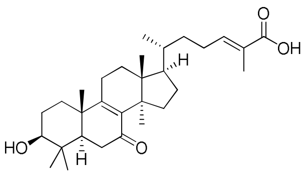

22 : 7-oxo-ganoderic acid Z (15 mg)

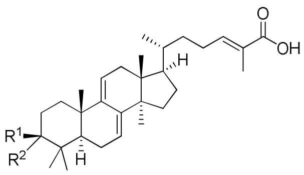

23 R^1,R^2 = O : (24*E*)-3-oxolanosta -7,9(11),24-trien-26-oic acid (20 mg)
24 R^1 = OH, R^2 = H : ganoderic acid Y (11 mg)

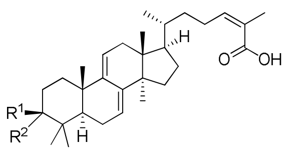

25 R^1,R^2 = O : ganoderic acid Sz (43 mg)
26 R^1 = OH, R^2 = H : (24*Z*)-3β -hydroxylanosta -7,9(11),24 -trien-26-oic acid (14 mg)

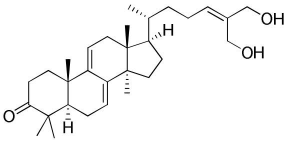

27 : ganoderiol F (2.1 mg)

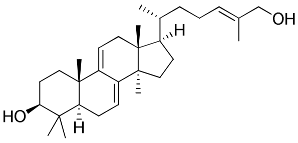

28 : ganodermadiol (17 mg)

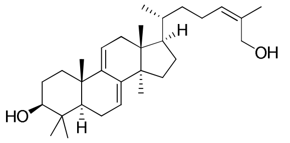

29 : (24*Z*)-3β,27-dihydroxylanosta -7,9(11),24-triene (16 mg)

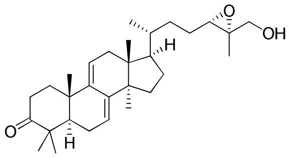

30 : epoxyganoderiol B (8.3 mg)

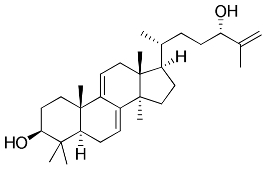

31 : (24*S*)-3β,24-dihydroxylanosta -7,9(11),25-triene (13 mg)

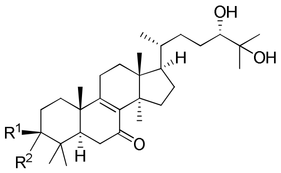

32 R_1,R^2 = O : lucidumol A (105 mg)
33 R^1 = OH, R^2 = H : ganoderitriol M (99 mg)

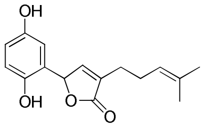

34 : fornicin A (29 mg)

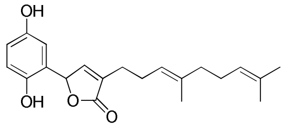

35 : ganomycin I (21 mg)

Figure S2. Structures of the new compounds isolated from fruiting bodies of *Ganoderma* cf. *hochiminhense* (Cultivation Batch 2). Isolated amounts (mg) are shown in parentheses.

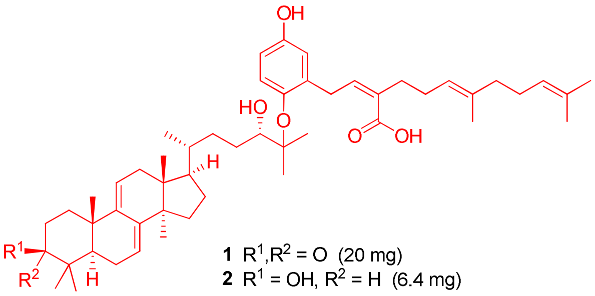

1 R^1,R^2 = O (20 mg)
2 R^1 = OH, R^2 = H (6.4 mg)

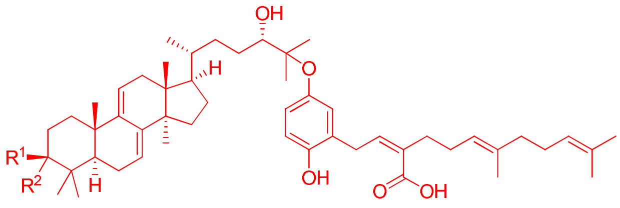

3 R^1,R^2 = O (41 mg)
4 R^1 = OH, R^2 = H (7.0 mg)

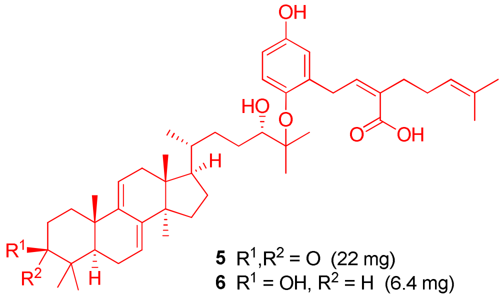

5 R^1,R^2 = O (22 mg)
6 R^1 = OH, R^2 = H (6.4 mg)

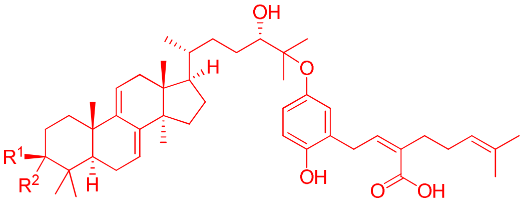

7 R^1,R^2 = O (3.7 mg)
8 R^1 = OH, R^2 = H (15 mg)

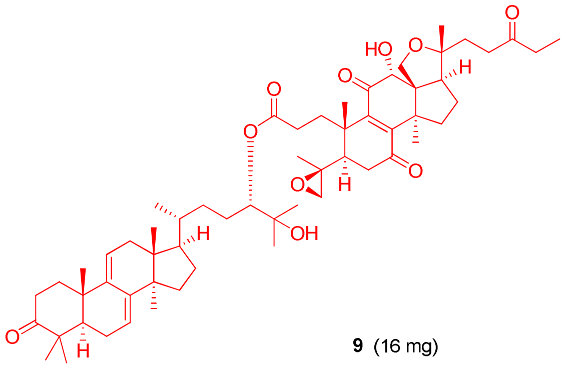

9 (16 mg)

References for the known compounds

11 (ganomycin B) : Mothana, R. A. A.; Jansen, R.; Jülich, W.-D.; Lindequist, U. Ganomycins A and B, new antimicrobial hydroquinones from the basidiomycete *Ganoderma pfeifferi*. *J. Nat. Prod.* **2000**, *63*, 416–418.

12 (ganodermanondiol) : Fujita, A.; Arisawa, M.; Saga, M.; Hayashi, T.; Morita, N. Two new lanostanoids from *Ganoderma lucidum*. *J. Nat. Prod.* **1986**, *49*, 1122–1125.

13 (lucidumol B) : Min, B.-S.; Nakamura, N.; Miyashiro, H.; Bae, K.-W.; Hattori, M. Triterpenes from the spores of *Ganoderma lucidum* and their inhibitory activity against HIV-1 protease. *Chem. Pharm. Bull.* **1998**, *46*, 1607–1612.

14 (ganoboninketal D) : Isaka, M.; Chinthanom, P.; Mayteeworakoon, S.; Laoteng, K.; Suvannakad, R.; Choeyklin, R. Lanostane triterpenoids from cultivated fruiting bodies of the basidiomycete *Ganoderma orbiforme*. *Phytochem. Lett.* **2017**, *21*, 251–255.

15 (ganoboninketal E) : Li, W.; Chinthanom, P.; Rachtawee, P.; Intereya, K.; Feng, T.; Liu, J. K.; Isaka, M. Isolation of 3,4-seco-27-norlanostane triterpenoids from cultivated fruiting bodies of *Ganoderma orbiforme*. *Phytochem. Lett.* **2018**, *28*, 104–109.

16 (ganoboninketal F) : Li, W.; Chinthanom, P.; Rachtawee, P.; Intereya, K.; Feng, T.; Liu, J. K.; Isaka, M. Isolation of 3,4-seco-27-norlanostane triterpenoids from cultivated fruiting bodies of *Ganoderma orbiforme*. *Phytochem. Lett.* **2018**, *28*, 104–109.

17 (ganoboninone E) : Ma, K.; Li, L.; Bao, L.; He, L.; Sun, C.; Zhou, B.; Si, S.; Liu, H. Six new 3,4-seco-27-norlanostane triterpenes from the medicinal mushroom *Ganoderma boninense* and their antiplasmodial activity and agonistic activity to LXRβ. *Tetrahedron* **2015**, *71*, 1808–1814**.**

18 (ganoboninone G) : Li, W.; Chinthanom, P.; Rachtawee, P.; Intereya, K.; Feng, T.; Liu, J.-K.; Isaka, M. Isolation of 3,4-seco-27-norlanostane triterpenoids from cultivated fruiting bodies of *Ganoderma orbiforme*. *Phytochem. Lett.* **2018**, *28*, 104–109.

19 (ganoboninone H) : Li, W.; Chinthanom, P.; Rachtawee, P.; Intereya, K.; Feng, T.; Liu, J.-K.; Isaka, M. Isolation of 3,4-seco-27-norlanostane triterpenoids from cultivated fruiting bodies of *Ganoderma orbiforme*. *Phytochem. Lett.* **2018**, *28*, 104–109.

20 (ganorbifate D) : Yin, X.; Yu, C.; Tuong, T. M. L., Kou, R.-W.; Yang, A.-A.; Chen, X.; Wang, W. J.; Gao, Y.-Q.; Gao, J.-M. Structures of ganorbifates C–I, seven previously undescribed lanostanoids from the mushroom *Ganoderma orbiforme*, and insights of computed biosynthesis with DFT. *Phytochemistry* **2022**, *194*, 113004.

21 : Chen, Y. G.; Song, X. P.; Hai, L. N.; Fang, A.; Bi, Y. M.; Liao, X. R. Triterpenoid acids from *Kadsura ananosma*. *Polish J. Chem.* **2006**, *80*, 1677–1681.

22 (7-oxo-ganoderic acid Z) : Li, C.; Li, Y.; Sun, H. H. New ganoderic acids, bioactive triterpenoid metabolites from *Ganoderma lucidum*. *Nat. Prod. Res.* **2006**, *20*, 985–991.

23 : Morigiwa, A.; Kitabatake, K.; Fujimoto, Y.; Ikekawa, N. Angiotensin converting enzyme-inhibitory triterpenoids from *Ganoderma lucidum*. *Chem. Pharm. Bull.* **1986**, *34*, 3025–3028.

24 (ganoderic acid Y) : Lin, L. J.; Shao, M. S.; Yeh, S. F. Seven new triterpenes from *Ganoderma lucidum*. *J. Nat. Prod.* **1988**, *51*, 918–924.

25 (ganoderic acid Sz) : Li, C.; Yin, J.; Guo, F.; Zhang, D.; Sun, H. H. Ganoderic acid Sz, a new lanostanoid from the mushroom *Ganoderma lucidum*. *Nat. Prod. Res.* **2005**, *19*, 461–465.

26 : Yangchum, A.; Rachtawee, P.; Srichomthong, K.; Choeyklin, R.; Boonpratuang, T.; Thongpanchang, C.; Isaka, M. Lanostane triterpenoids from artificially cultivated fruiting bodies of *Ganoderma* cf. *mastoporum*. *Nat. Prod. Res.* **2024**, *38*, 2644–2652.

27 (ganoderiol F) : Nishitoba, T.; Oda, K.; Sato, H.; Sakamura, S. Novel triterpenoids from the fungus *Ganoderma lucidum*. *Agric. Biol. Chem.* **1988**, *52*, 367–372.

28 (ganodermadiol) : Arisawa, M.; Fujita, A.; Saga, M.; Fukumura, H.; Hayashi, T.; Shimizu, M.; Morita, N. Three new lanostanoids from *Ganoderma lucidum*. *J. Nat. Prod.* **1986**, *49*, 621–625.

29 : Li, W.; Chinthanom, P.; Rachtawee, P.; Intereya, K.; Feng, T.; Liu, J. K.; Isaka, M. Isolation of 3,4-seco-27-norlanostane triterpenoids from cultivated fruiting bodies of *Ganoderma orbiforme*. *Phytochem. Lett.* **2018**, *28*, 104–109.

30 (epoxyganoderiol B) : Nishitoba, T.; Sato, H.; Oda, K.; Sakamura, S. Novel triterpenoids and a steroid from a fungus *Ganoderma lucidum*. *Agric. Biol. Chem.* **1988**, *52*, 211–216.

31 : Isaka, M.; Sappan, M.; Choowong, W.; Boonpratuang, T.; Choeyklin, R.; Feng, T.; Liu, J. K. Antimalarial lanostane triterpenoids from cultivated fruiting bodies of the basidiomycete *Ganoderma* sp. *J. Antibiot.* **2020**, *73*, 702–710.

32 (lucidumol A) : Min, B.-S.; Nakamura, N.; Miyashiro, H.; Bae, K.-W.; Hattori, M. Triterpenes from the spores of *Ganoderma lucidum* and their inhibitory activity against HIV-1 protease. *Chem. Pharm. Bull.* **1998**, *46*, 1607–1612.

33 (ganoderitriol M) : Chen, M.; Zhang, M.; Sun, S.; Xia, B.; Zhang, H. Q. A new triterpene from fruiting bodies of *Ganoderma lucidum*. Yao Xue Xue Bao **2009**, *44*, 768–770.

34 (fornicin A) : Niu, X. M.; Li, S. H.; Sun, H. D.; Che, C. T. Prenylated phenolics from *Ganoderma fornicatum*. *J. Nat. Prod*. **2006**, *69*, 1364–1365.

35 (ganomycin I) : El Dine, R. S.; El Halawany, A. M., Ma, C. M.; Hattori, M. Inhibition of the dimerization and active site of HIV-1 protease by secondary metabolites from the Vietnamese mushroom *Ganoderma colossum*. *J. Nat. Prod*. **2009**, *72*, 2019–2023.

Figure S3. COSY and HMBC correlations for compounds **2–8** and **10**.

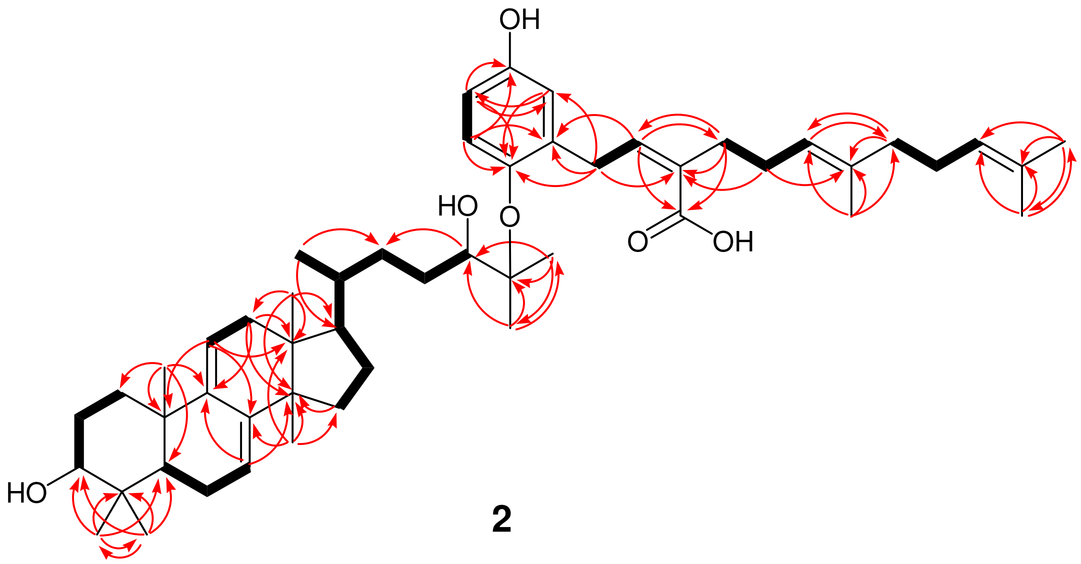

2

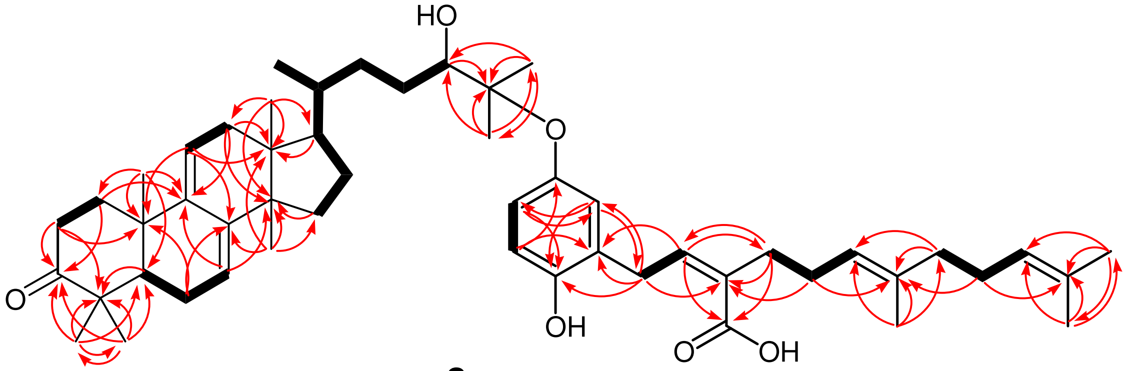

3

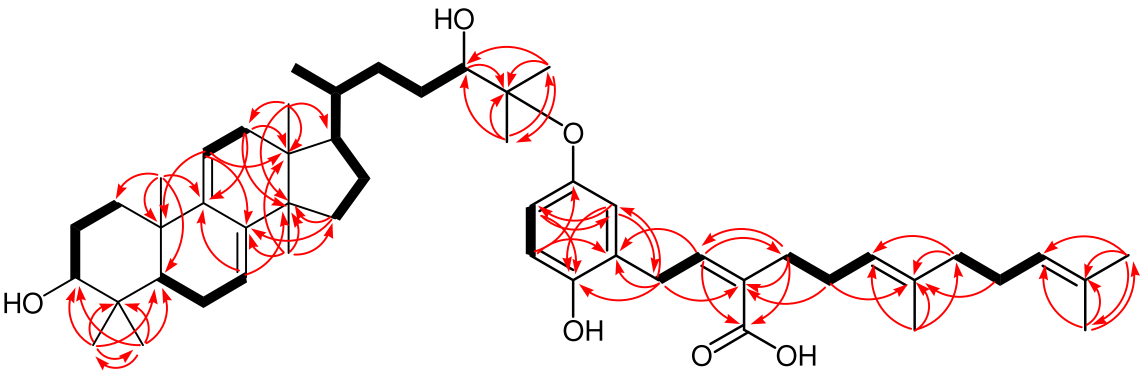

4

COSY
HMBC

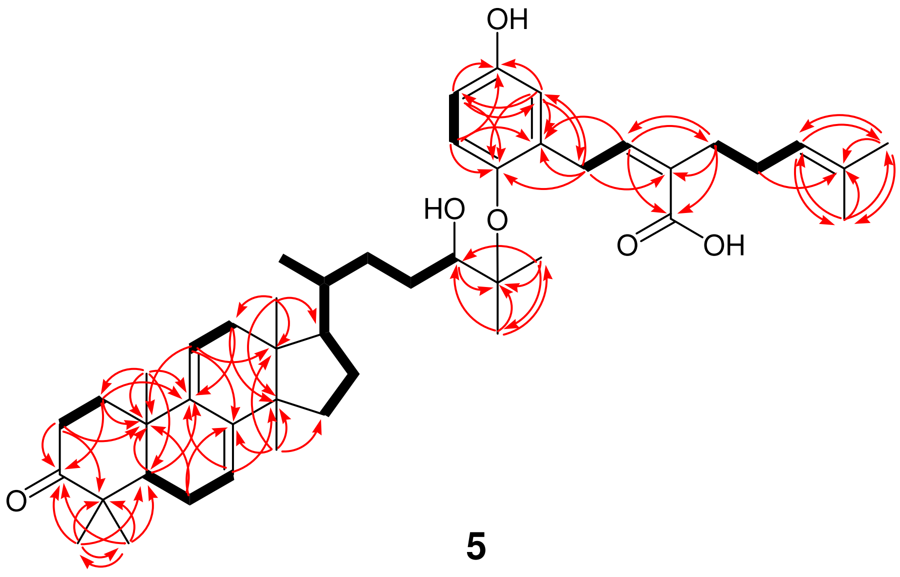

5

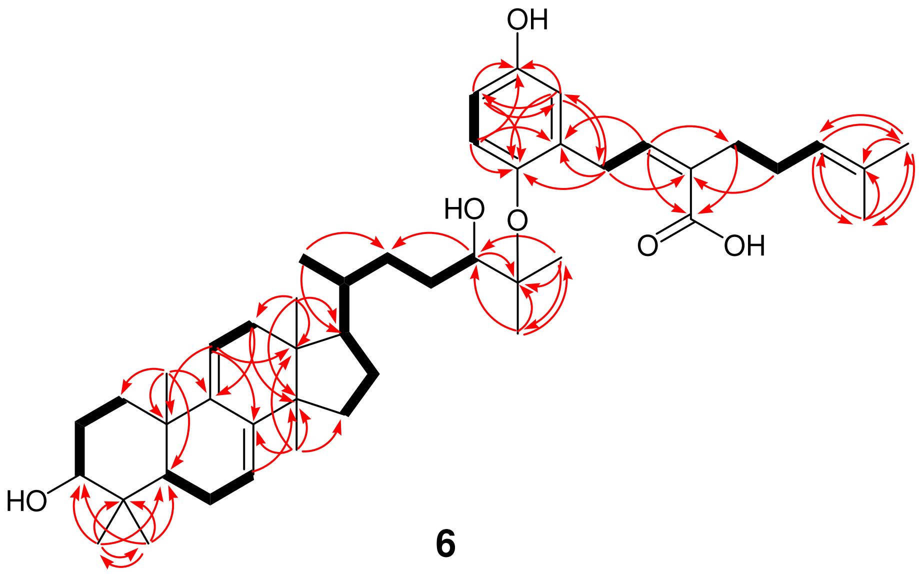

6

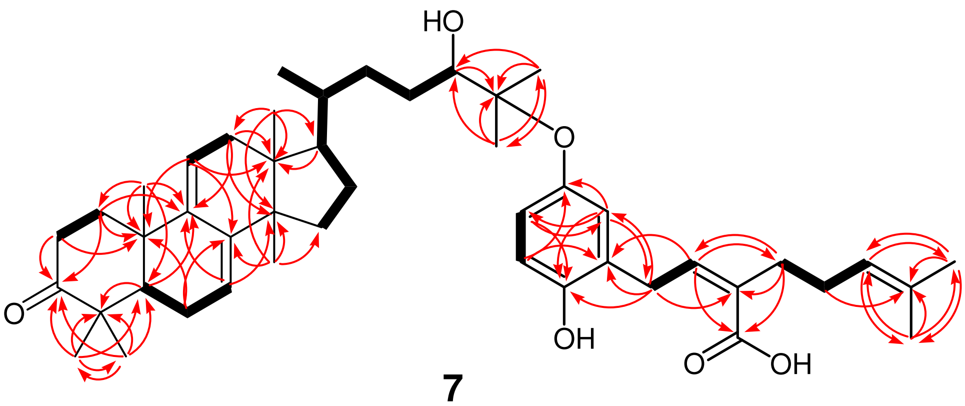

7

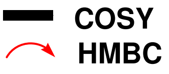

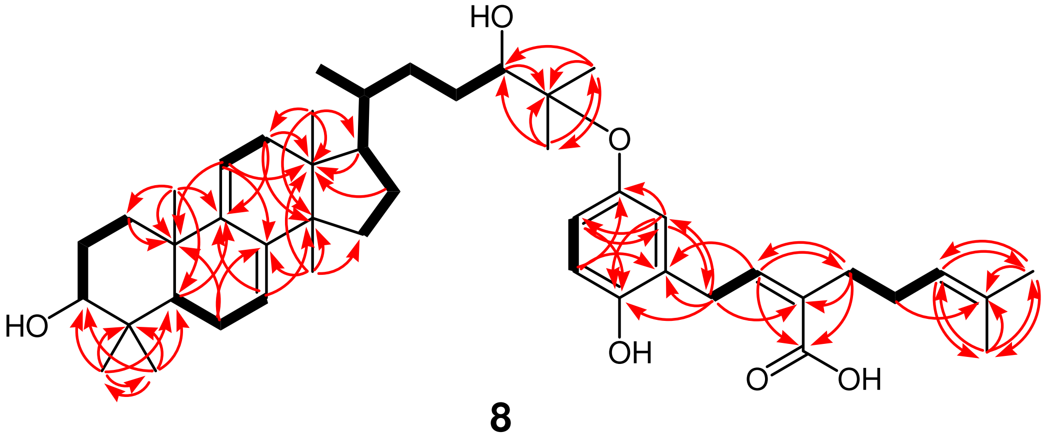

8

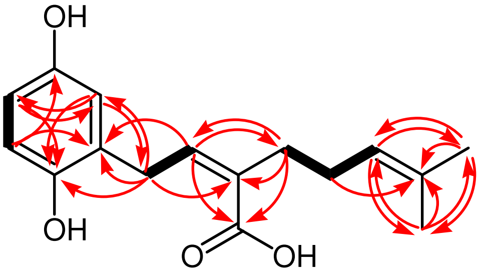

10

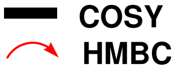

Figure S4. Key NOESY correlations for **2**, **5**, and **10**.

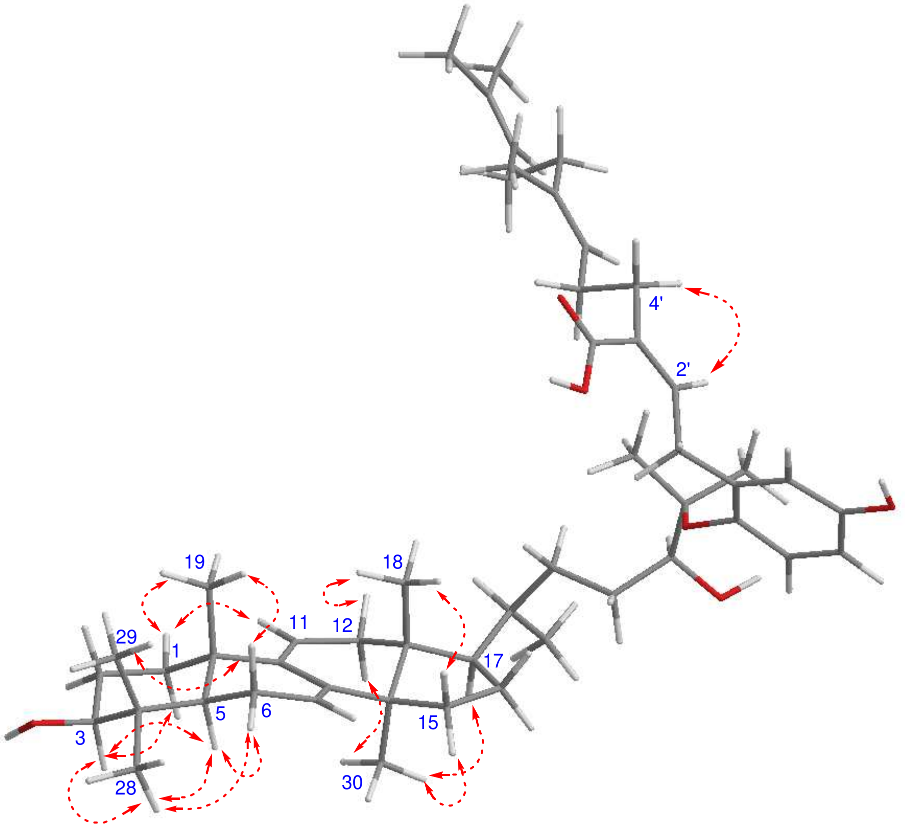

2

Key NOESY correlations for the lanostane unit

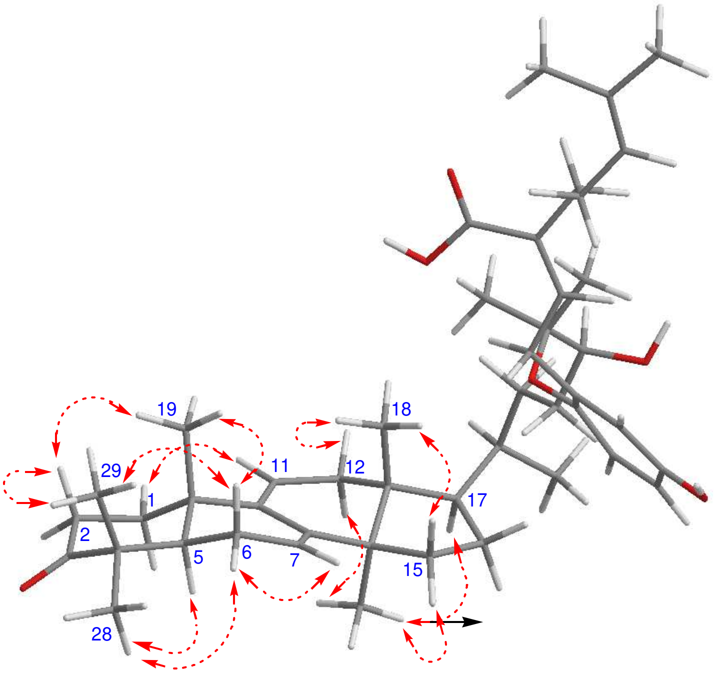

5

Key NOESY correlations for the lanostane unit

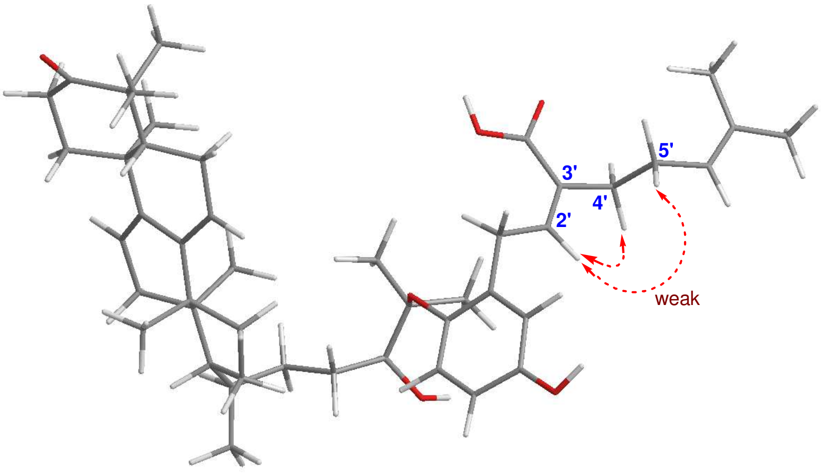

5

Key NOESY correlations for the meroterpene unit

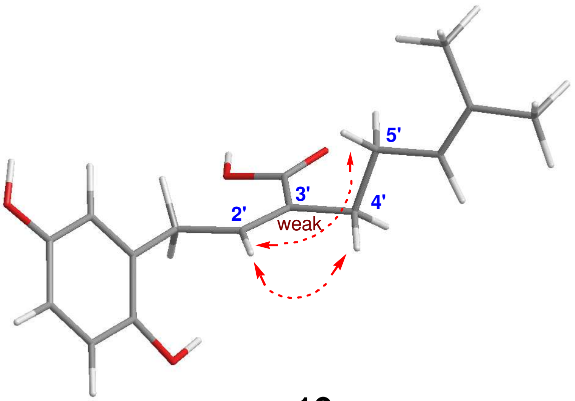

10

Figure S5. ^{1}H NMR spectrum of ganohochimin A (**1**) ($CDCl_3$, 500 MHz)

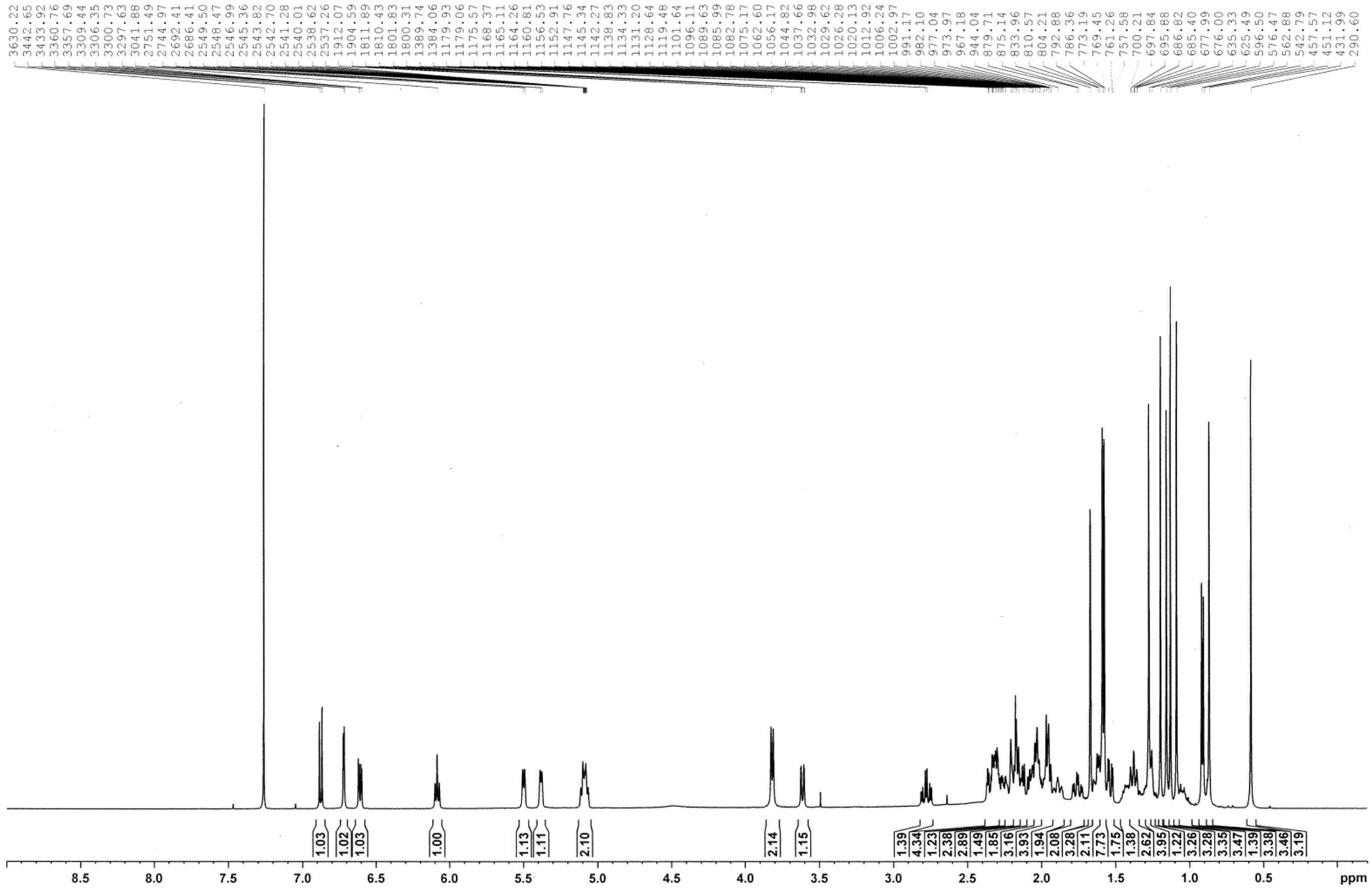

Figure S6. ^{13}C NMR spectrum of ganohochimin A (**1**) ($CDCl_3$, 125 MHz)

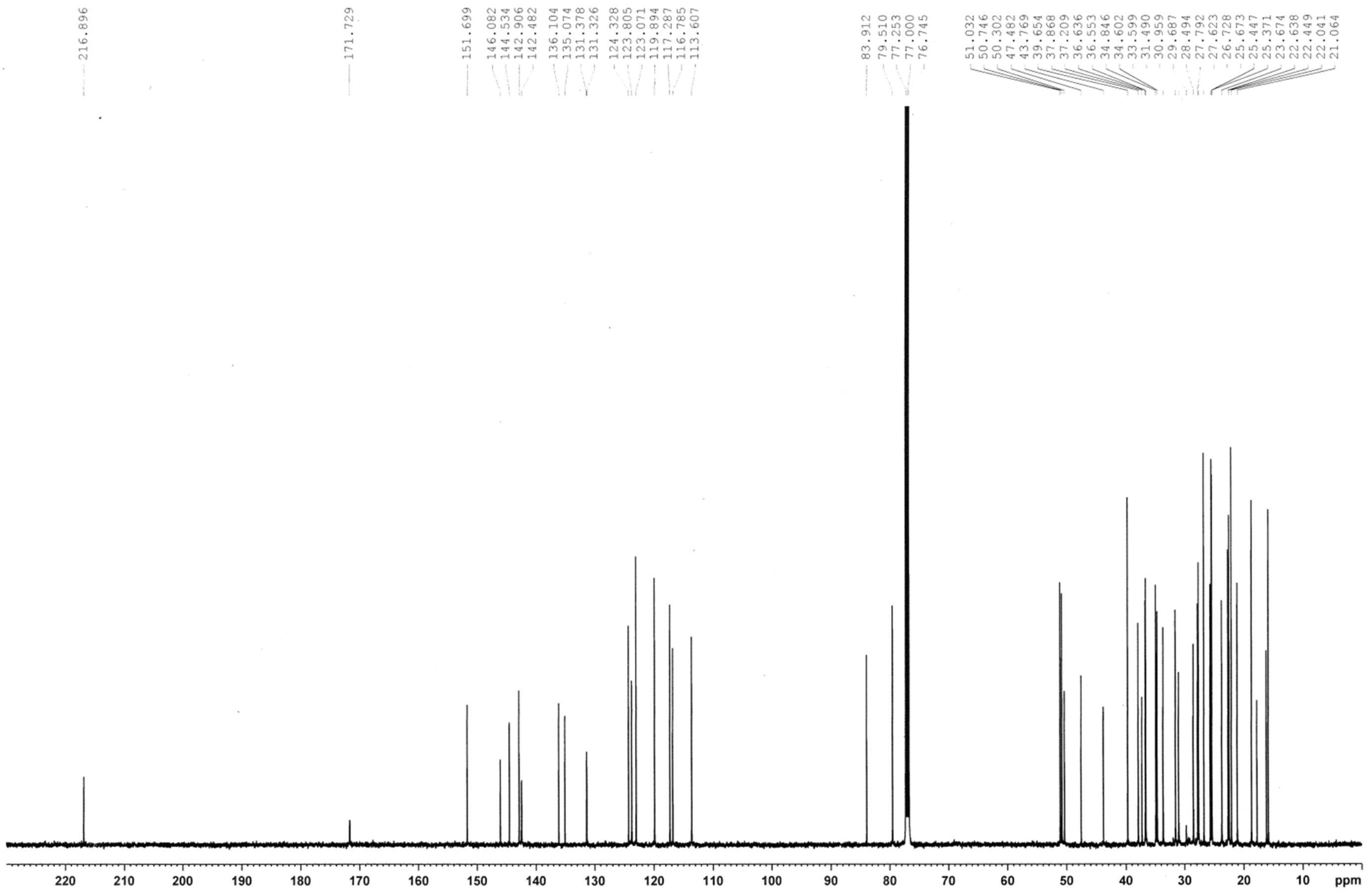

Figure S7. DEPT-135 spectrum of ganohochimin A (**1**) ($CDCl_3$, 125 MHz)

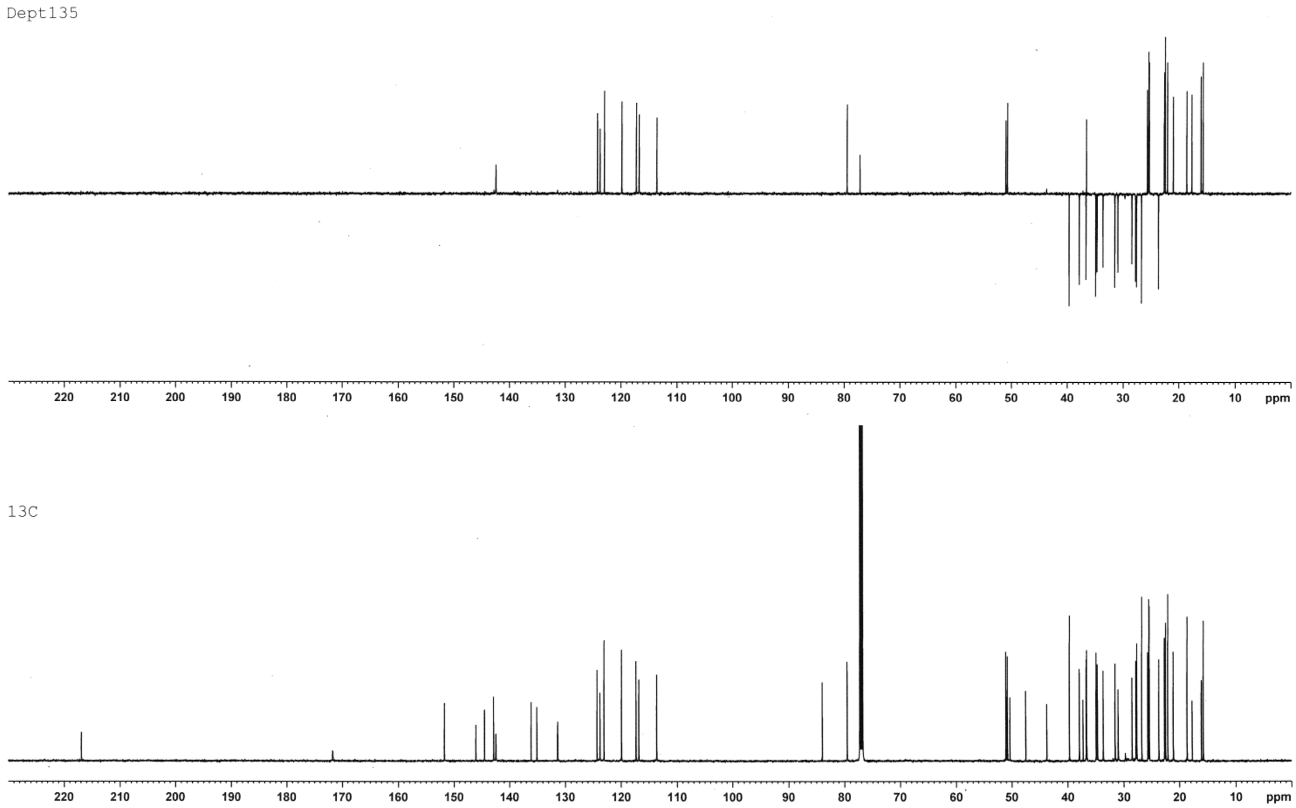

Figure S8. COSY spectrum of ganohochimin A (**1**) ($CDCl_3$, 500 MHz)

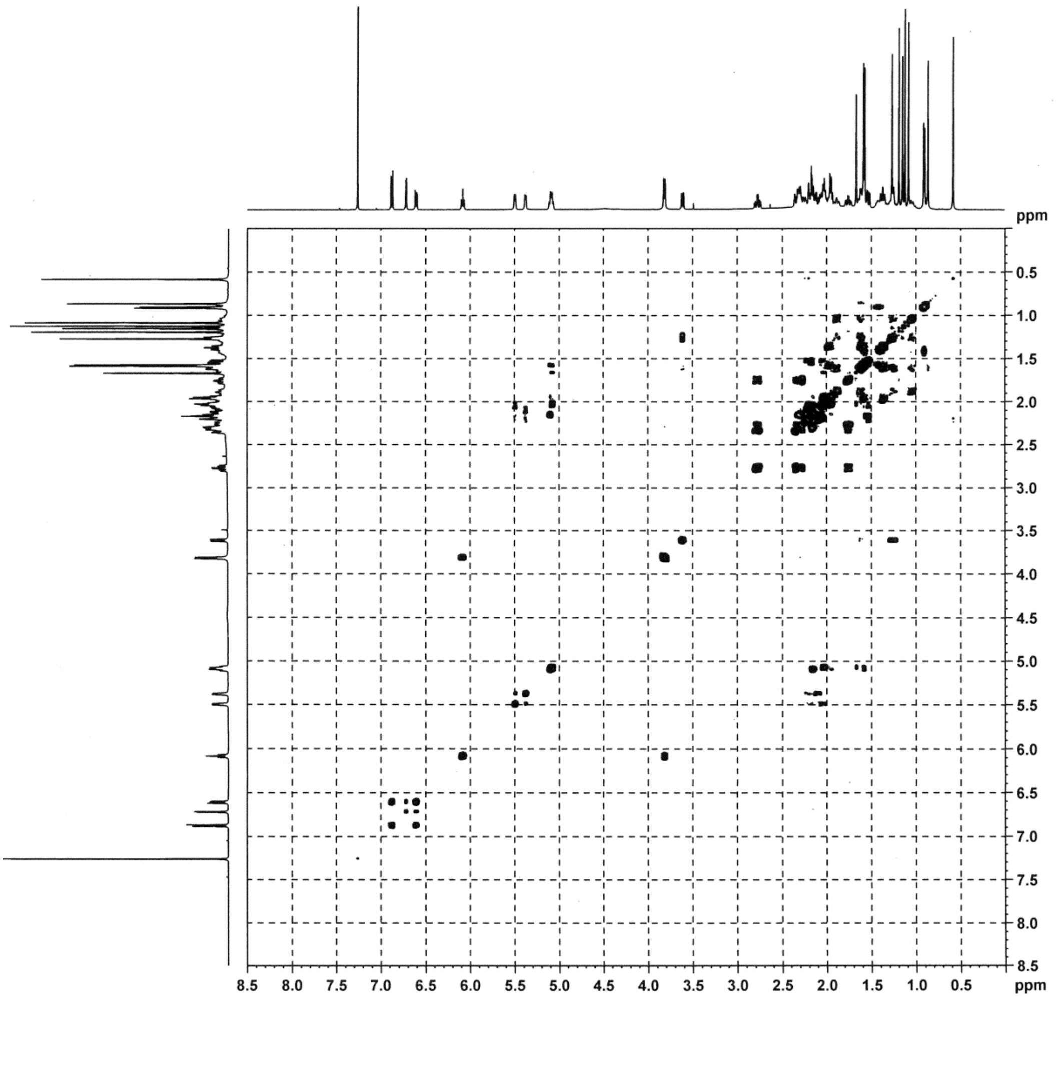

Figure S9. HSQC spectrum of ganohochimin A (**1**) ($CDCl_3$)

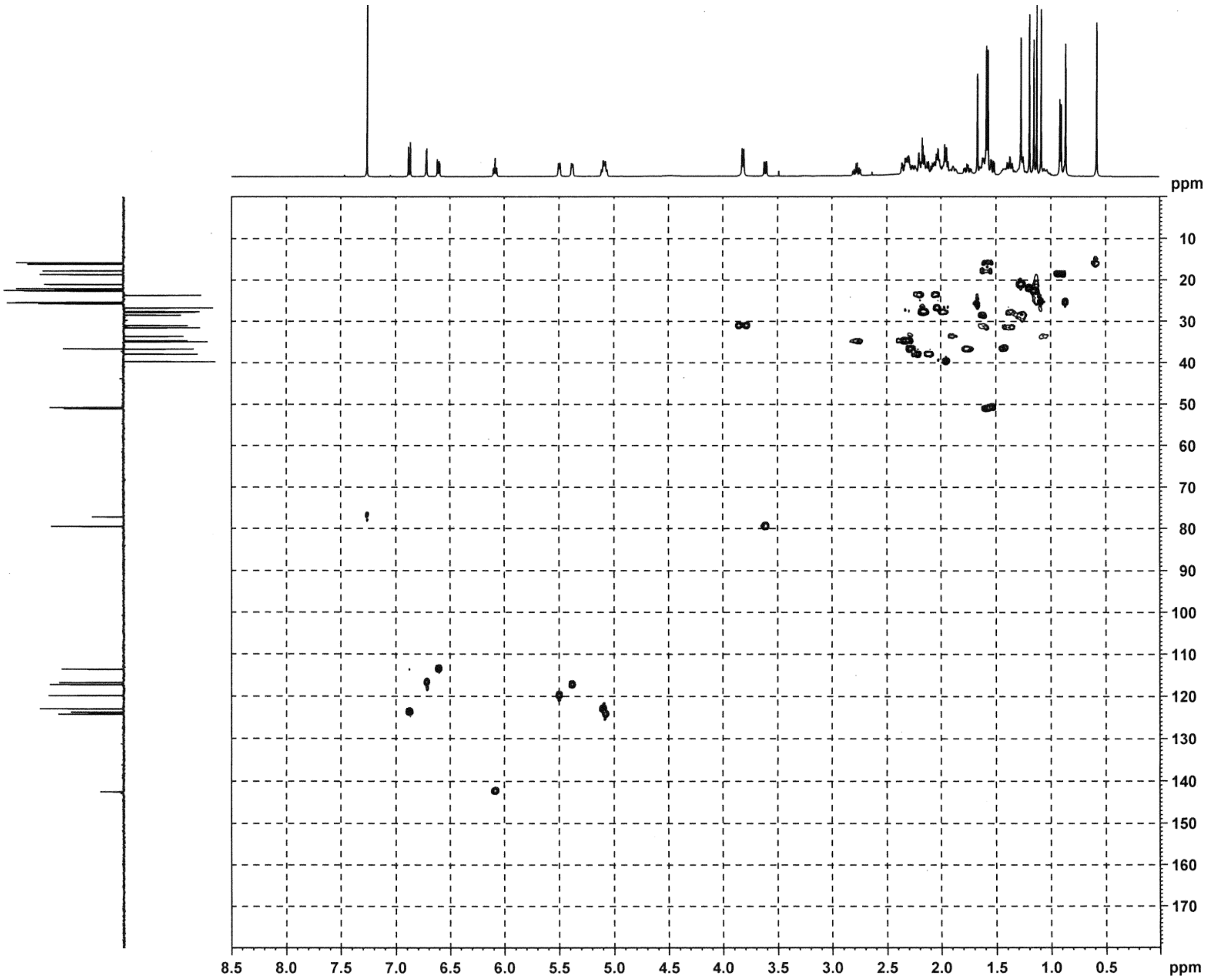

Figure S10. HMBC spectrum of ganohochimin A (**1**) ($CDCl_3$)

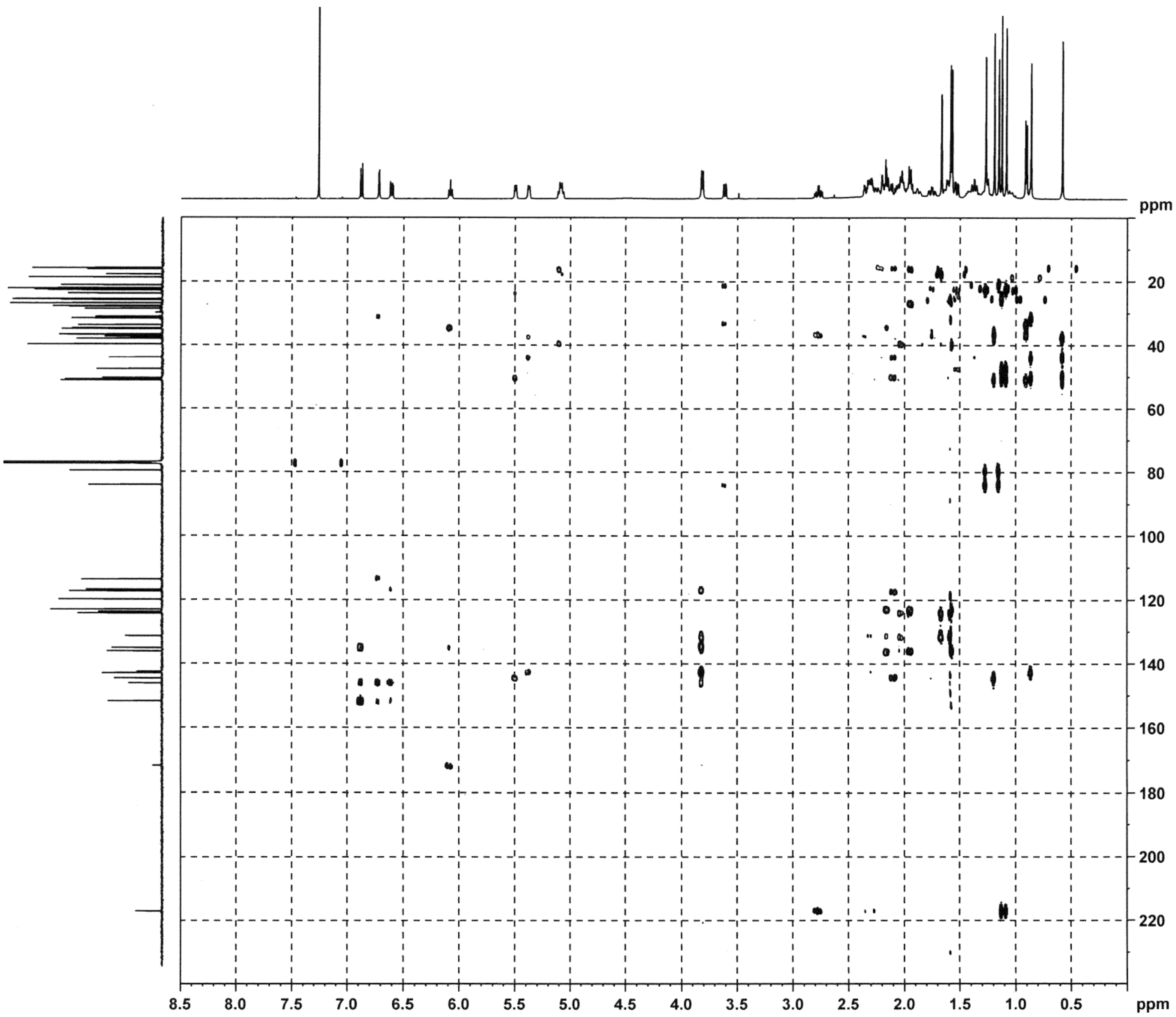

Figure S11. NOESY spectrum of ganohochimin A (**1**) ($CDCl_3$, 500 MHz)

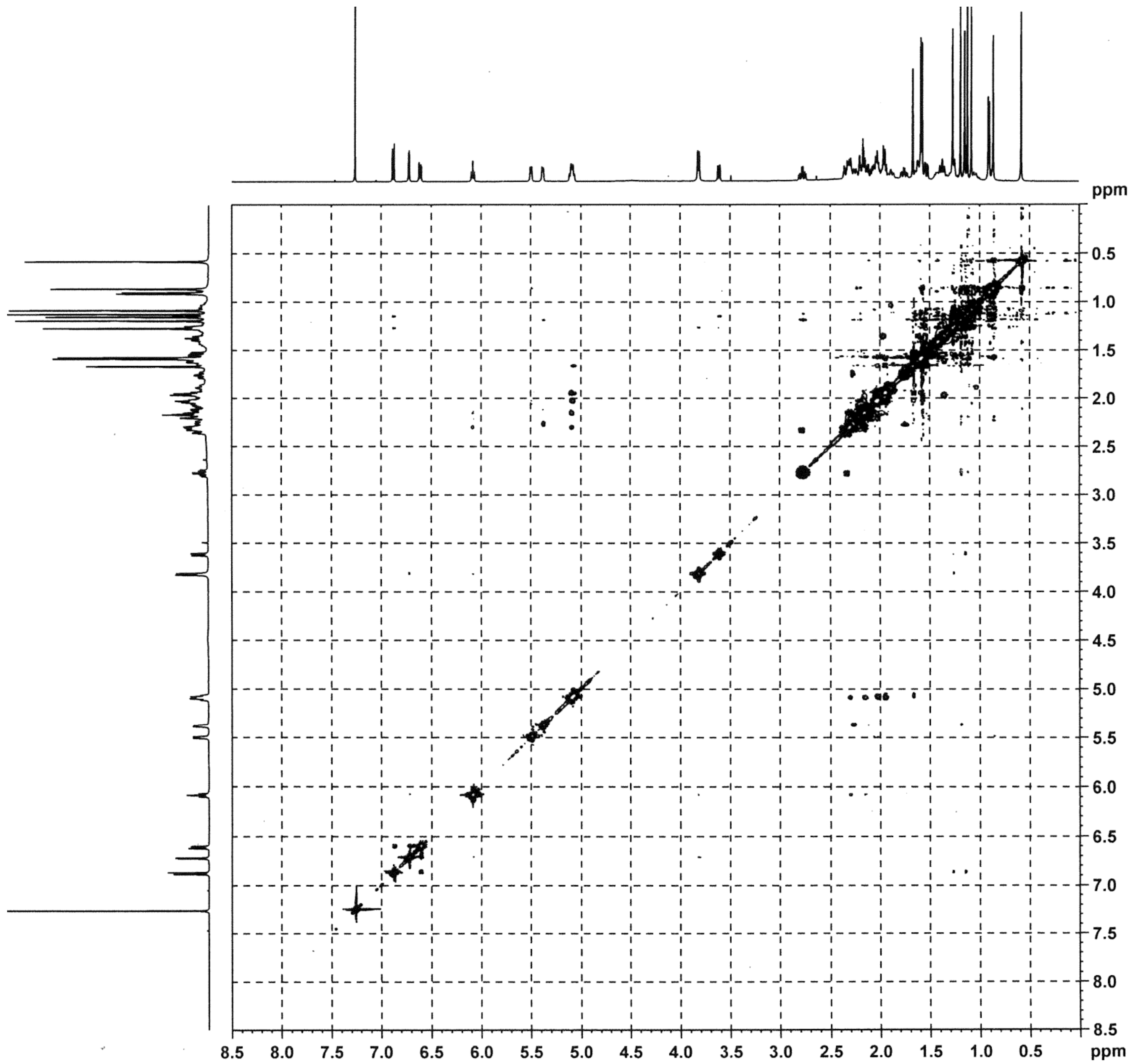

Figure S12. HRESIMS of ganohochimin A (**1**) (positive ion mode)

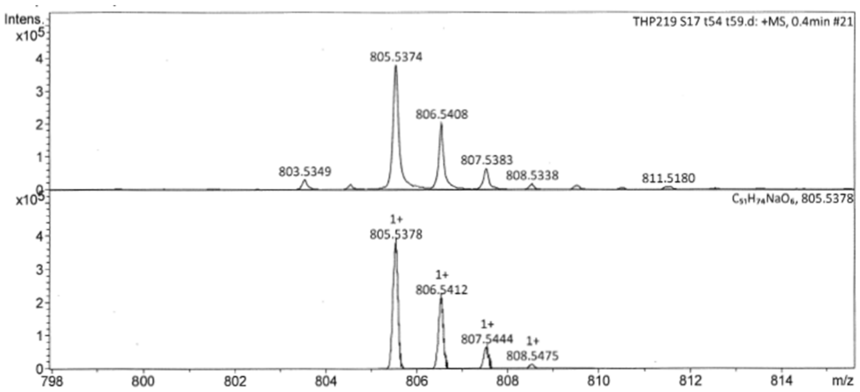

Figure S13. ^{1}H NMR spectrum of ganohochimin B (**2**) ($CDCl_3$, 500 MHz)

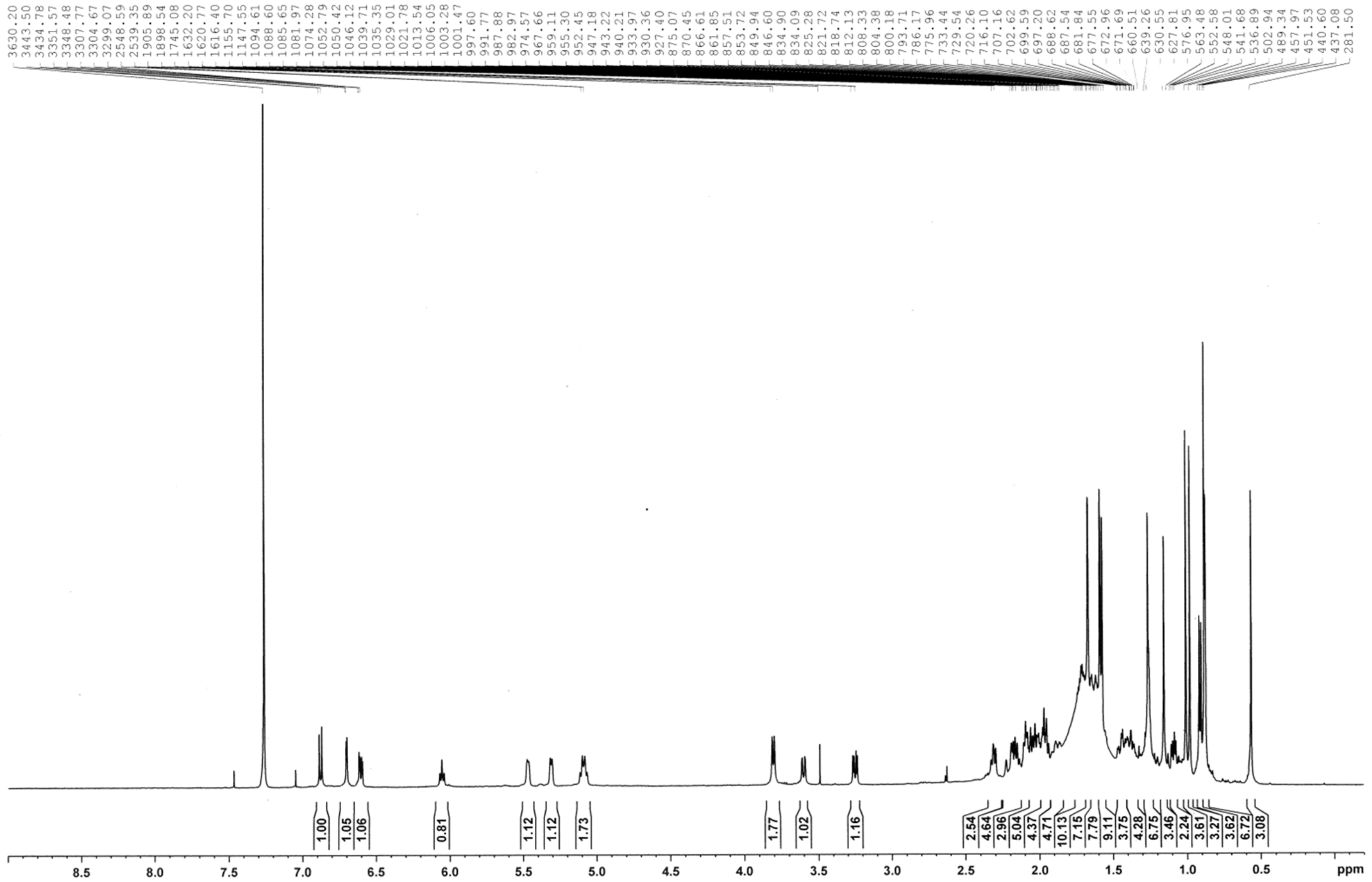

Figure S14. ^{13}C NMR spectrum of ganohochimin B (**2**) ($CDCl_3$, 125 MHz)

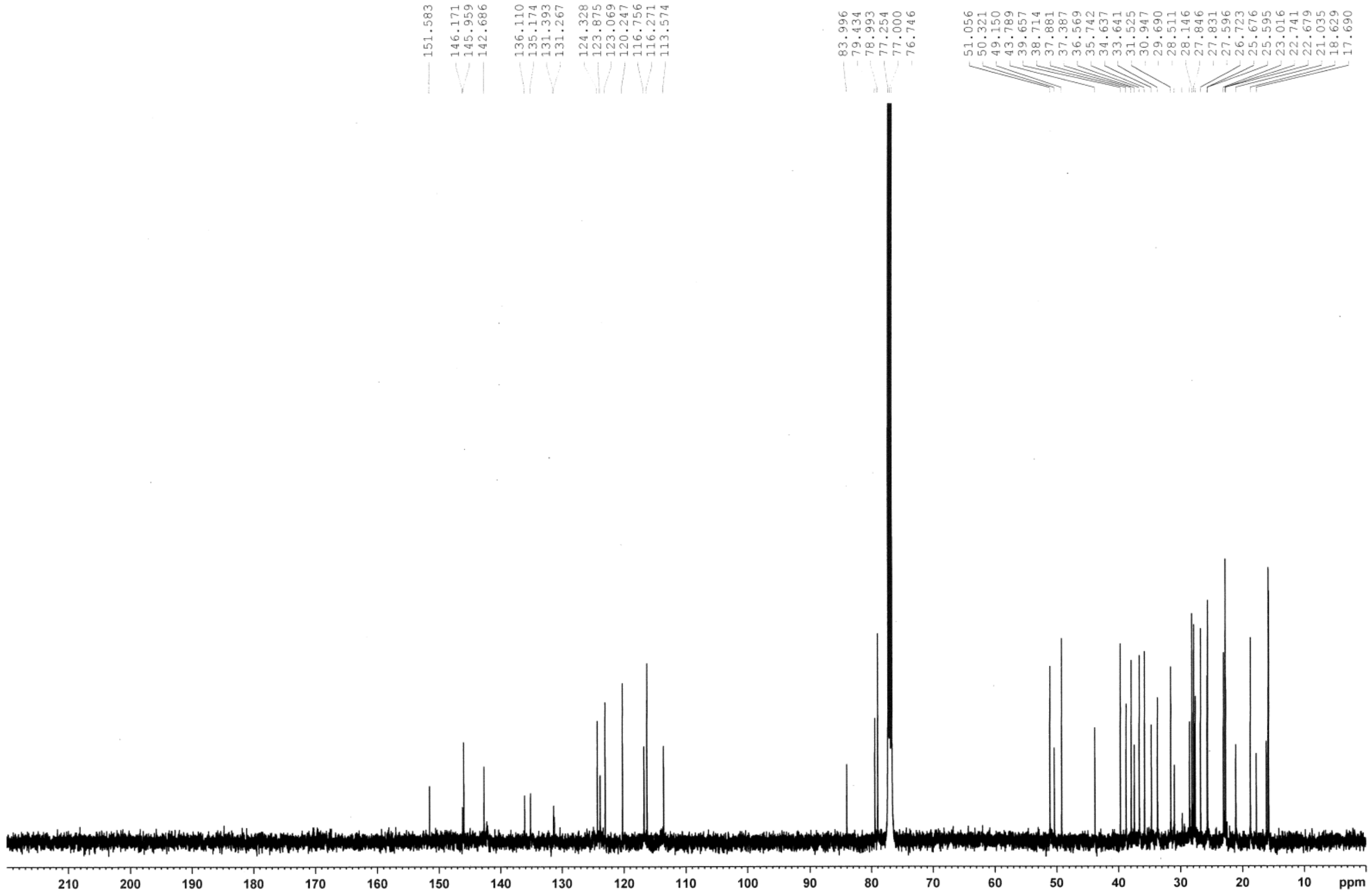

Figure S15. DEPT-135 spectrum of ganohochimin B (**2**) ($CDCl_3$, 125 MHz)

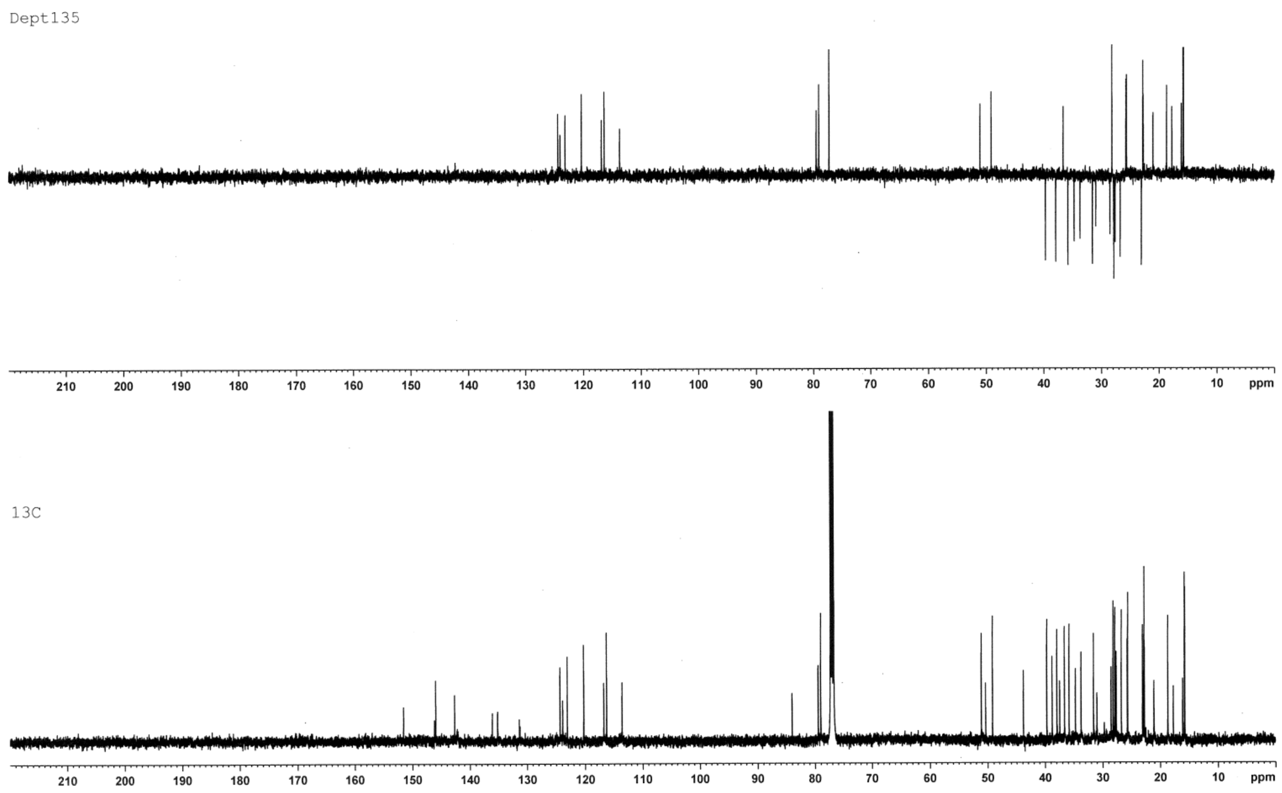

Figure S16. COSY spectrum of ganohochimin B (**2**) ($CDCl_3$, 500 MHz)

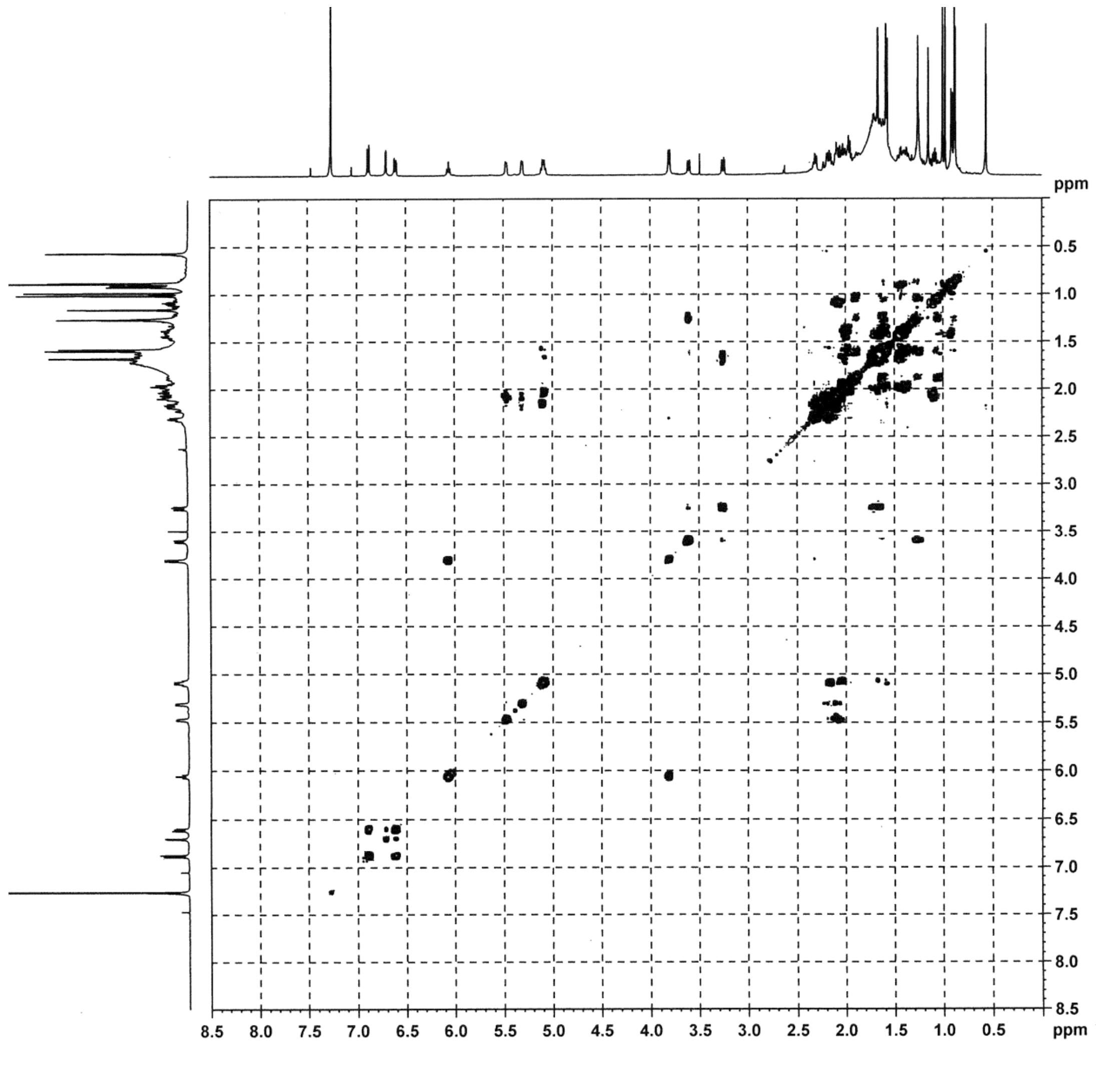

Figure S17. HSQC spectrum of ganohochimin B (**2**) ($CDCl_3$)

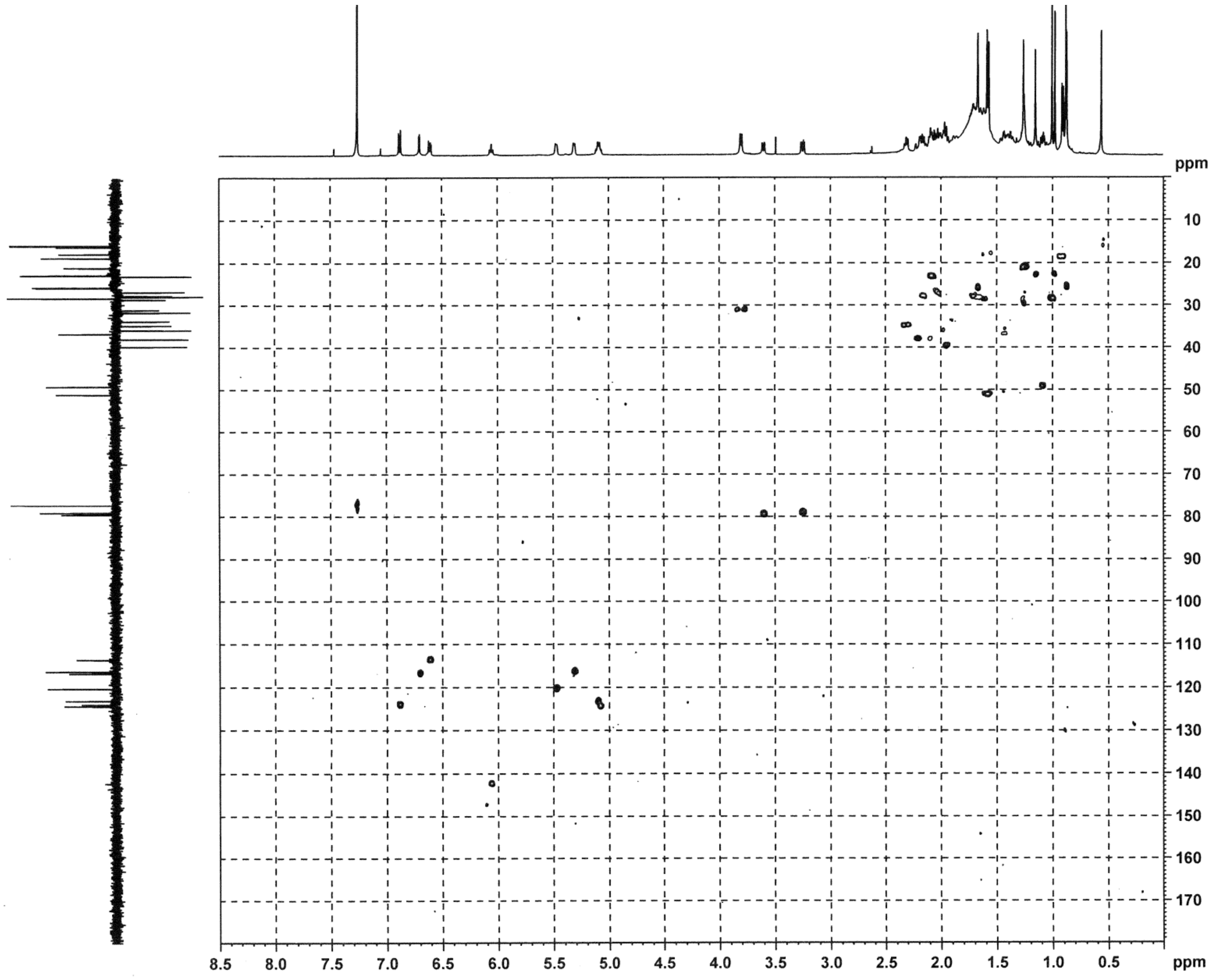

Figure S18. HMBC spectrum of ganohochimin B (**2**) (CDCl$_3$)

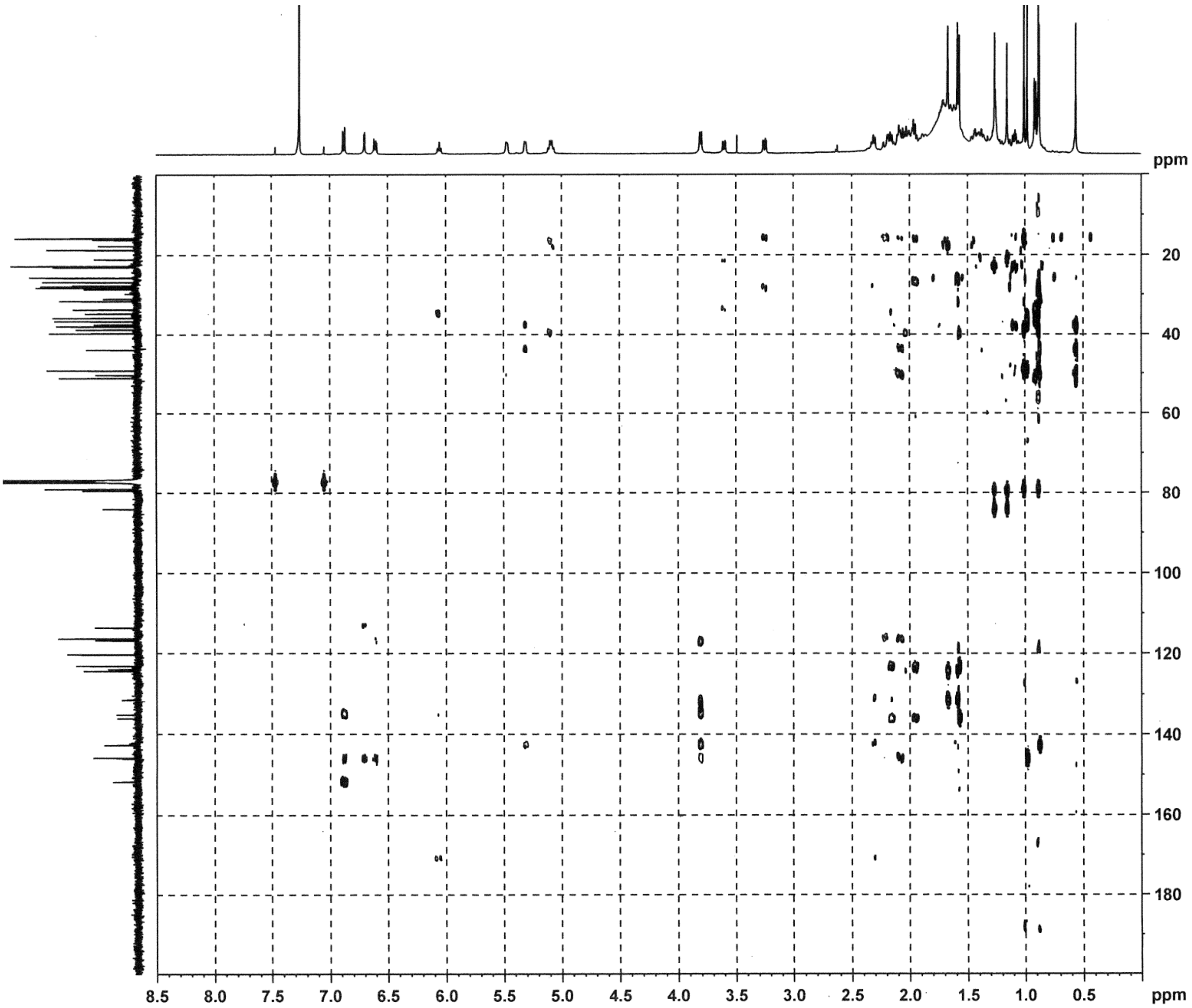

Figure S19. NOESY spectrum of ganohochimin B (**2**) ($CDCl_3$, 500 MHz)

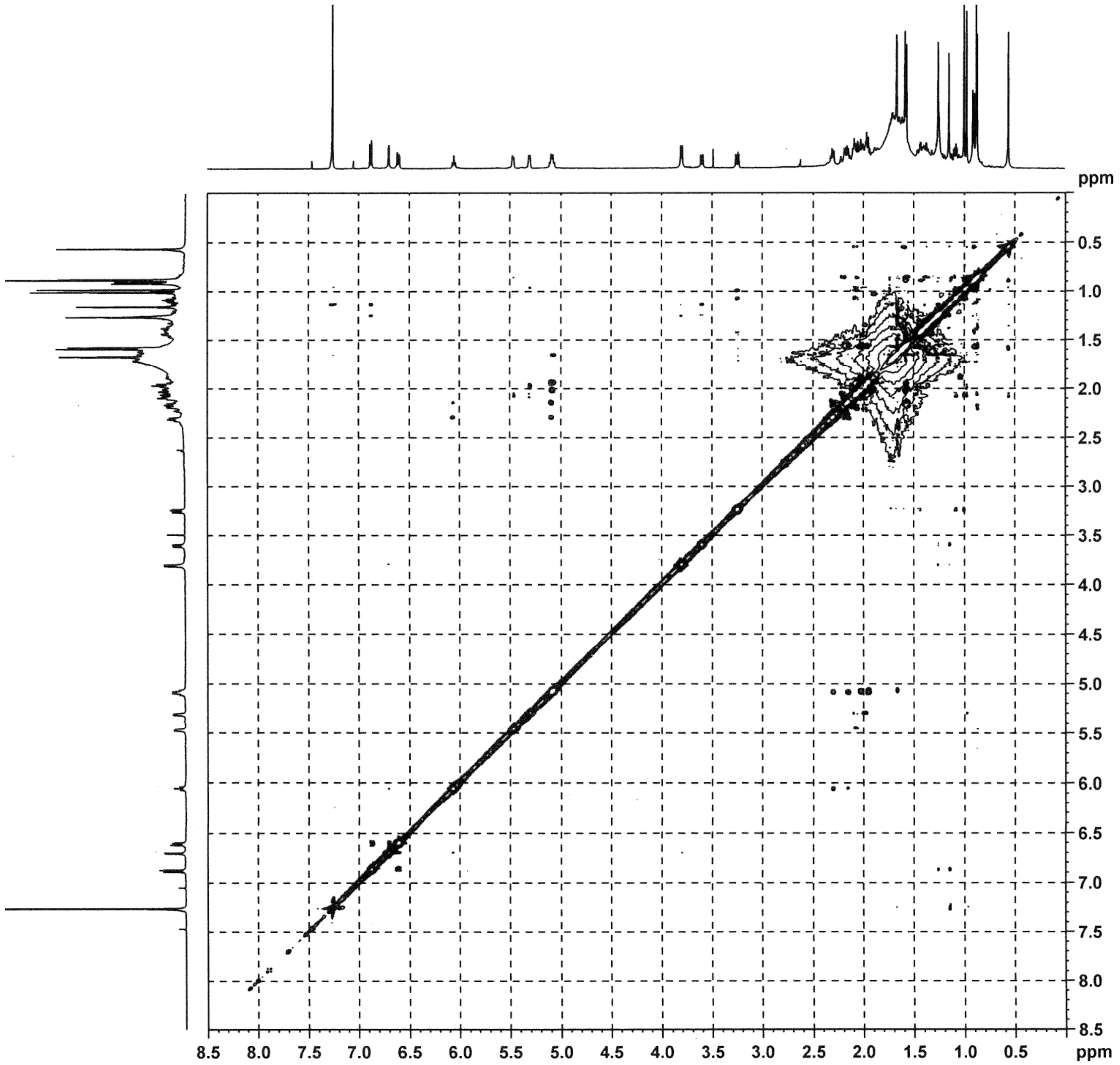

Figure S20. HRESIMS of ganohochimin B (**2**) (positive ion mode)

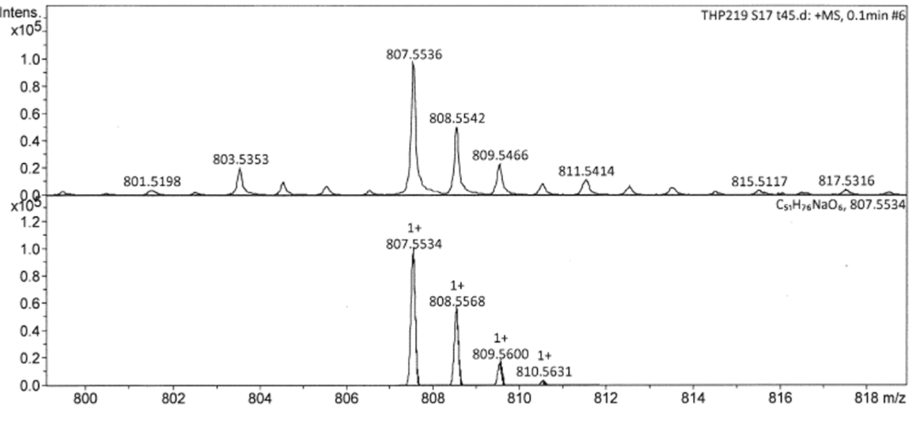

Figure S21. ^{1}H NMR spectrum of ganohochimin C (**3**) ($CDCl_3$, 500 MHz)

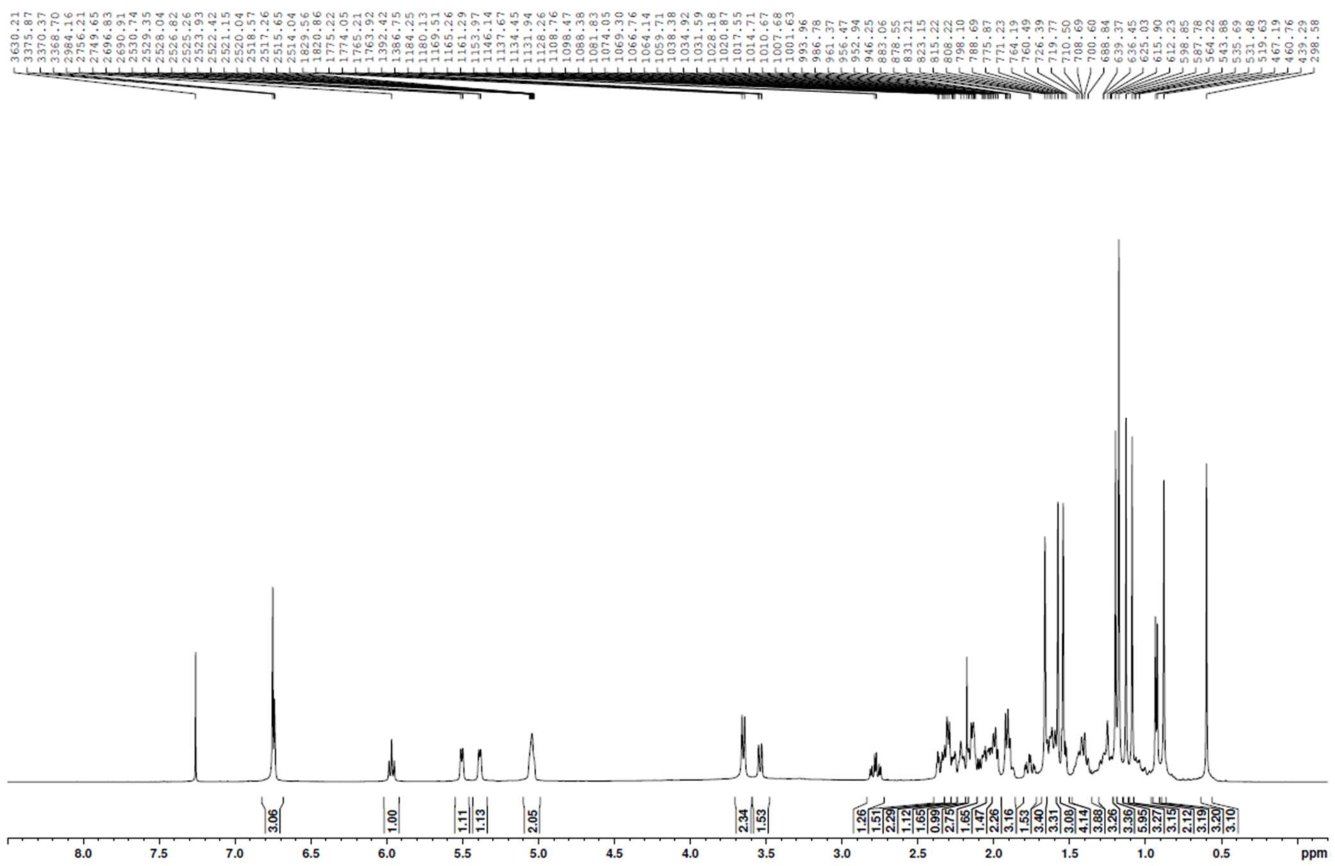

Figure S22. ^{13}C NMR spectrum of ganohochimin C (**3**) ($CDCl_3$, 125 MHz)

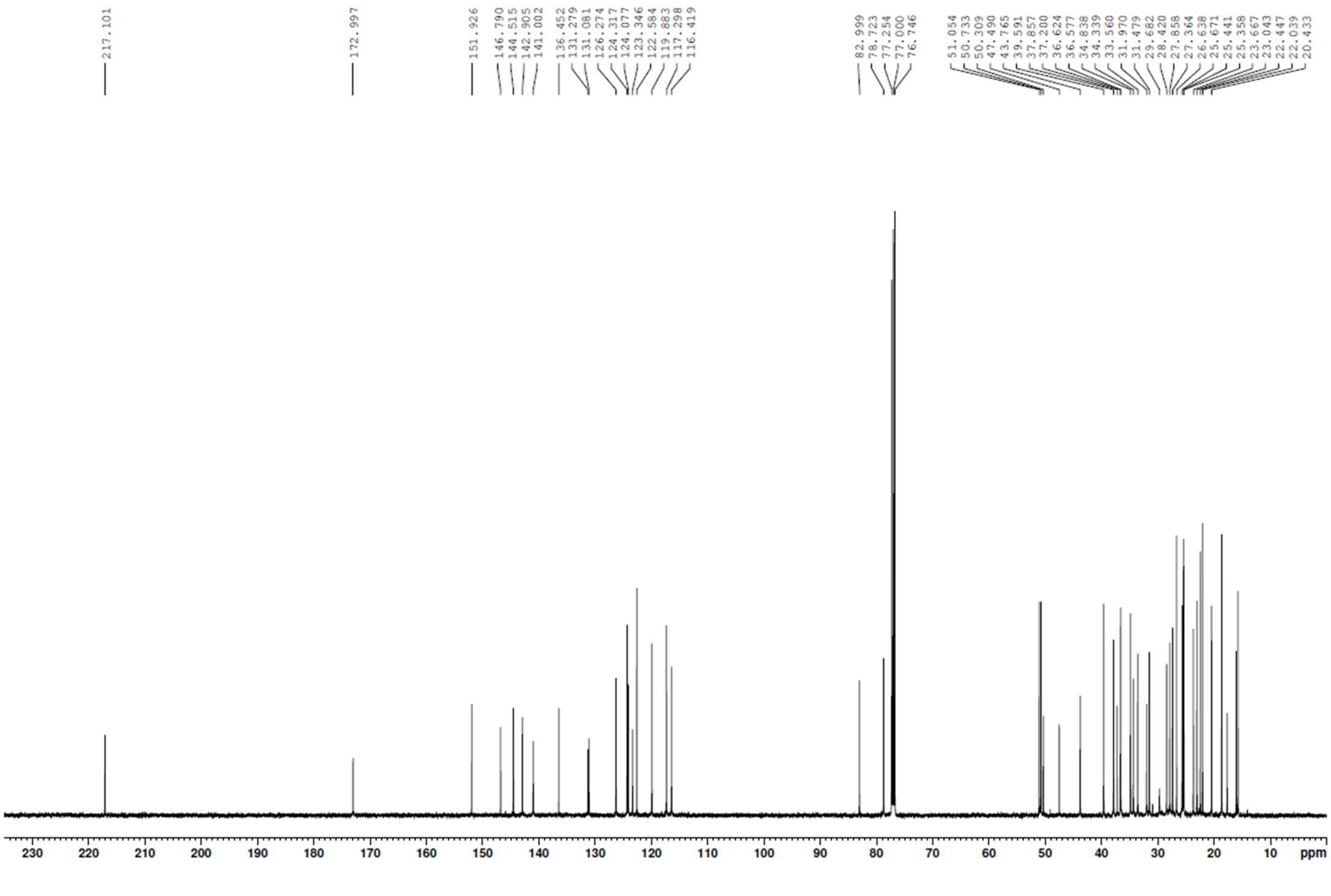

Figure S23. DEPT-135 spectrum of ganohochimin C (**3**) ($CDCl_3$, 125 MHz)

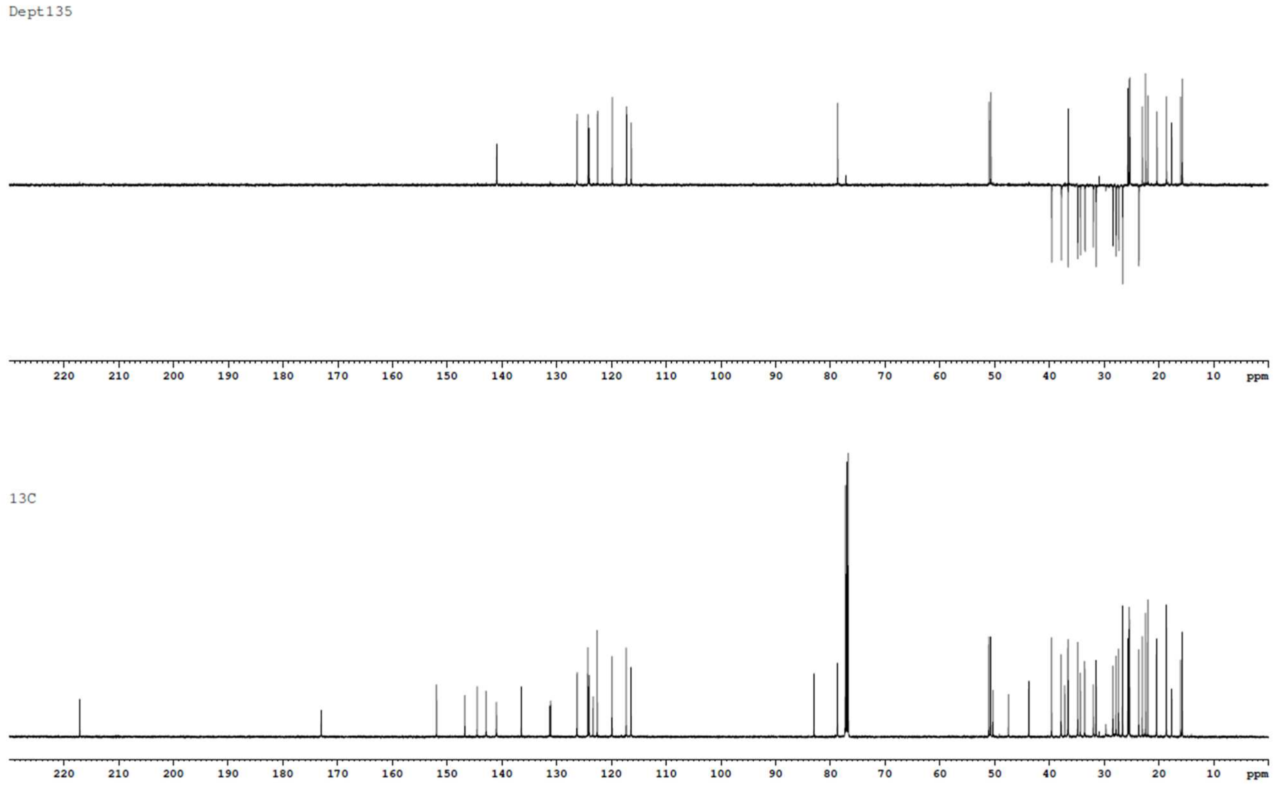

Figure S24. COSY spectrum of ganohochimin C (**3**) ($CDCl_3$, 500 MHz)

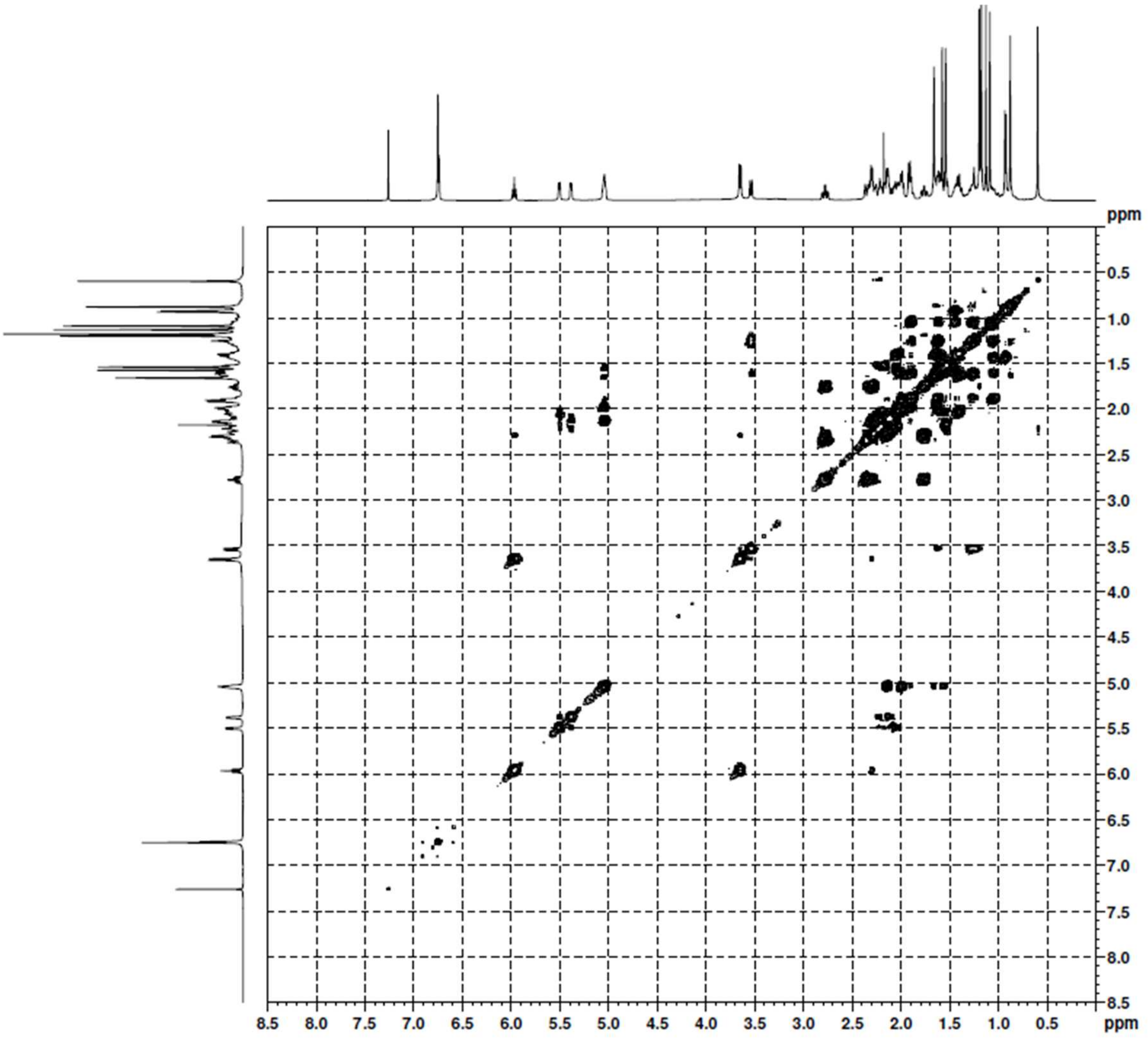

Figure S25. HSQC spectrum of ganohochimin C (**3**) ($CDCl_3$)

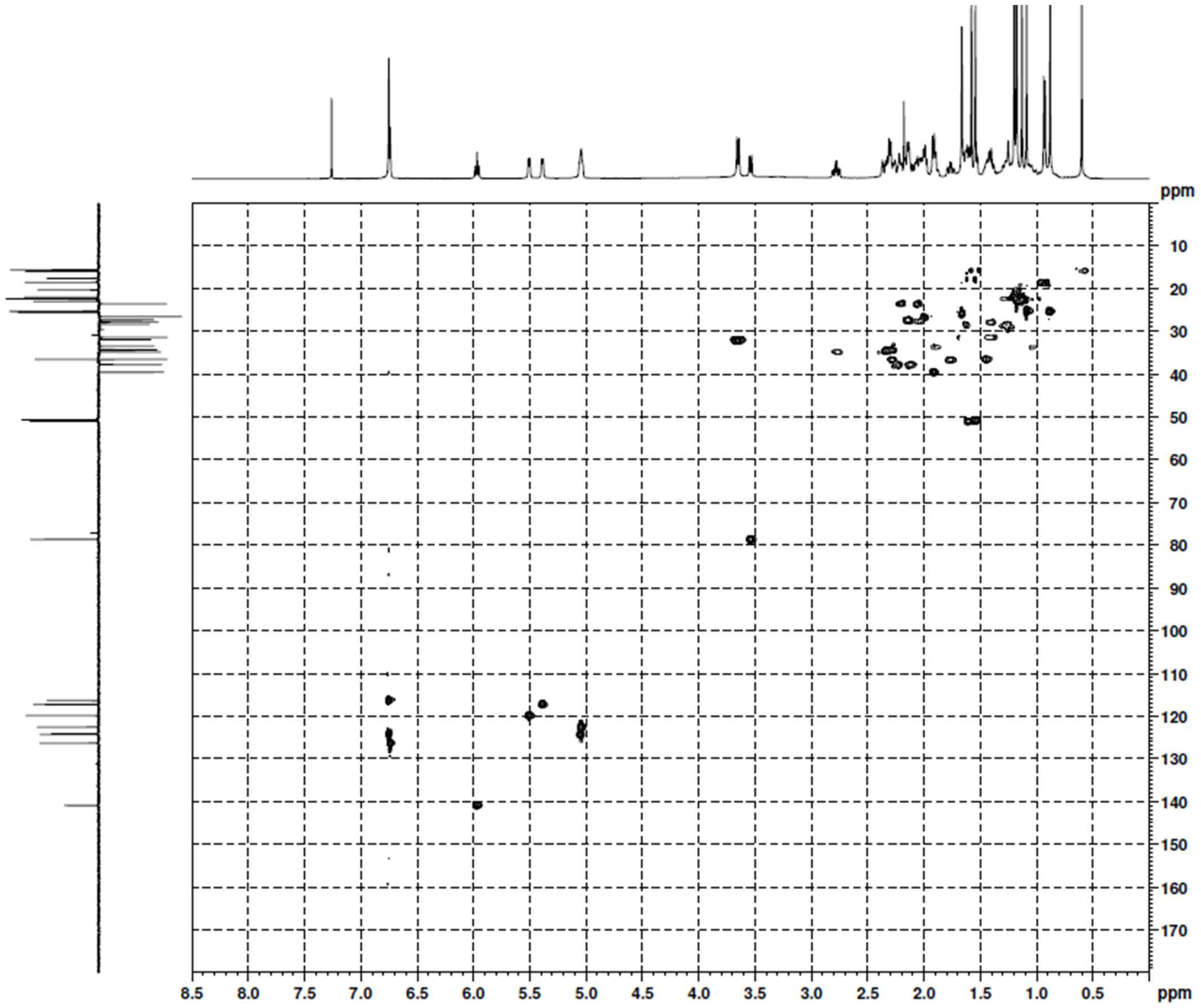

Figure S26. HMBC spectrum of ganohochimin C (**3**) ($CDCl_3$)

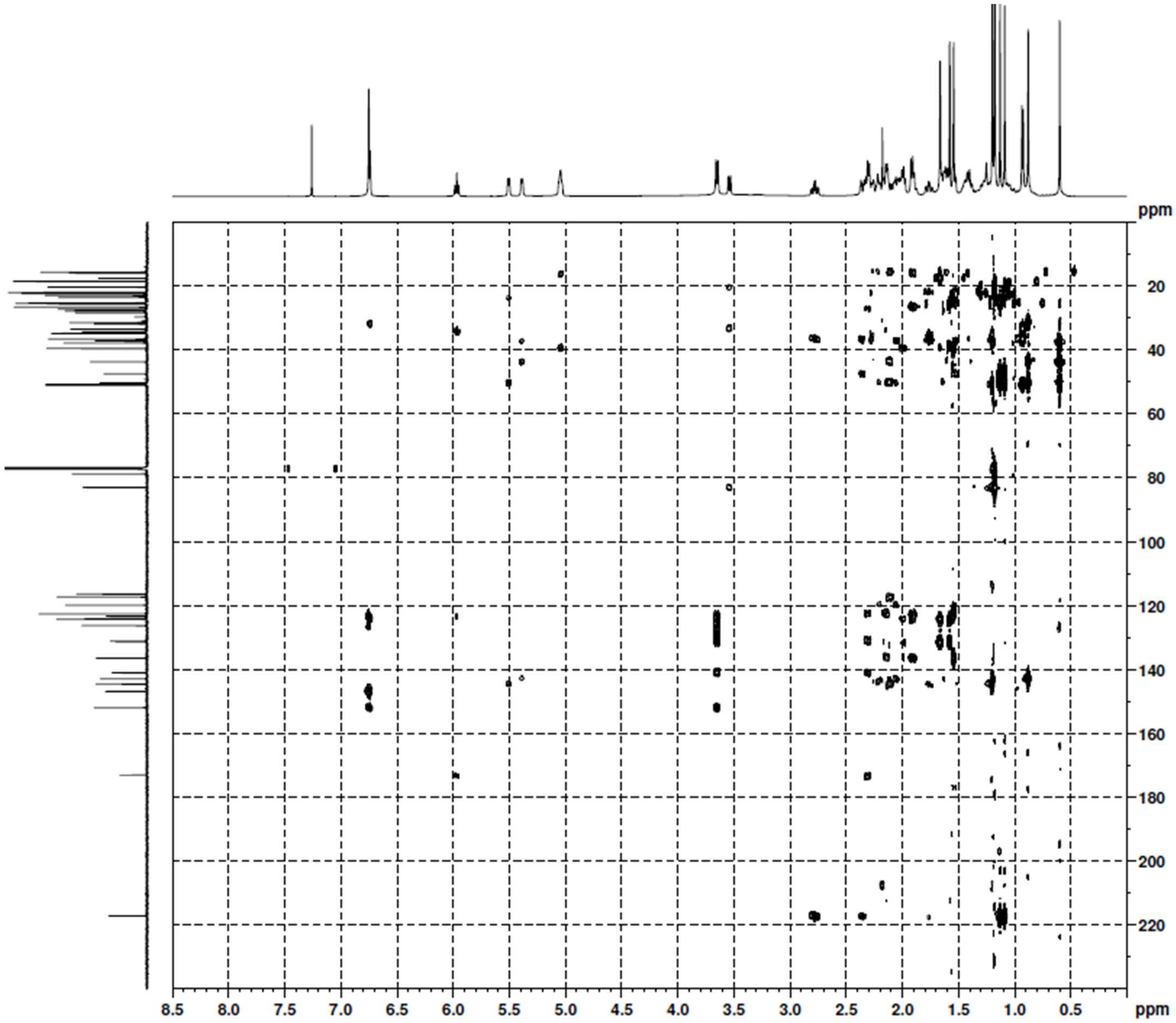

Figure S27. NOESY spectrum of ganohochimin C (**3**) ($CDCl_3$, 500 MHz)

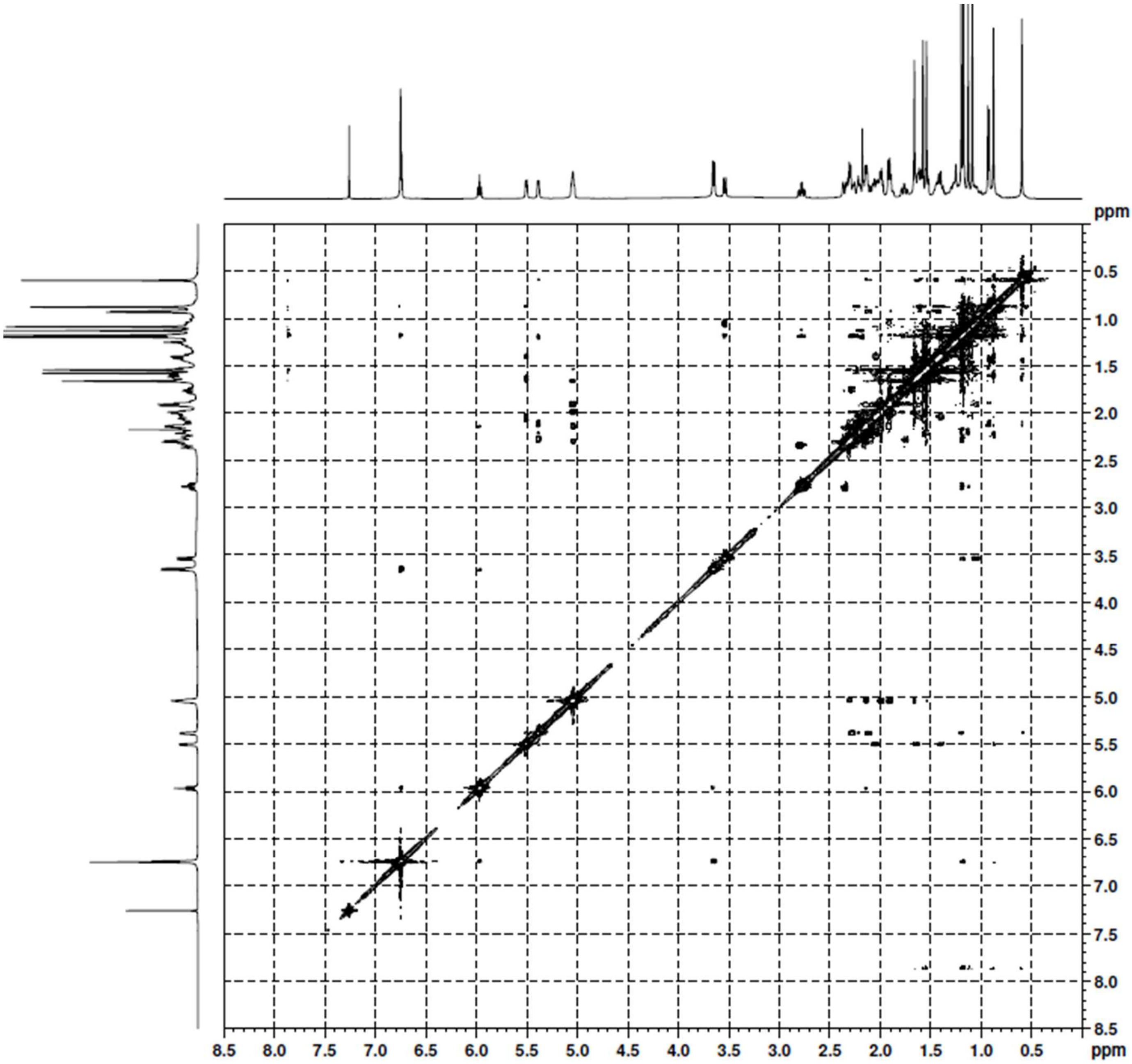

Figure S28. HRESIMS of ganohochimin C (**3**) (positive ion mode)

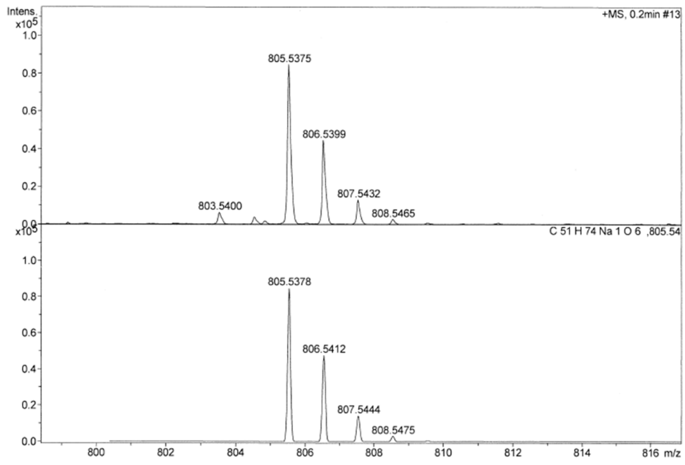

Figure S29. ^{1}H NMR spectrum of ganohochimin D (**4**) ($CDCl_3$, 500 MHz)

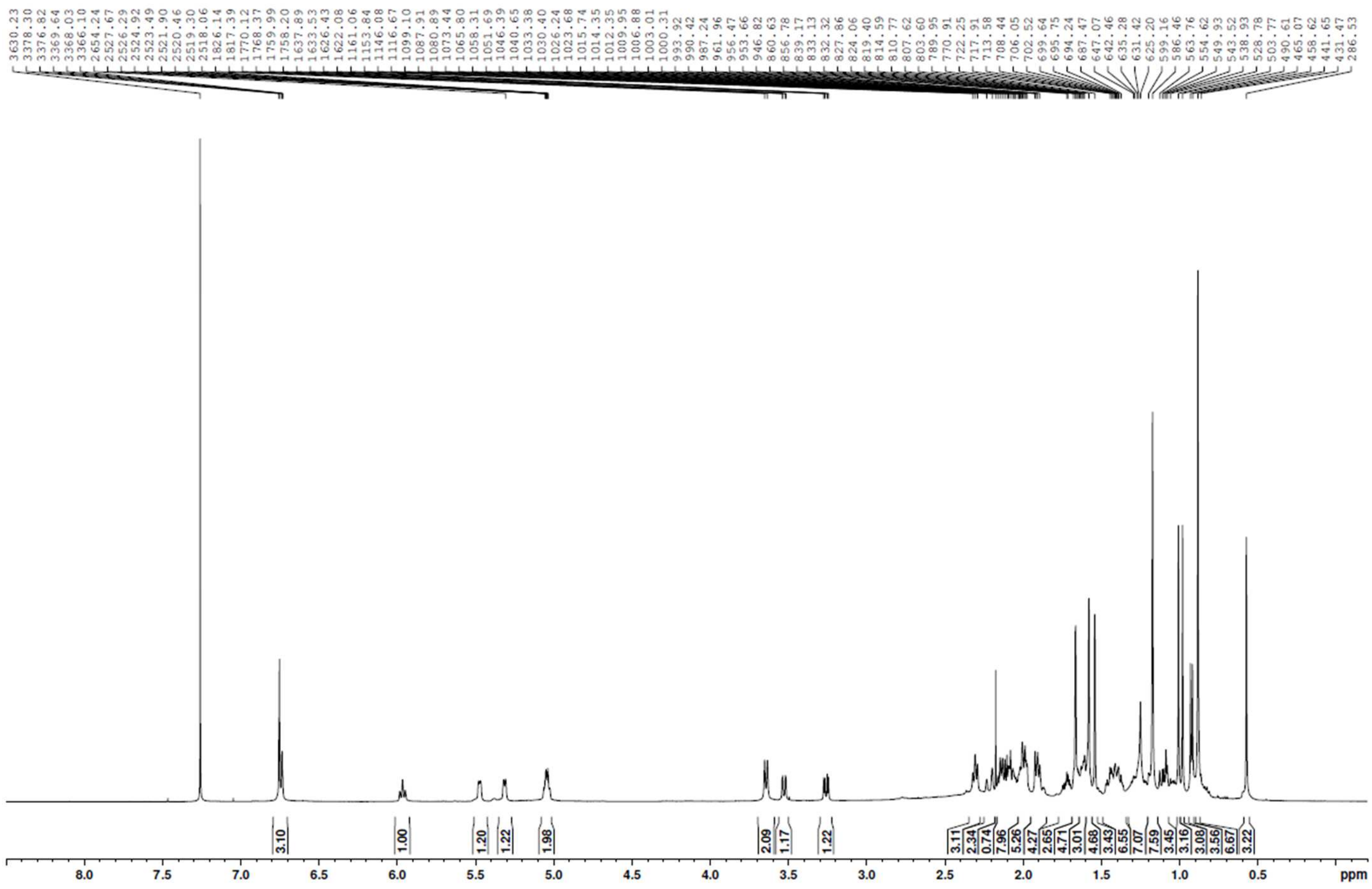

Figure S30. ^{13}C NMR spectrum of ganohochimin D (**4**) ($CDCl_3$, 125 MHz)

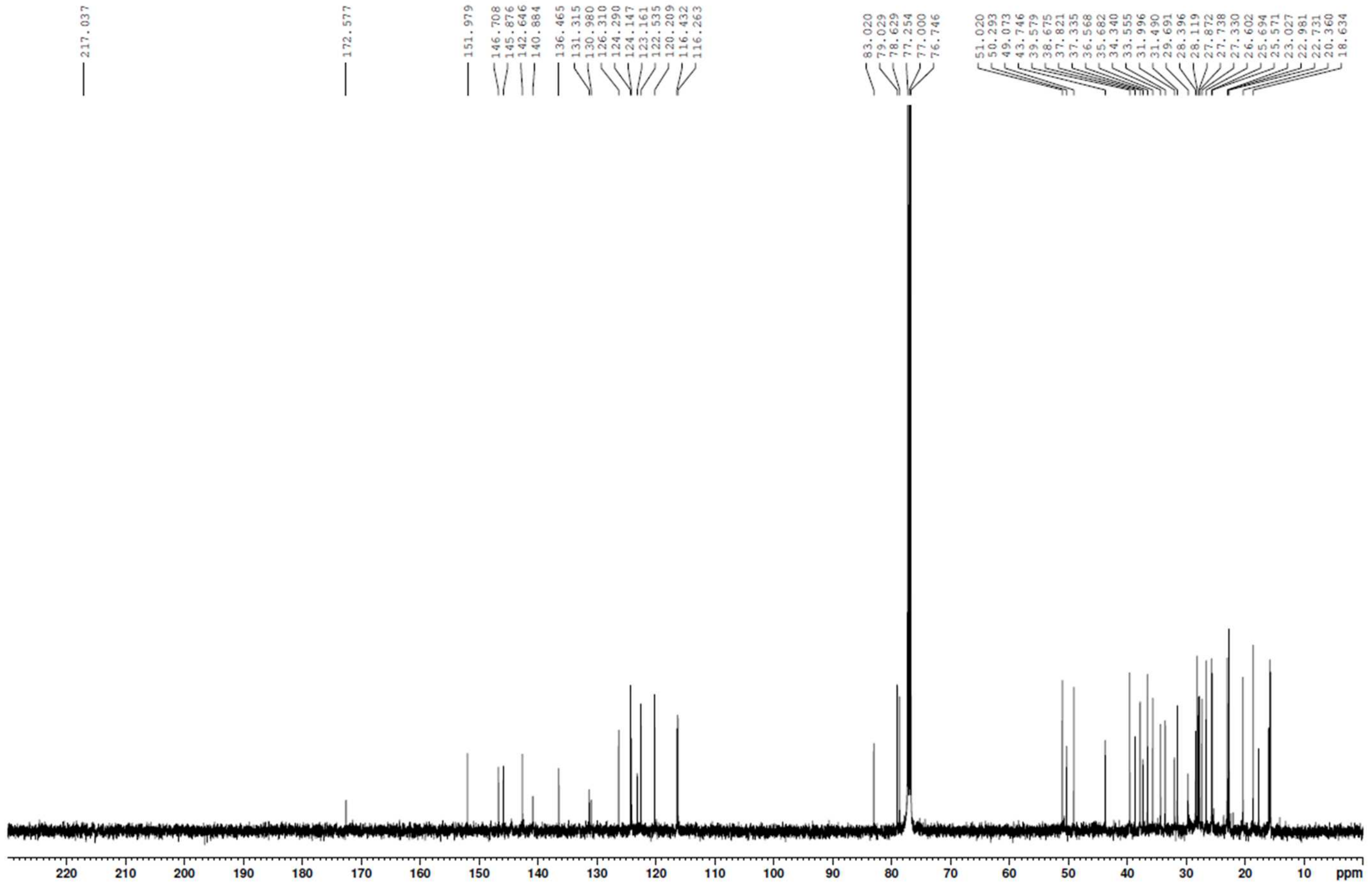

Figure S31. DEPT-135 spectrum of ganohochimin D (**4**) ($CDCl_3$, 125 MHz)

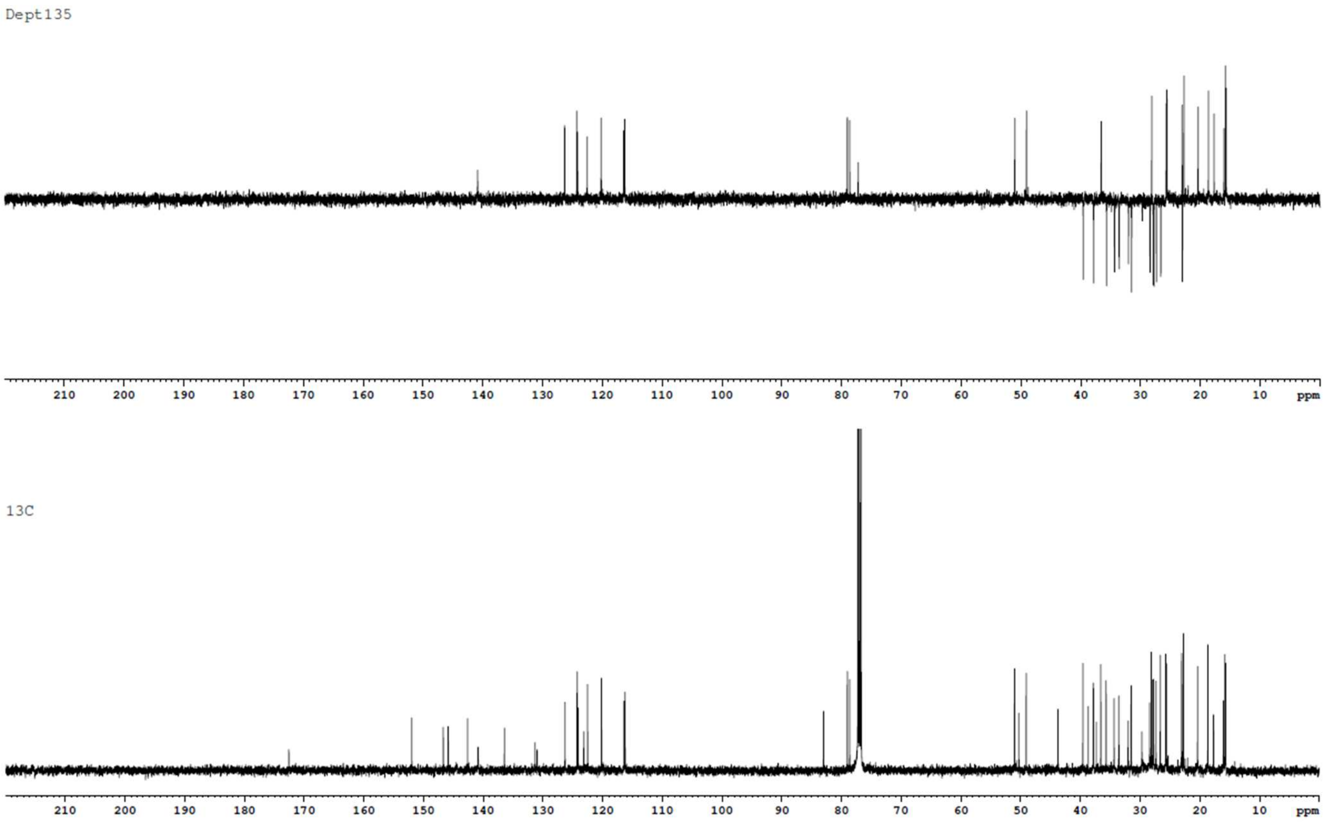

Figure S32. COSY spectrum of ganohochimin D (**4**) ($CDCl_3$, 500 MHz)

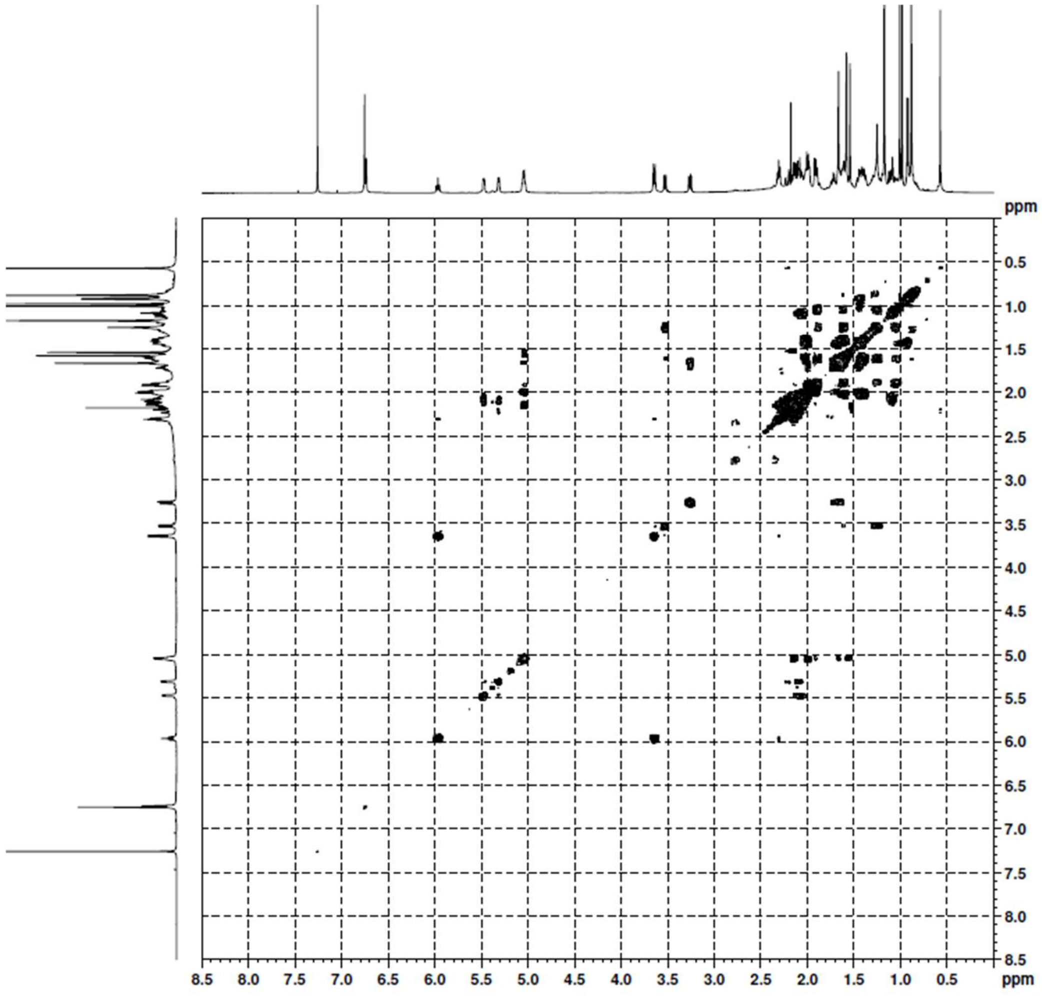

Figure S33. HSQC spectrum of ganohochimin D (**4**) ($CDCl_3$)

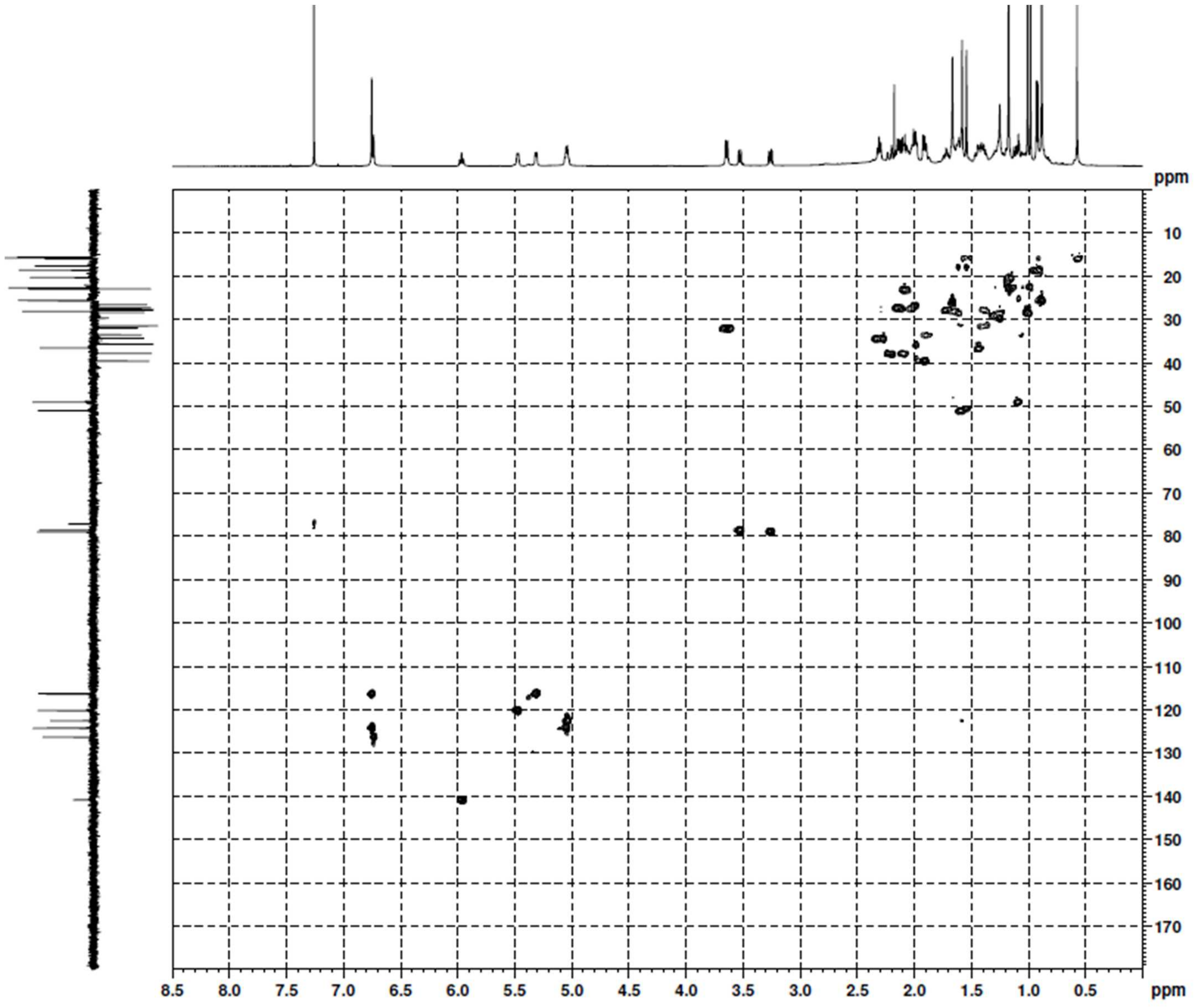

Figure S34. HMBC spectrum of ganohochimin D (**3**) ($CDCl_3$)

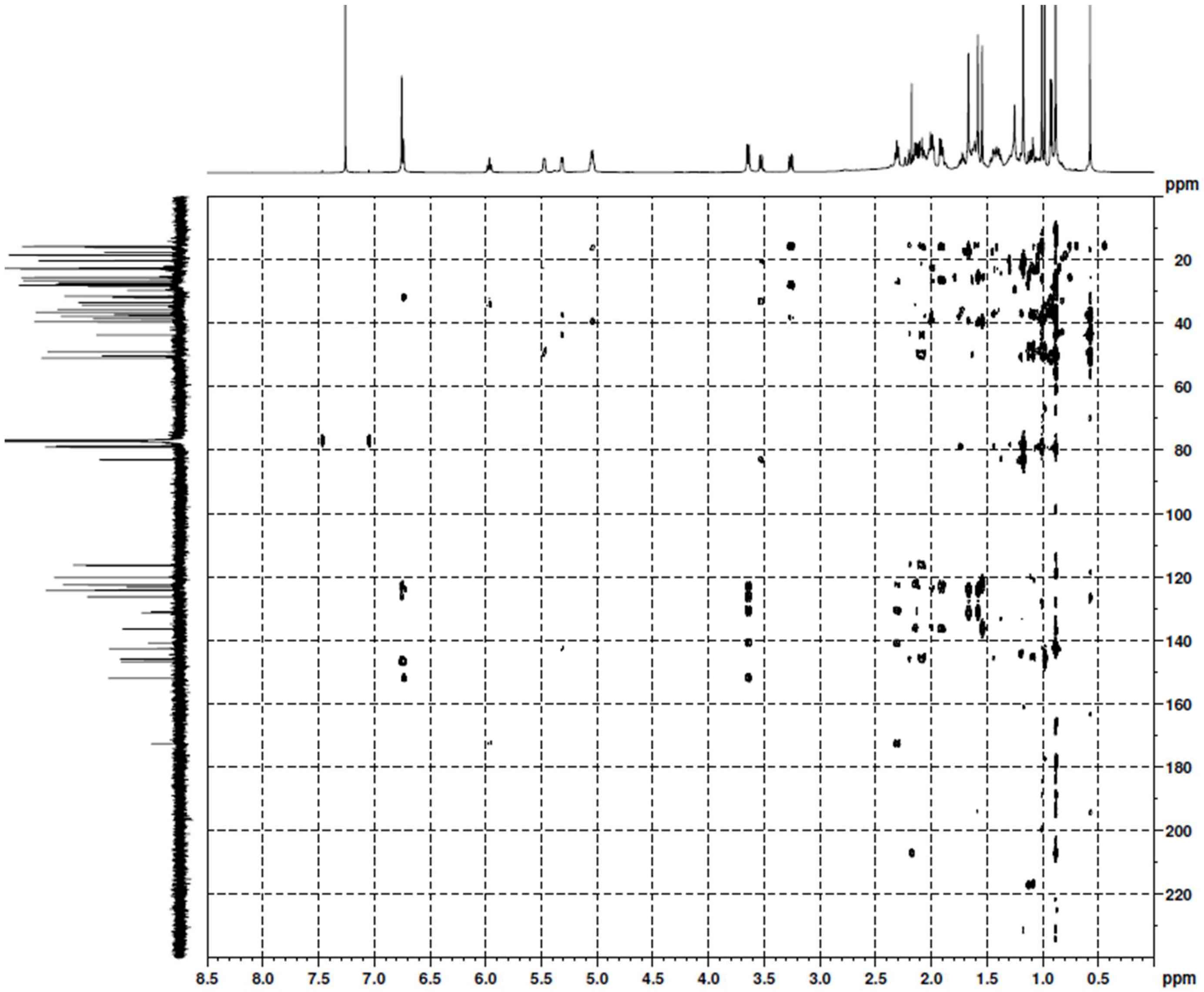

Figure S35. NOESY spectrum of ganohochimin C (**4**) ($CDCl_3$, 500 MHz)

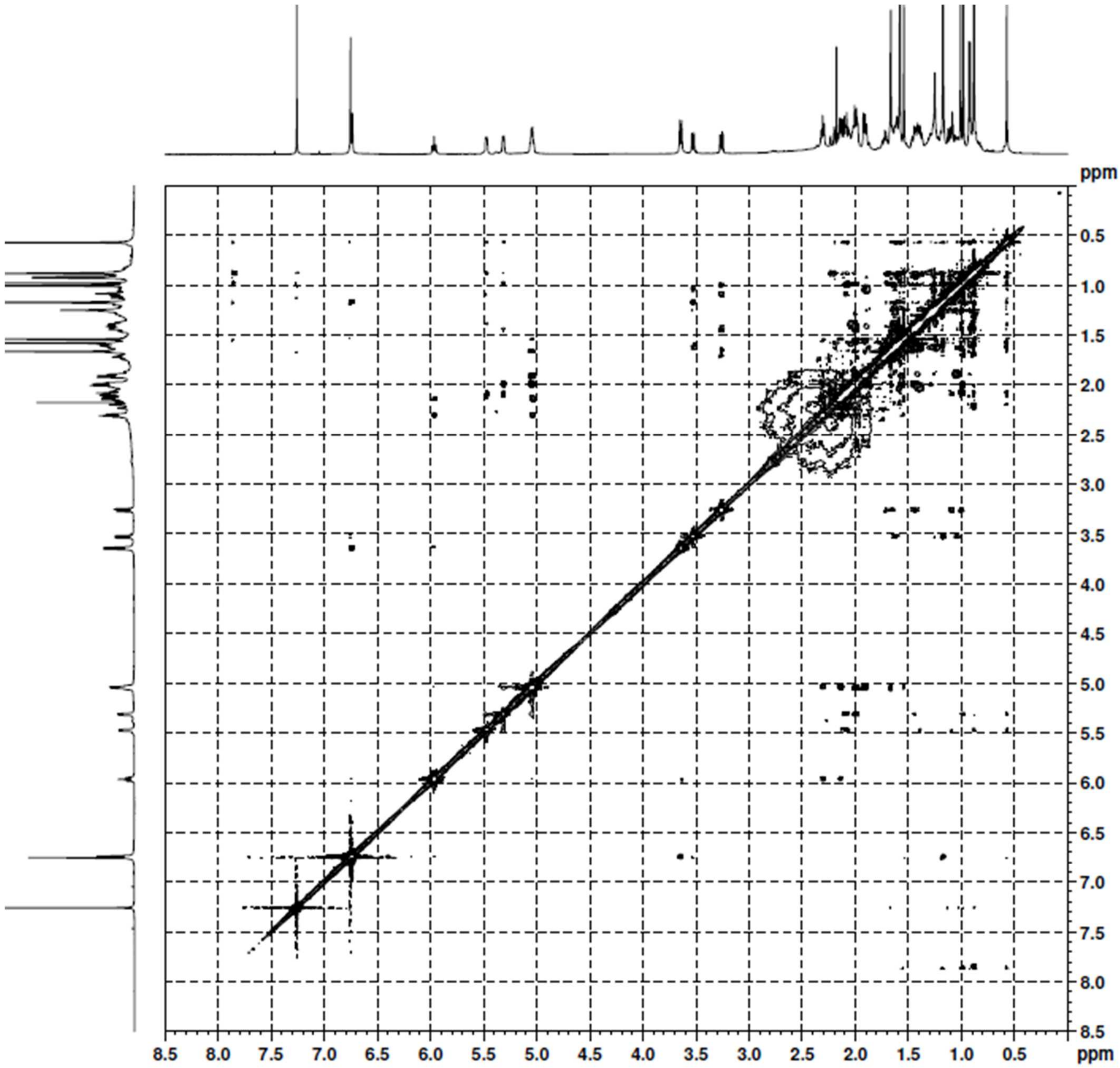

Figure S36. HRESIMS of ganohochimin D (**4**) (positive ion mode)

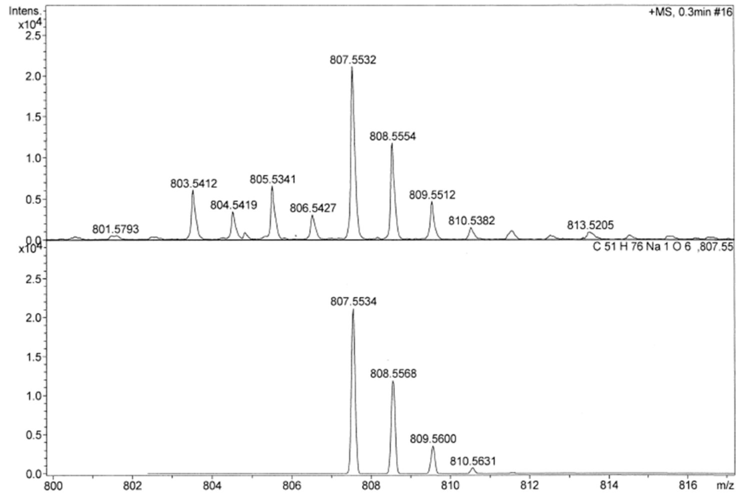

Figure S37. ^{1}H NMR spectrum of ganohochimin E (**5**) ($CDCl_3$, 500 MHz)

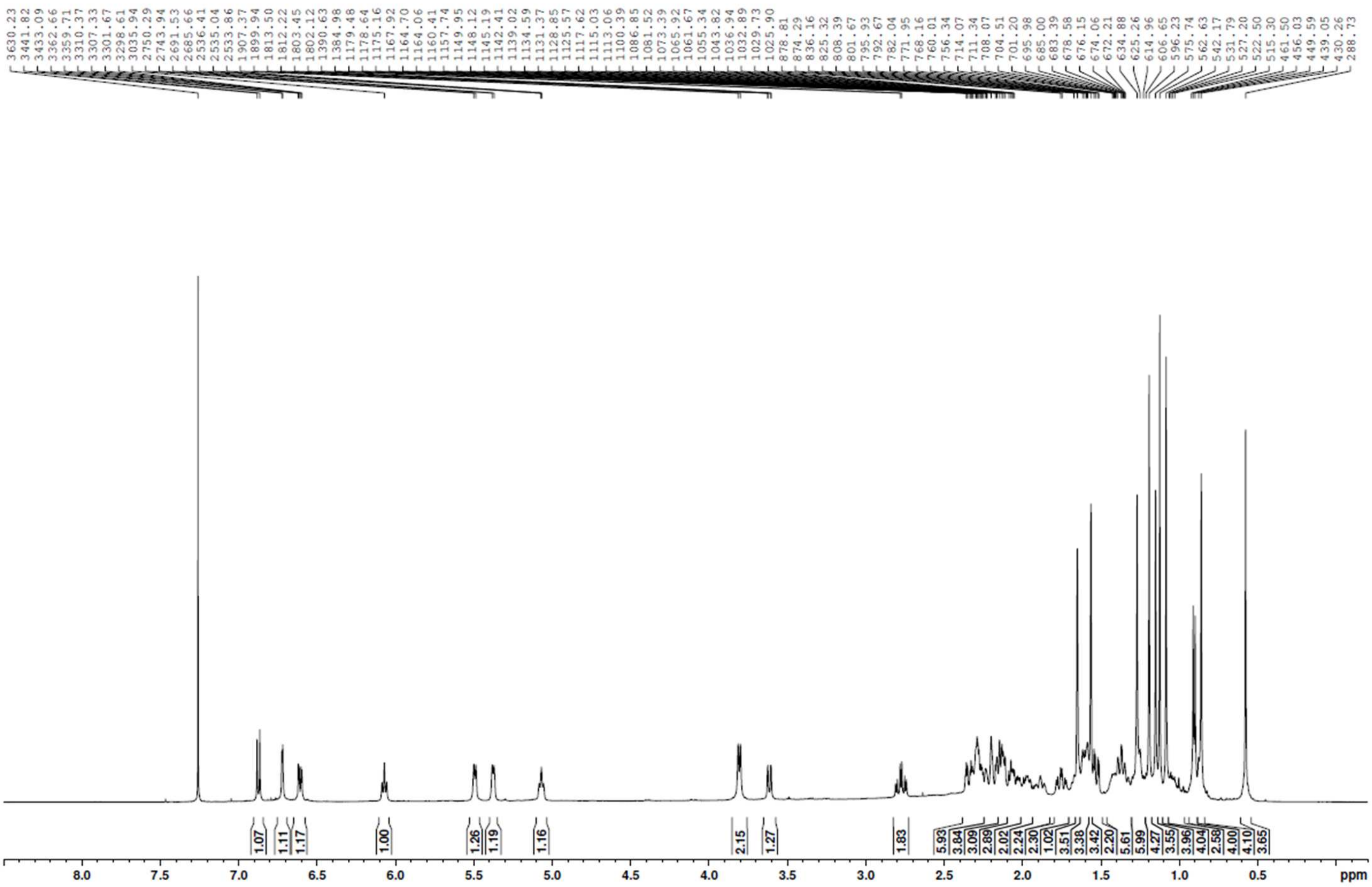

Figure S38. ^{13}C NMR spectrum of ganohochimin E (**5**) ($CDCl_3$, 125 MHz)

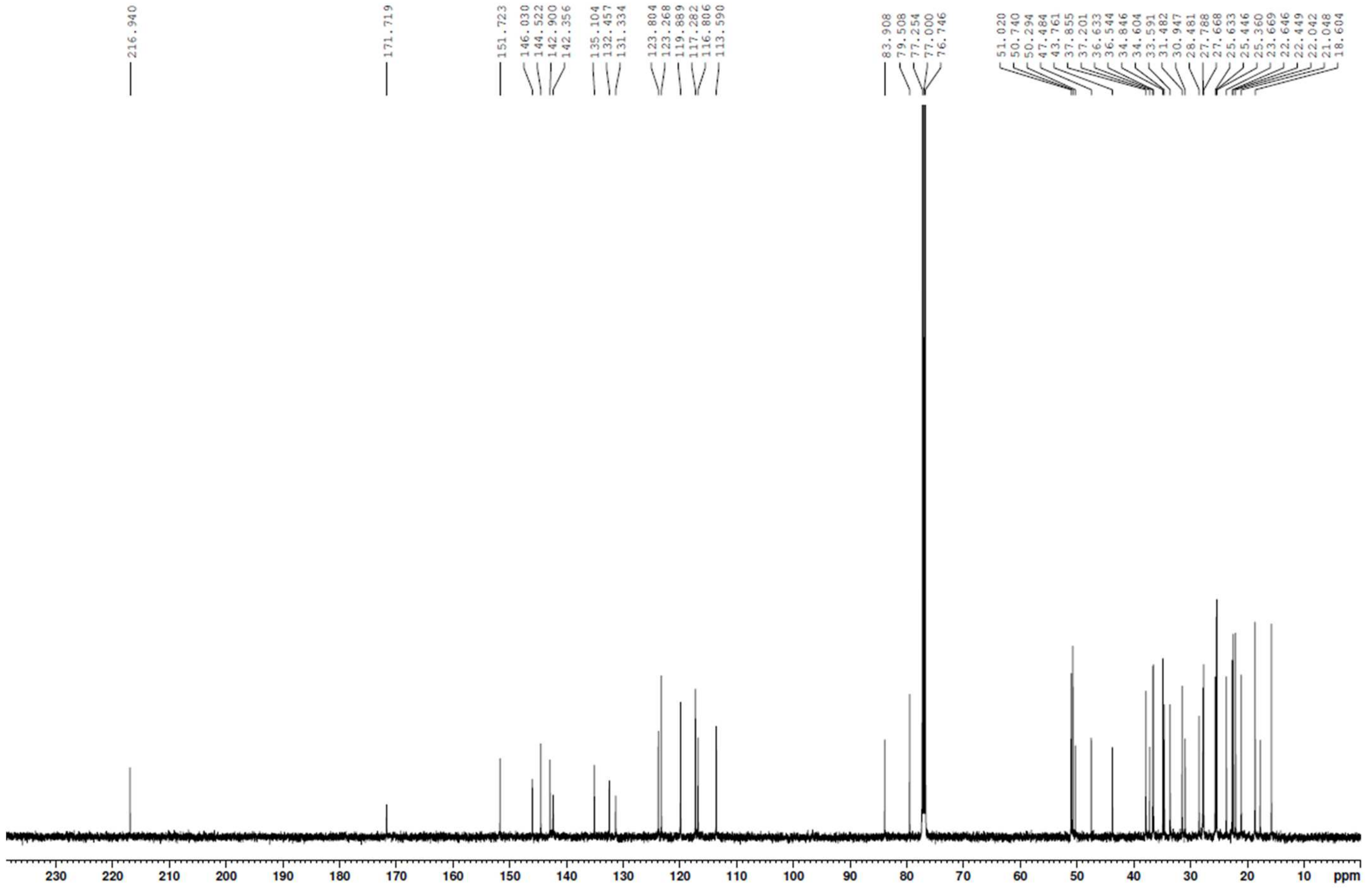

Figure S39. DEPT-135 spectrum of ganohochimin E (**5**) ($CDCl_3$, 125 MHz)

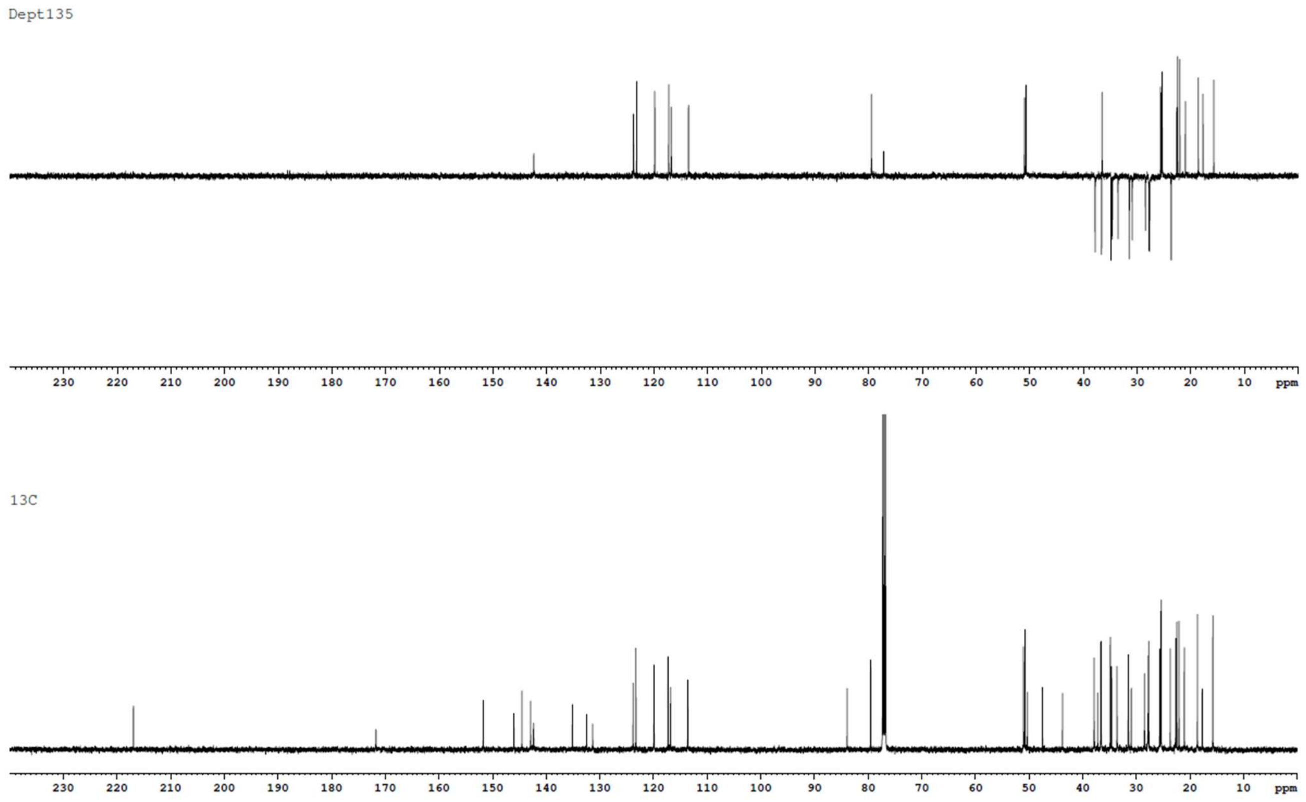

Figure S40. COSY spectrum of ganohochimin E (**5**) ($CDCl_3$, 500 MHz)

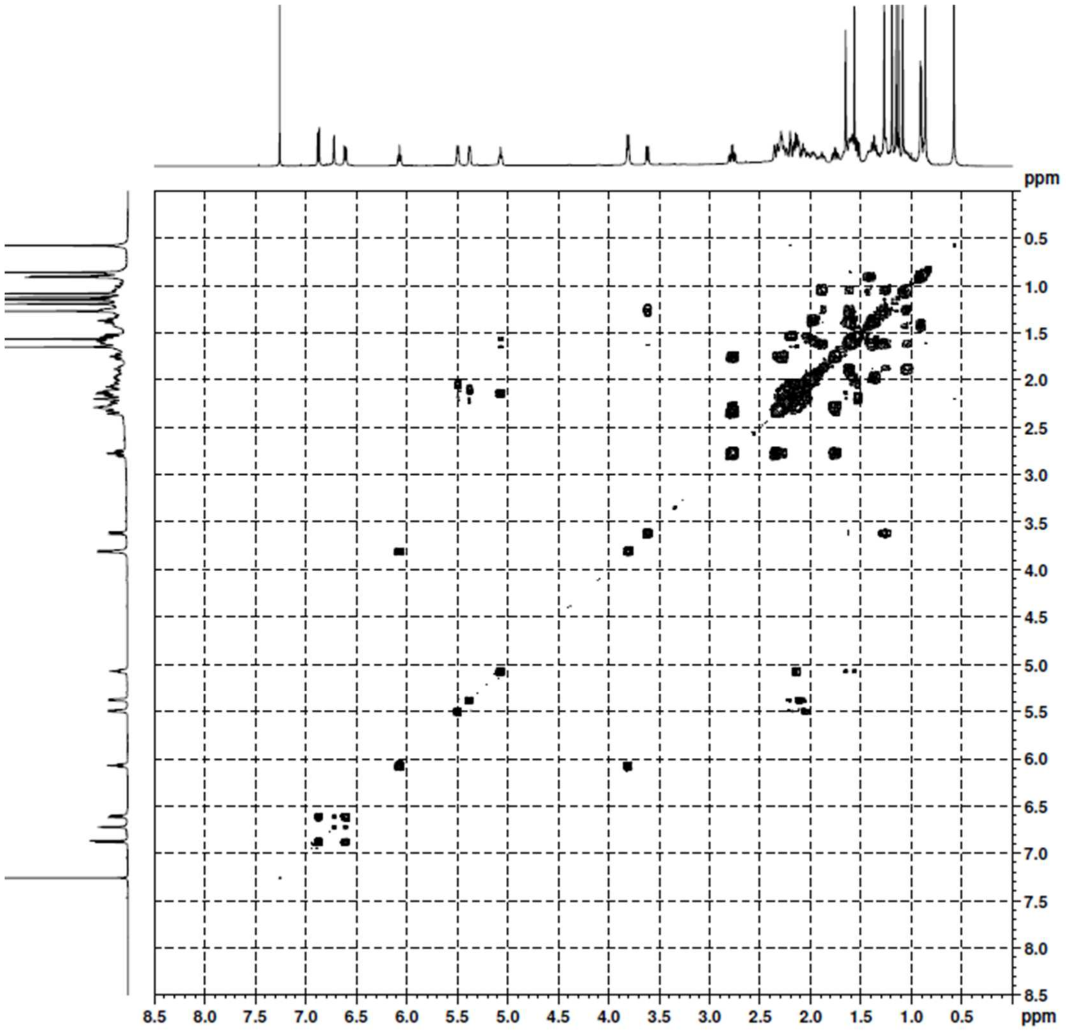

Figure S41. HSQC spectrum of ganohochimin E (**5**) (CDCl$_3$)

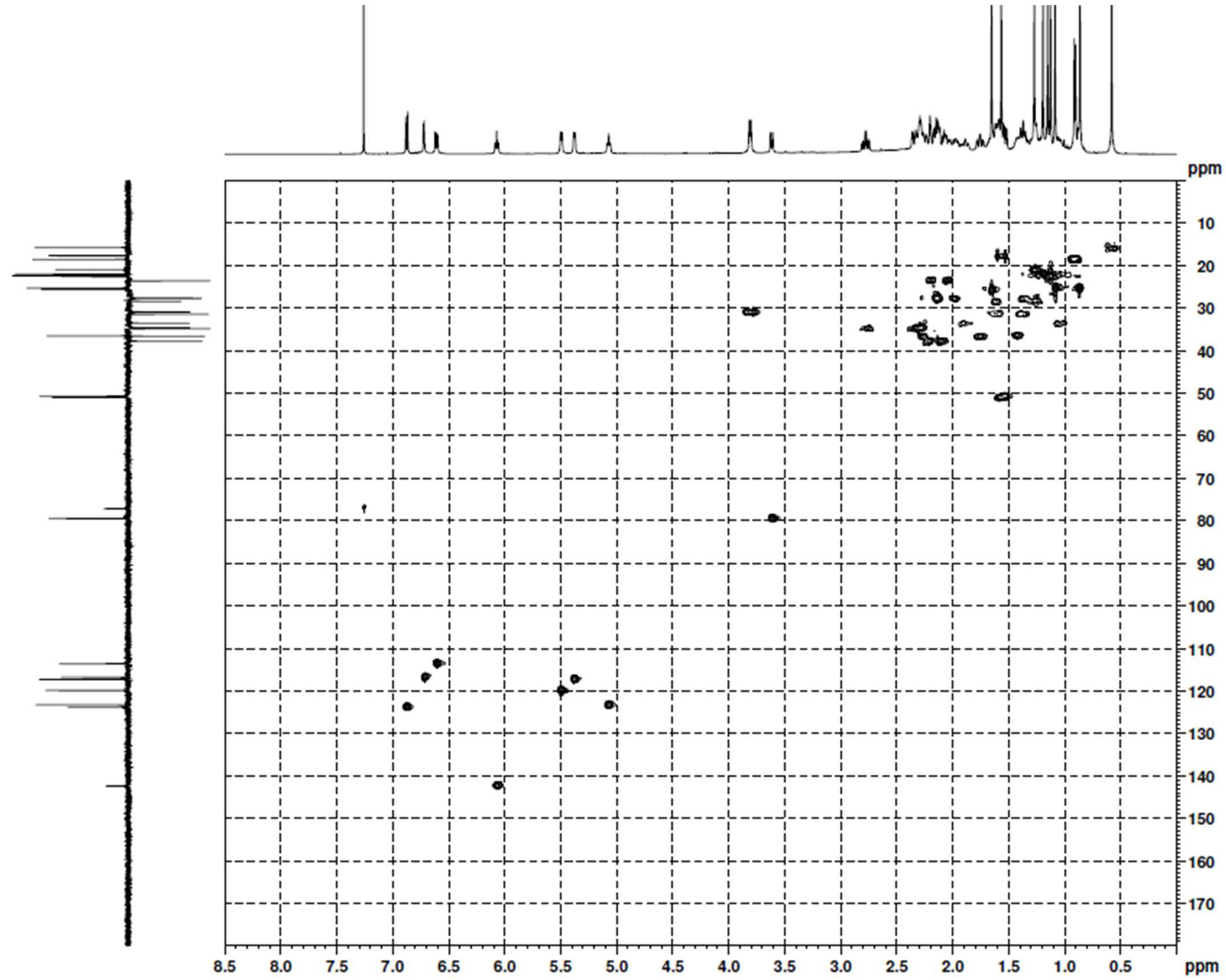

Figure S42. HMBC spectrum of ganohochimin E (**5**) ($CDCl_3$)

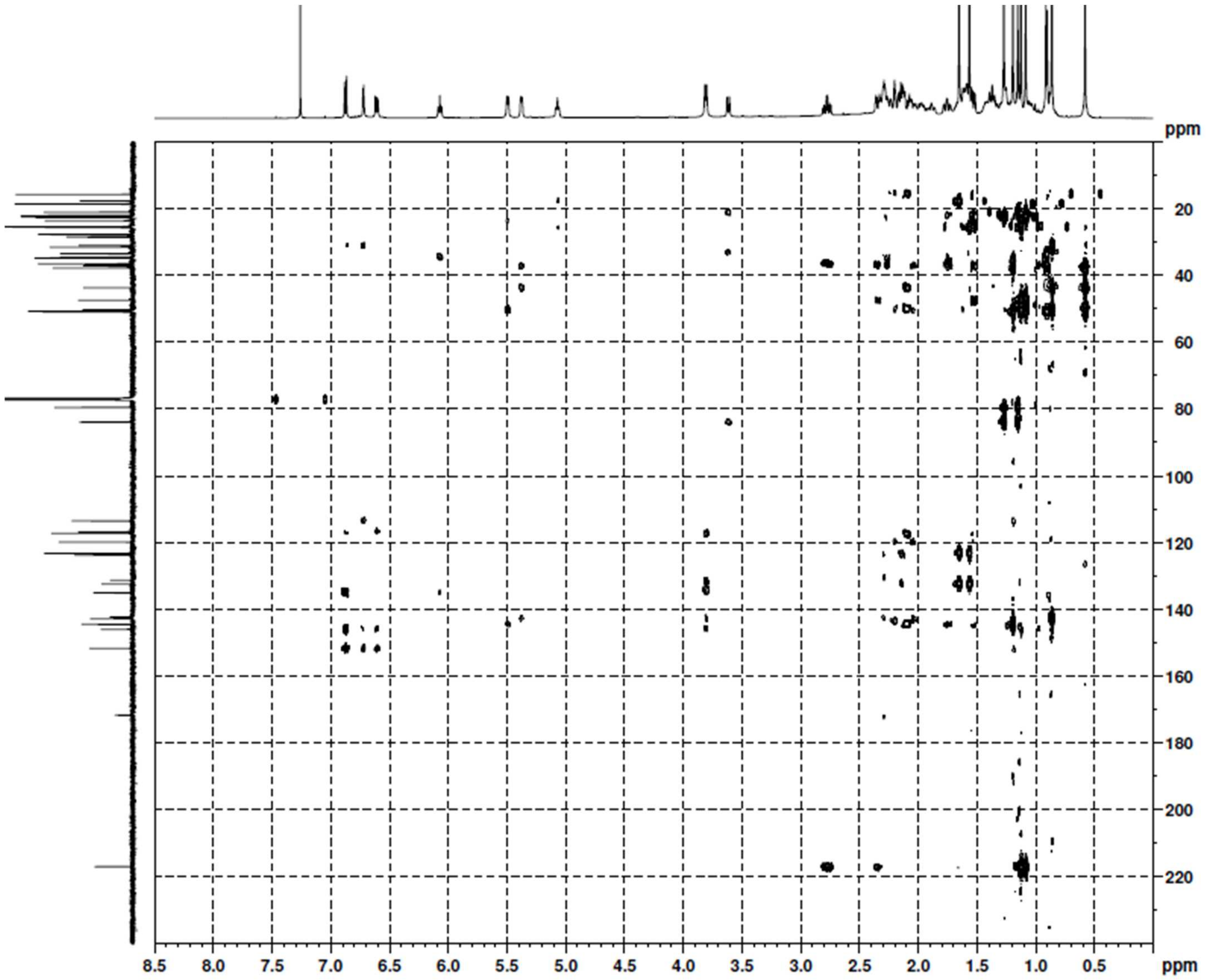

Figure S43. NOESY spectrum of ganohochimin E (**5**) ($CDCl_3$, 500 MHz)

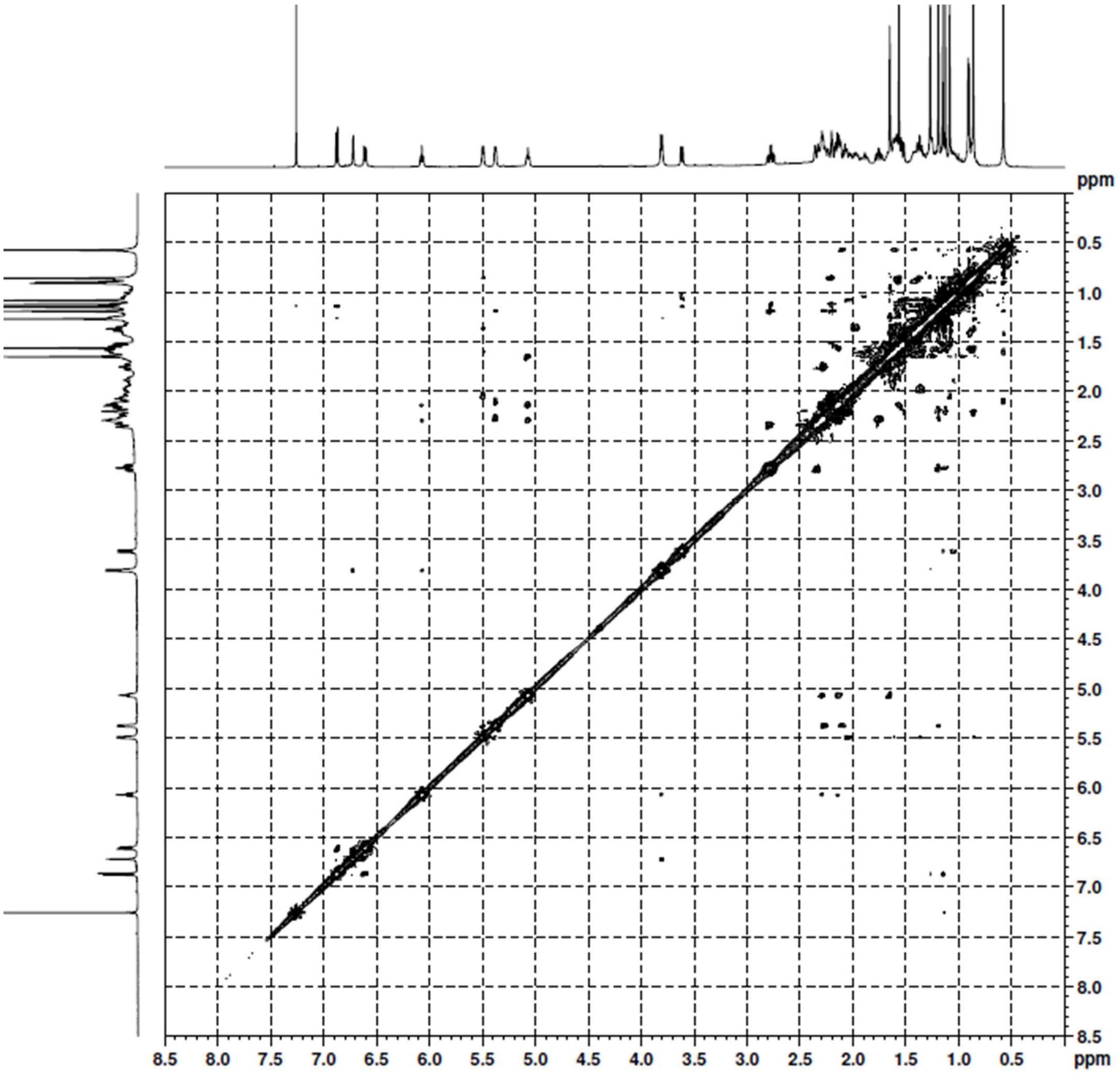

Figure S44. HRESIMS of ganohochimin E (**5**) (positive ion mode)

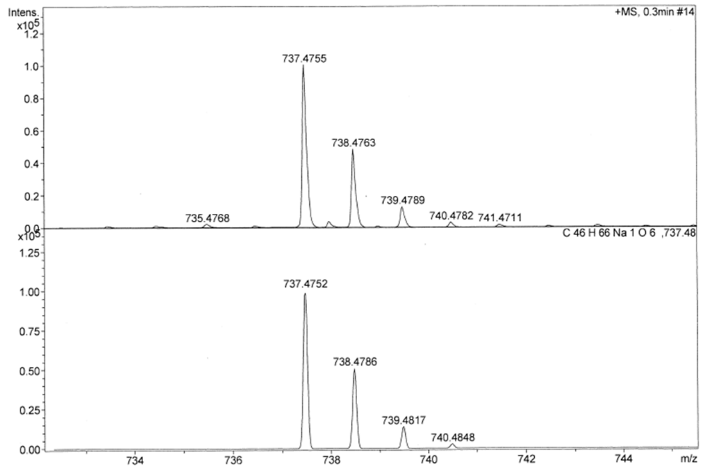

Figure S45. ^{1}H NMR spectrum of ganohochimin F (**6**) ($CDCl_3$, 500 MHz)

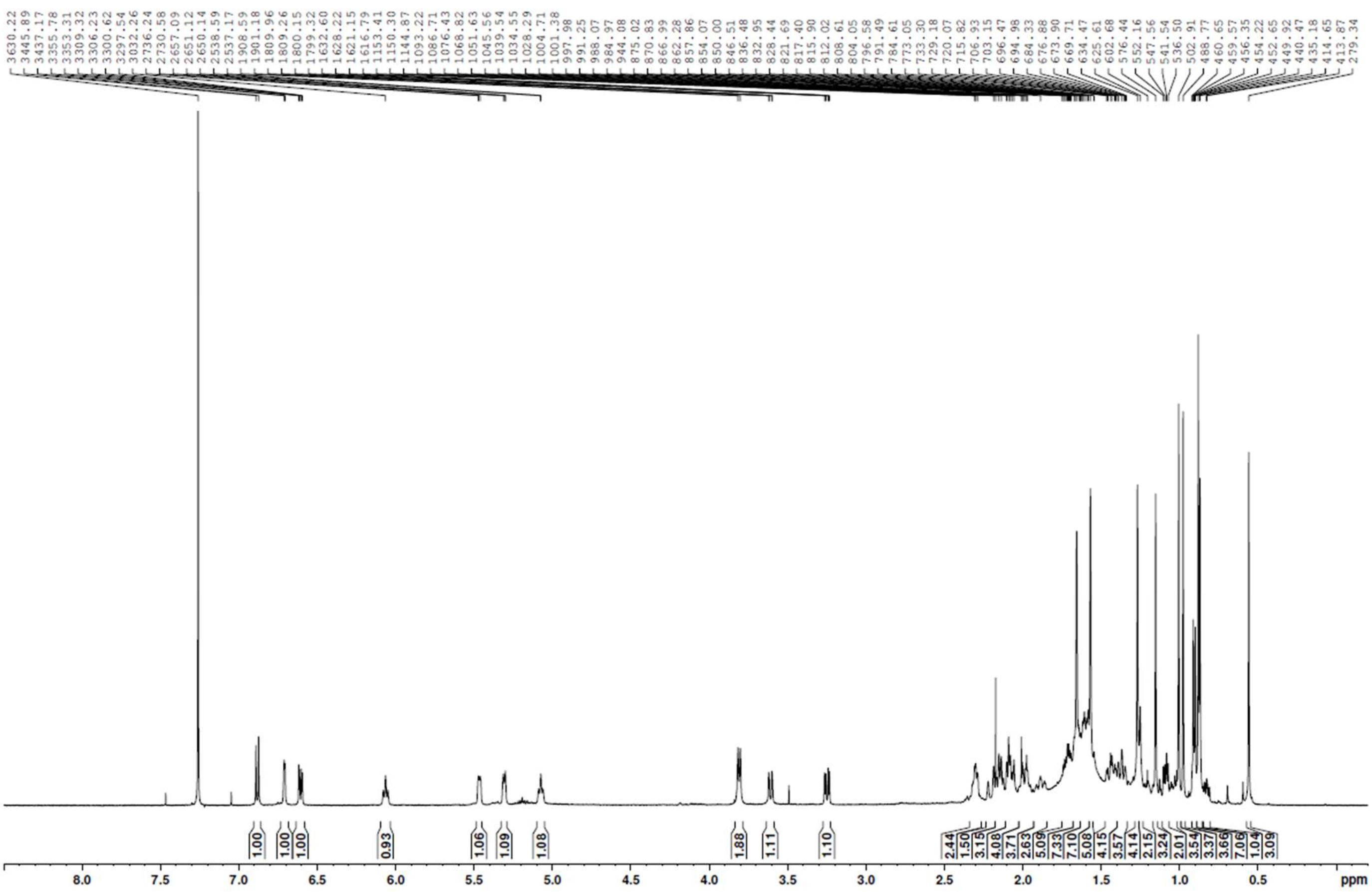

Figure S46. ^{13}C NMR spectrum of ganohochimin F (**6**) ($CDCl_3$, 125 MHz)

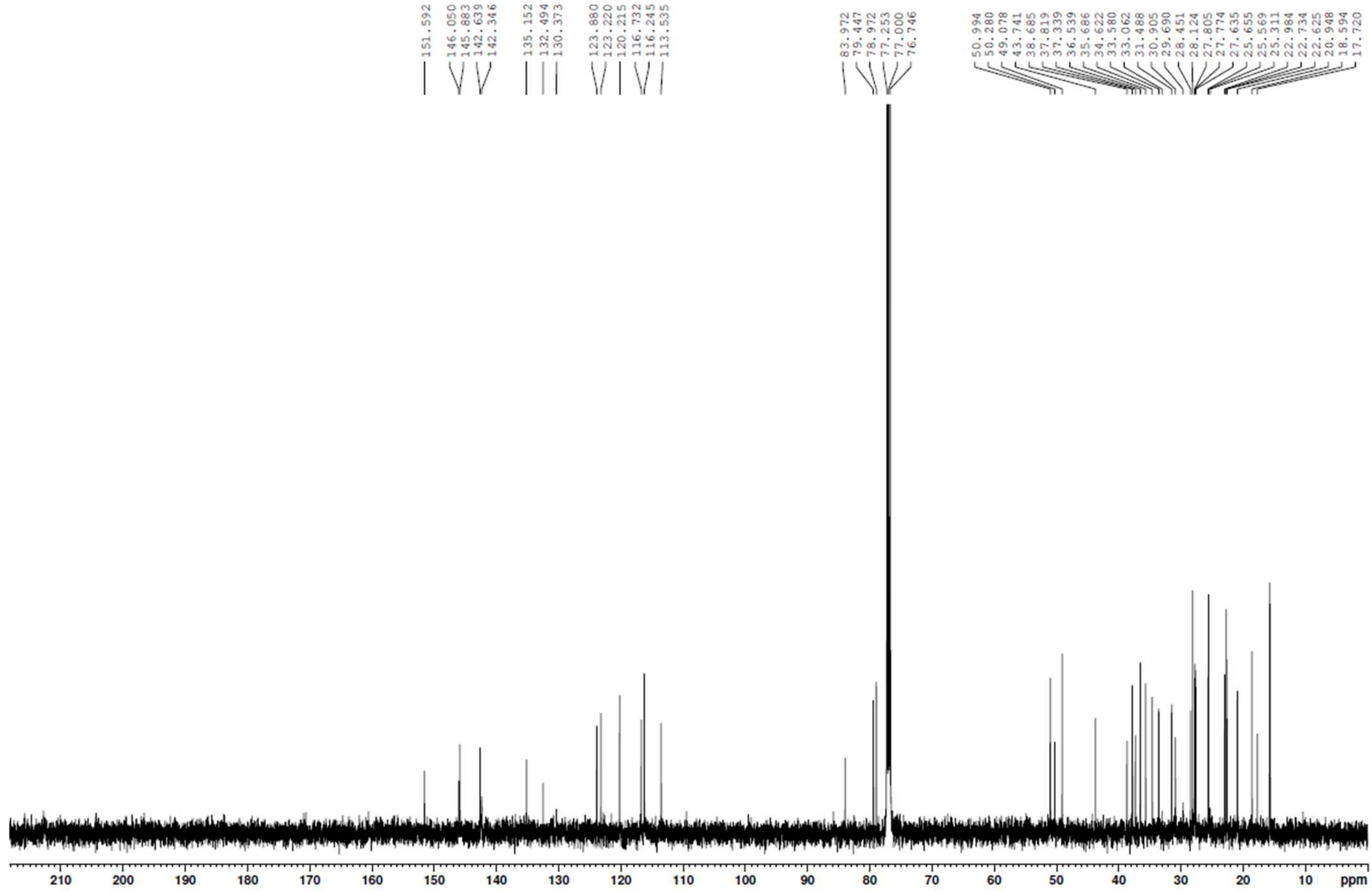

Figure S47. DEPT-135 spectrum of ganohochimin F (**6**) ($CDCl_3$, 125 MHz)

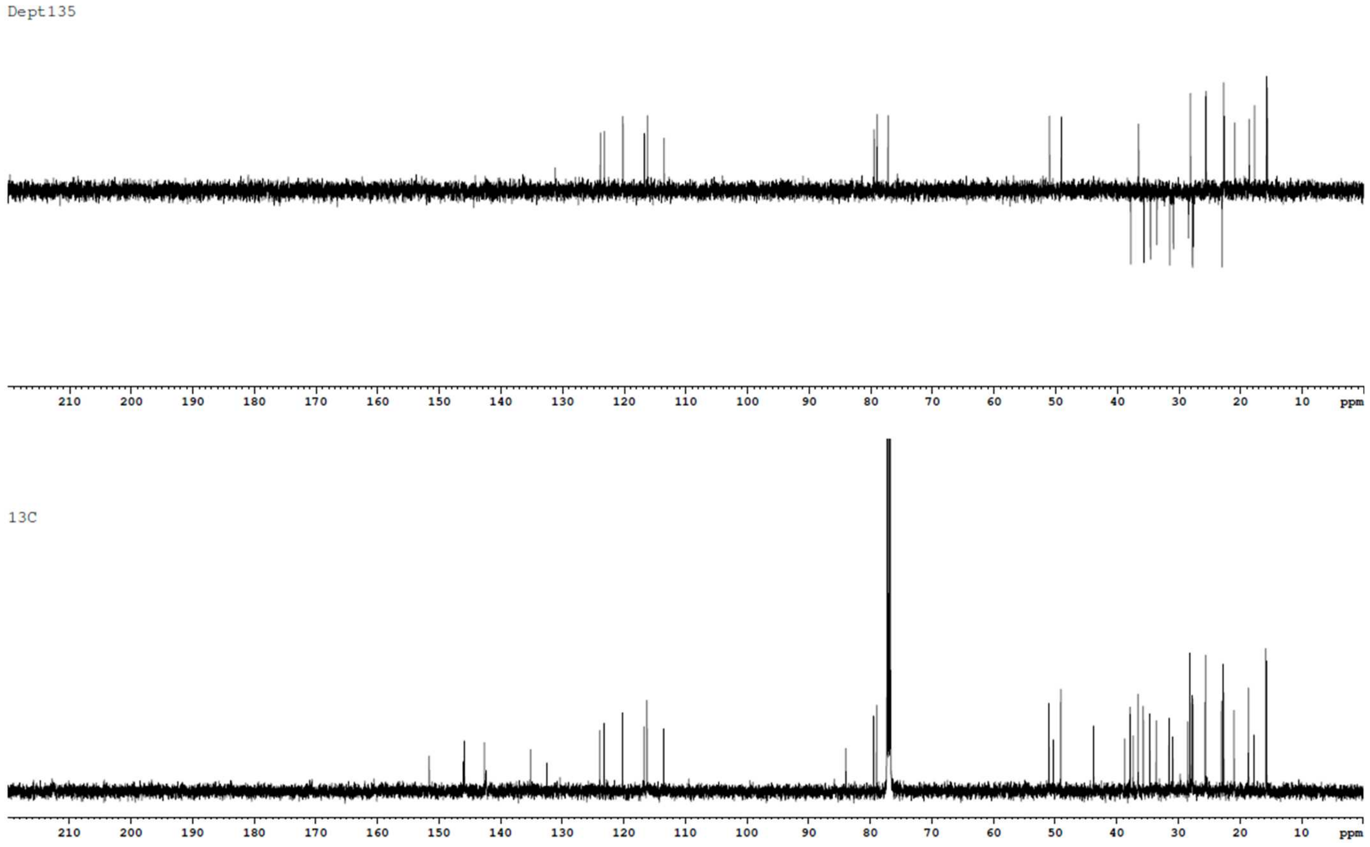

Figure S48. COSY spectrum of ganohochimin F (**6**) ($CDCl_3$, 500 MHz)

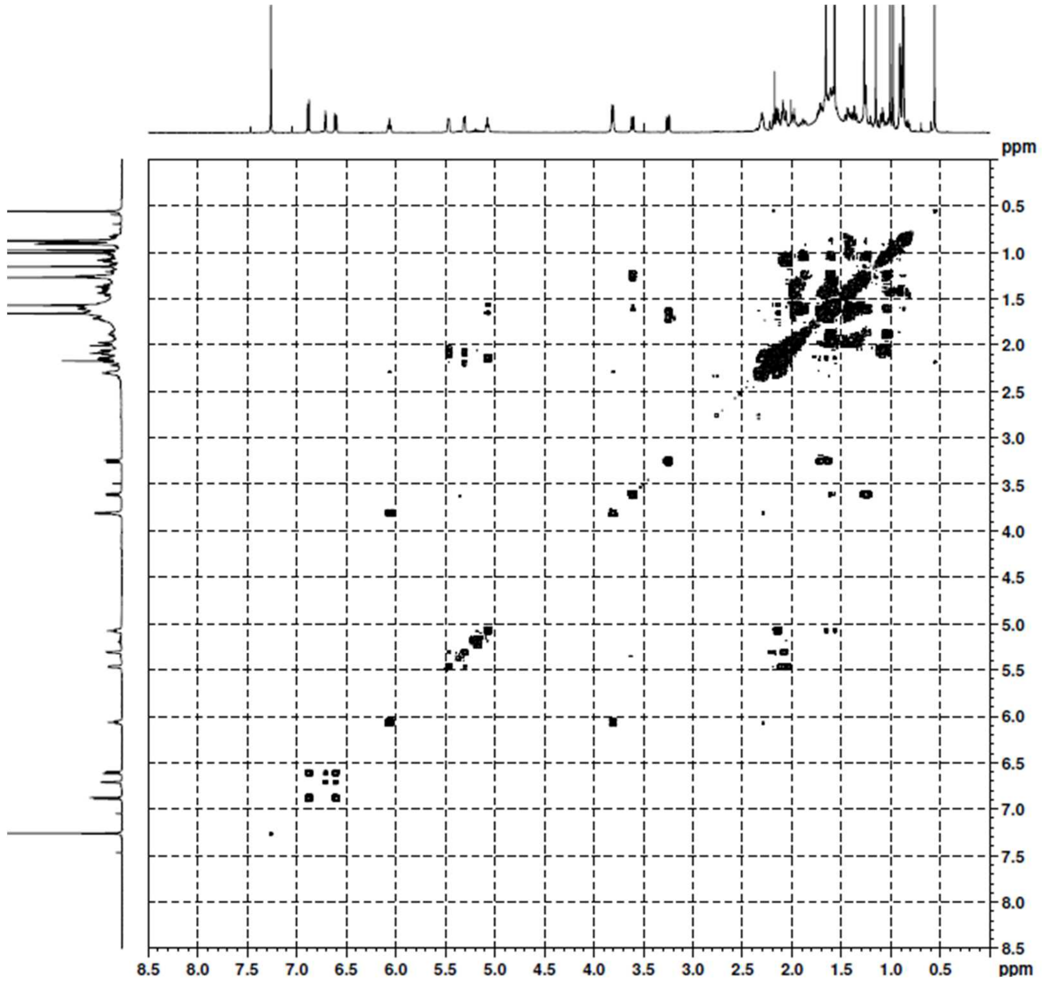

Figure S49. HSQC spectrum of ganohochimin F (**6**) ($CDCl_3$)

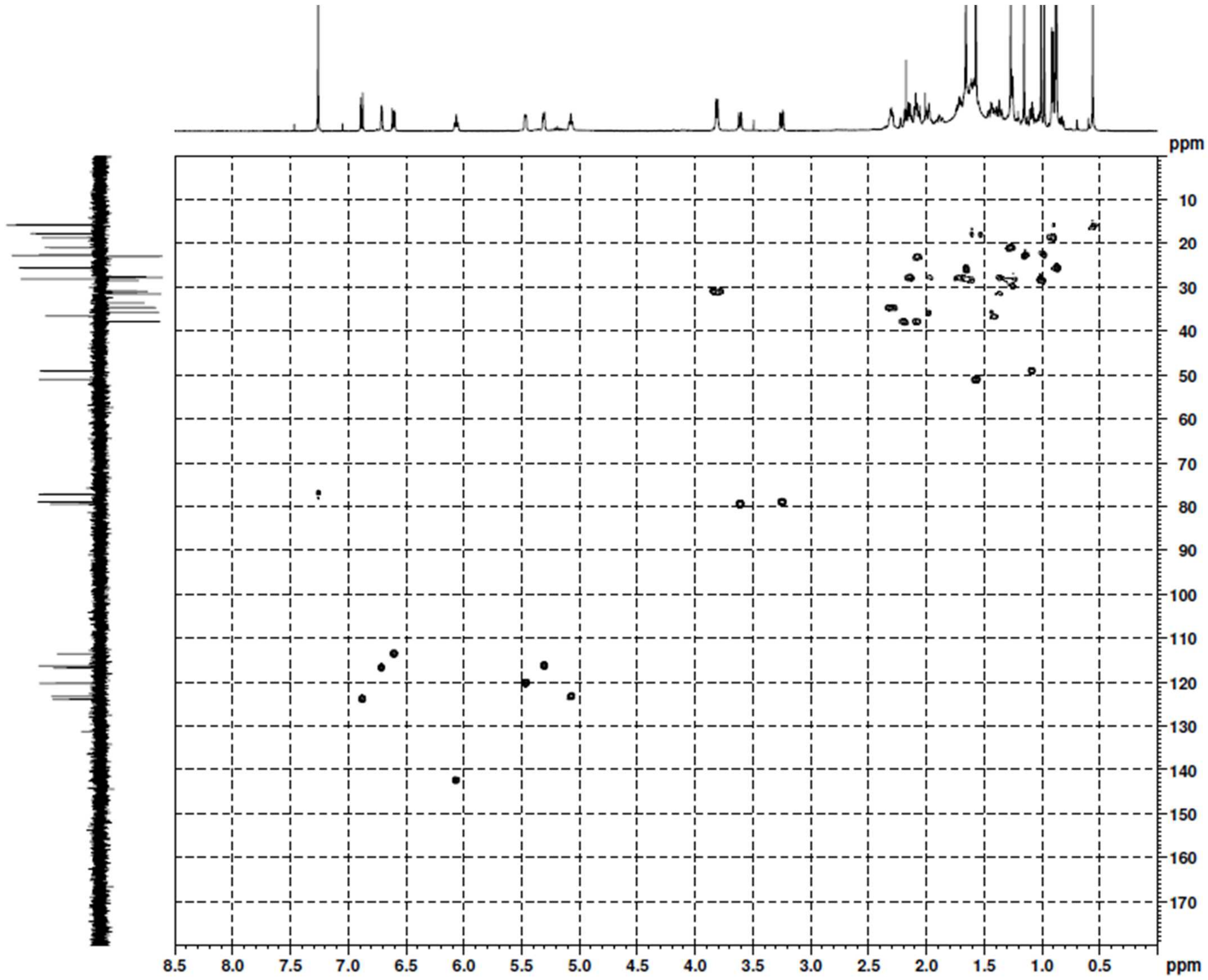

Figure S50. HMBC spectrum of ganohochimin F (**6**) ($CDCl_3$)

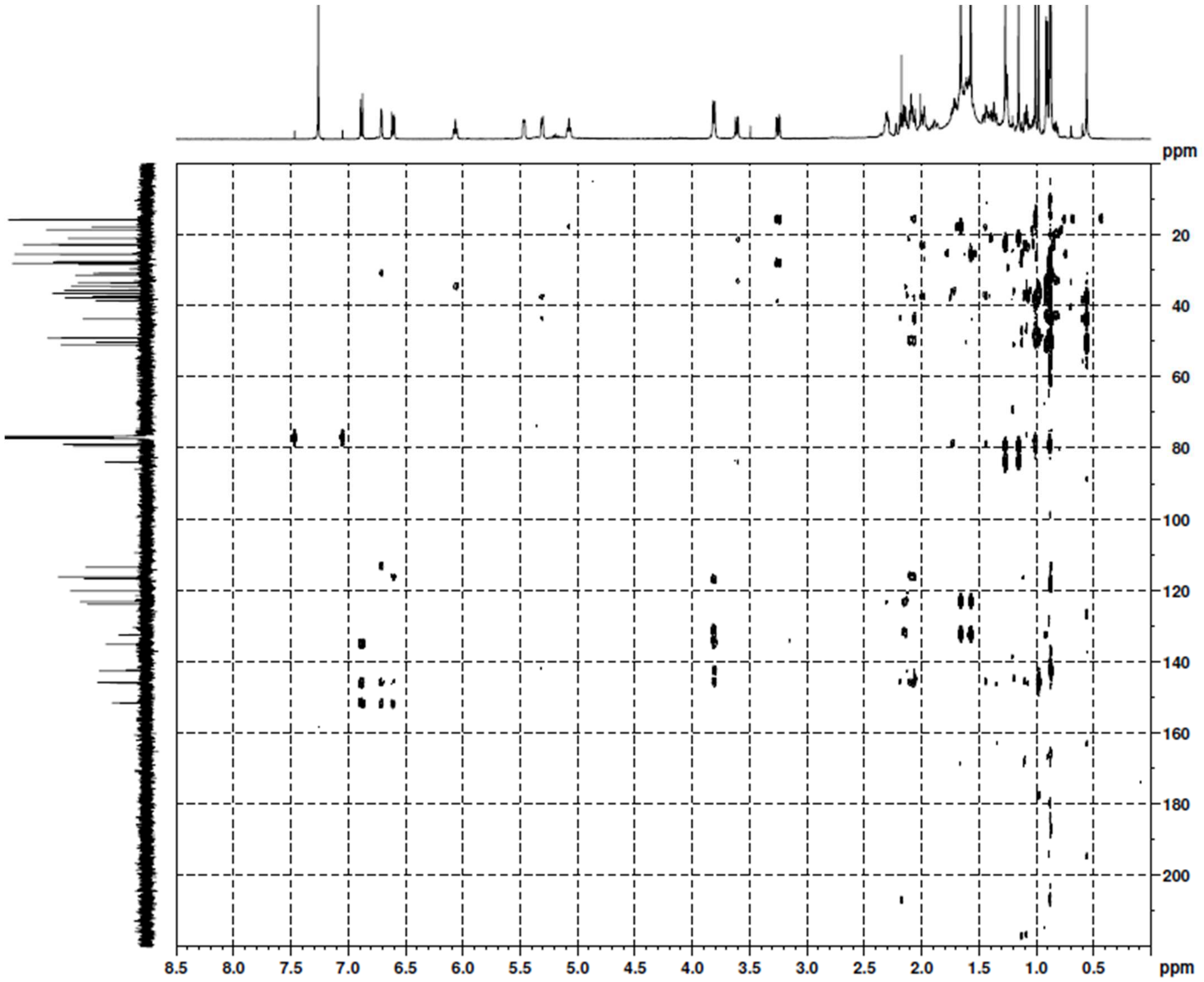

Figure S51. NOESY spectrum of ganohochimin F (**6**) ($CDCl_3$, 500 MHz)

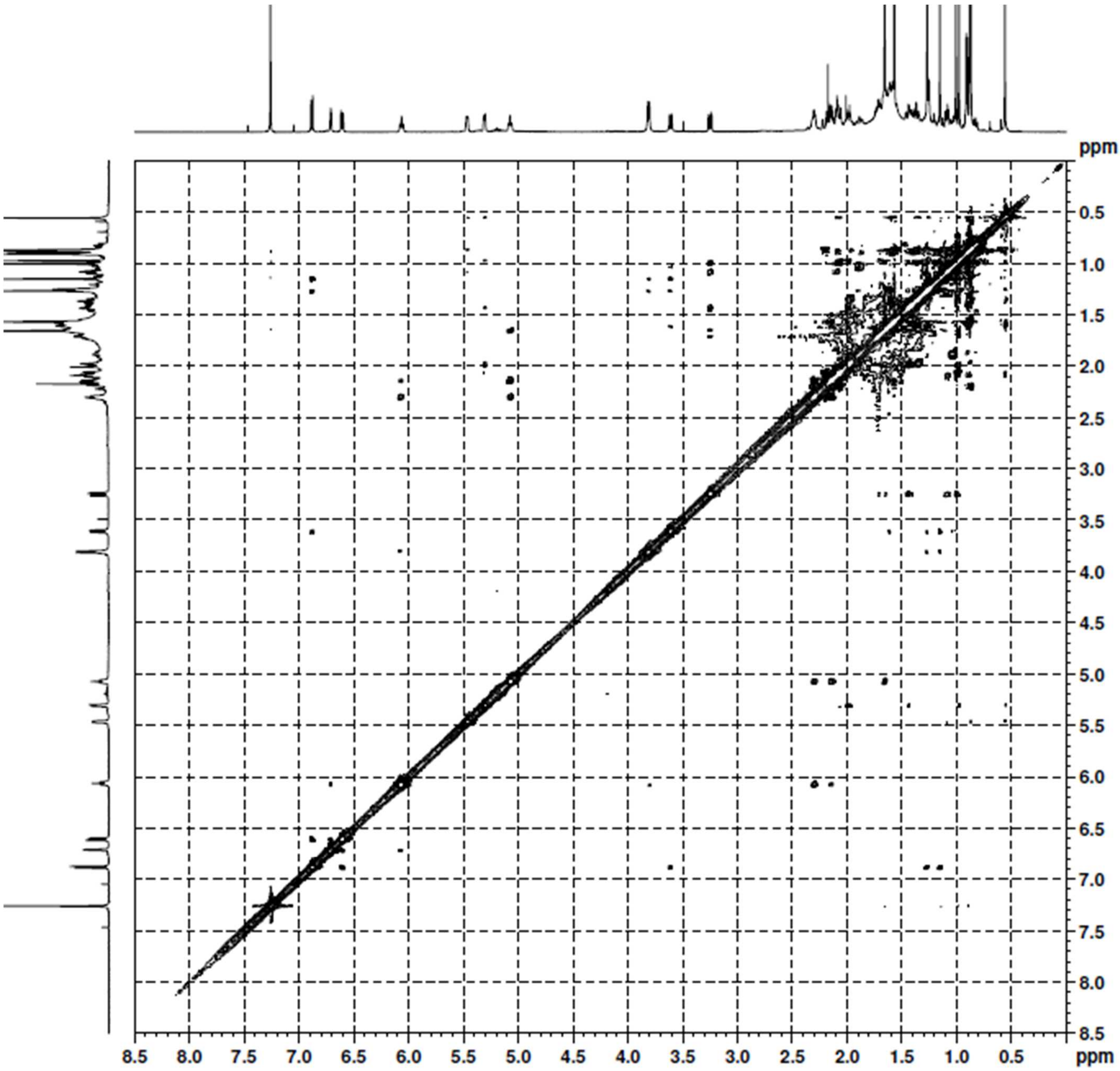

Figure S52. HRESIMS of ganohochimin F (**6**) (positive ion mode)

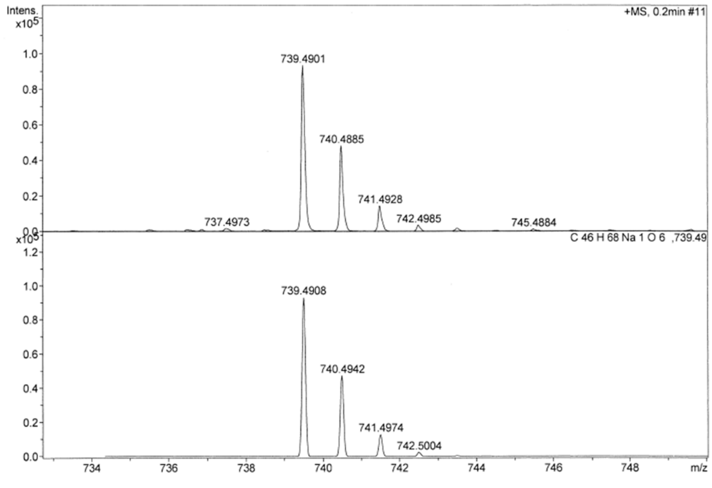

Figure S53. ^{1}H NMR spectrum of ganohochimin G (**7**) ($CDCl_3$, 500 MHz)

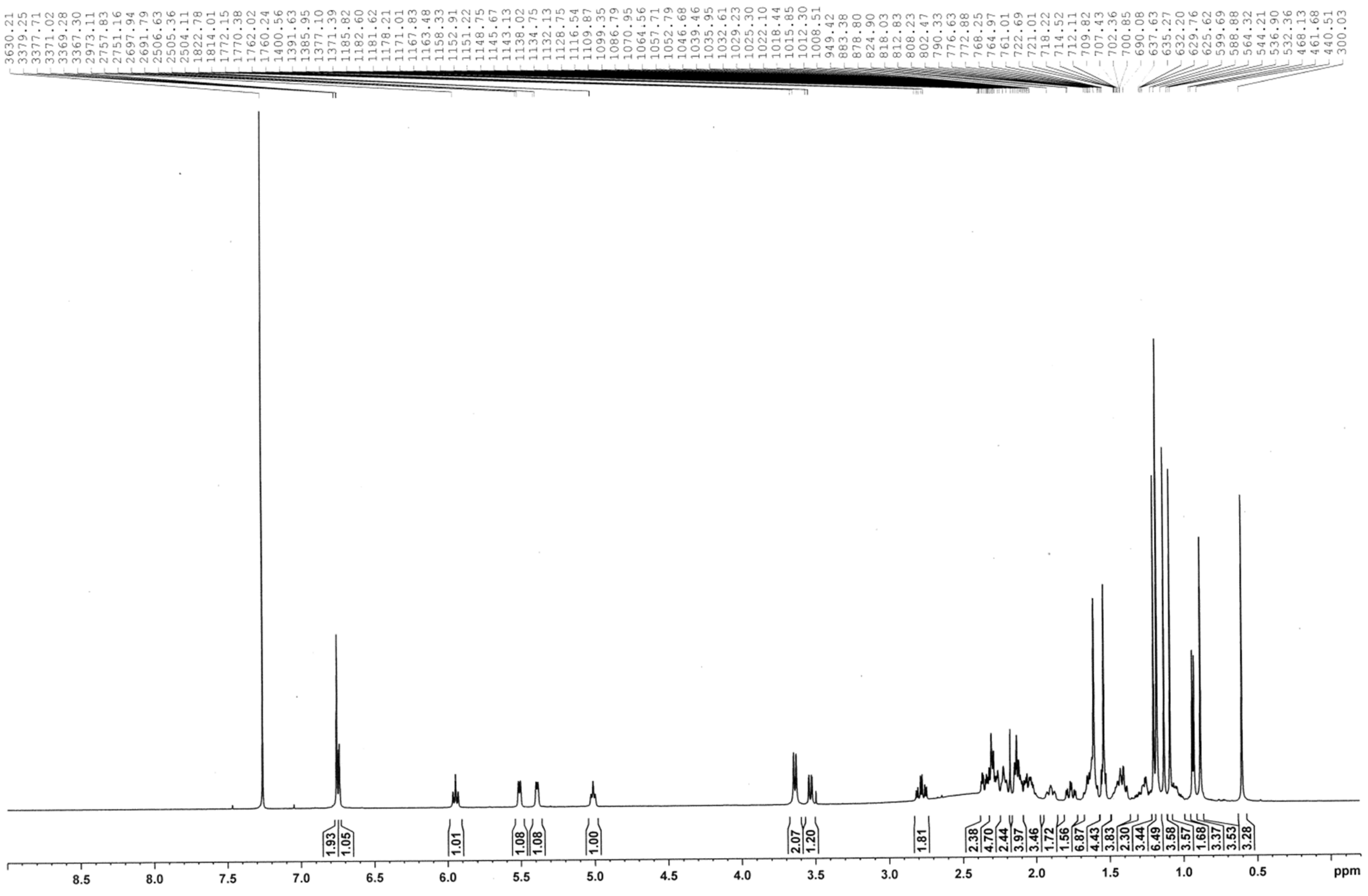

Figure S54. ^{13}C NMR spectrum of ganohochimin G (**7**) ($CDCl_3$, 125 MHz)

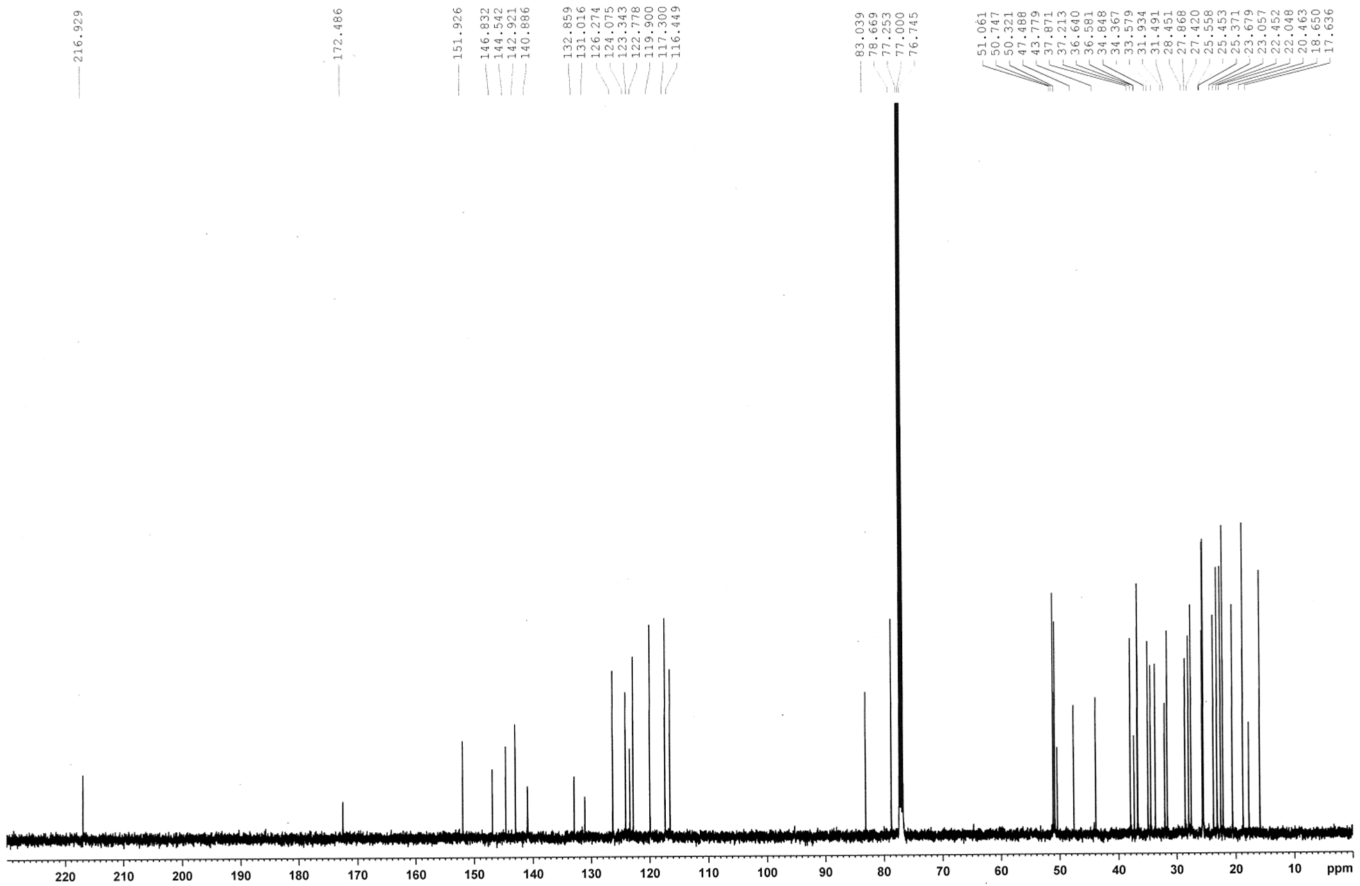

Figure S55. DEPT-135 spectrum of ganohochimin G (**7**) ($CDCl_3$, 125 MHz)

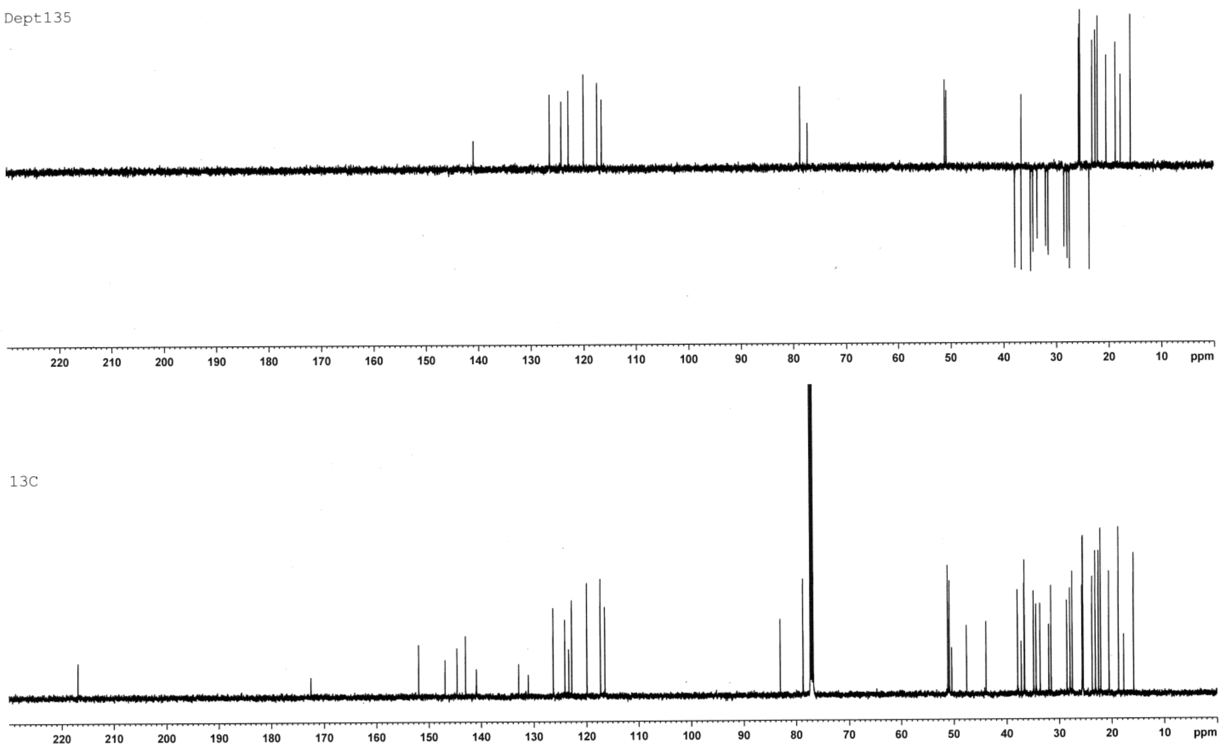

Figure S56. COSY spectrum of ganohochimin G (**7**) ($CDCl_3$, 500 MHz)

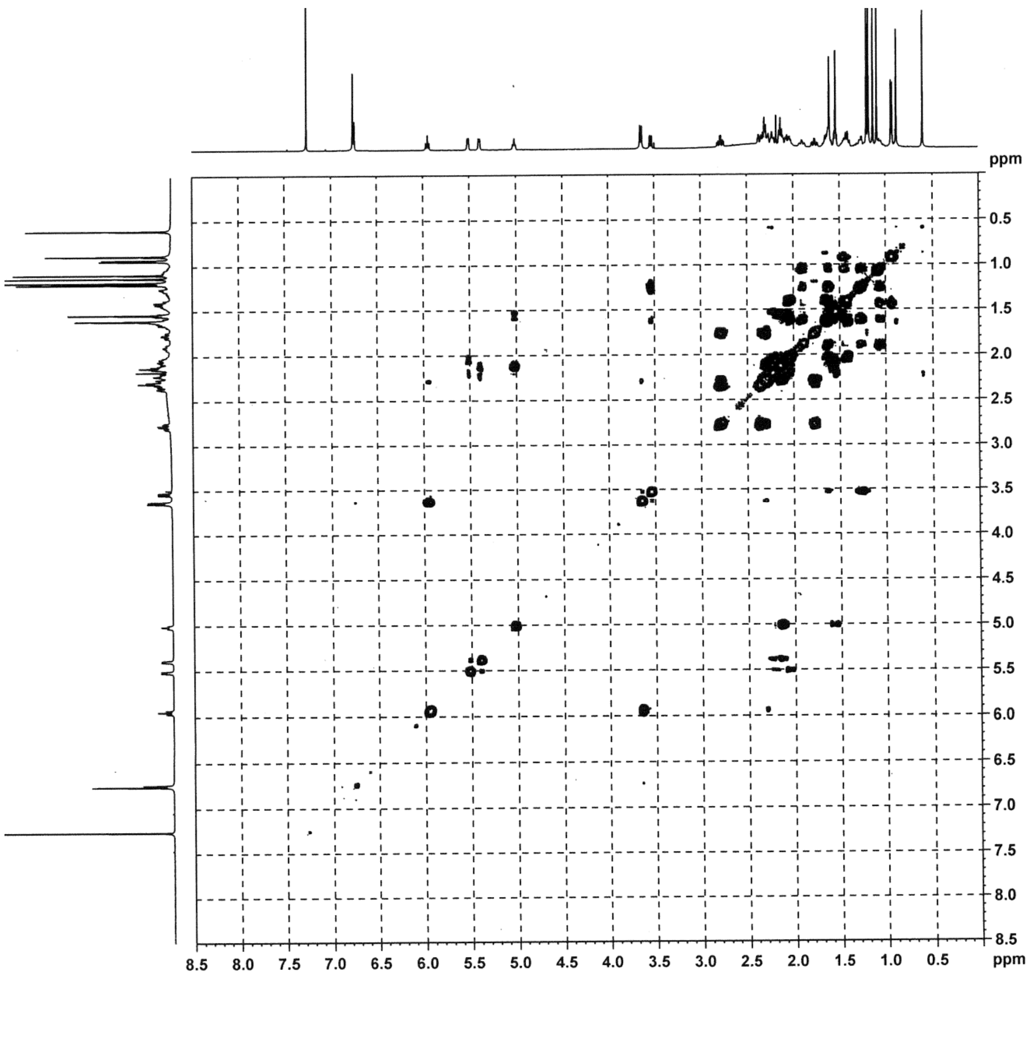

Figure S57. HSQC spectrum of ganohochimin G (**7**) ($CDCl_3$)

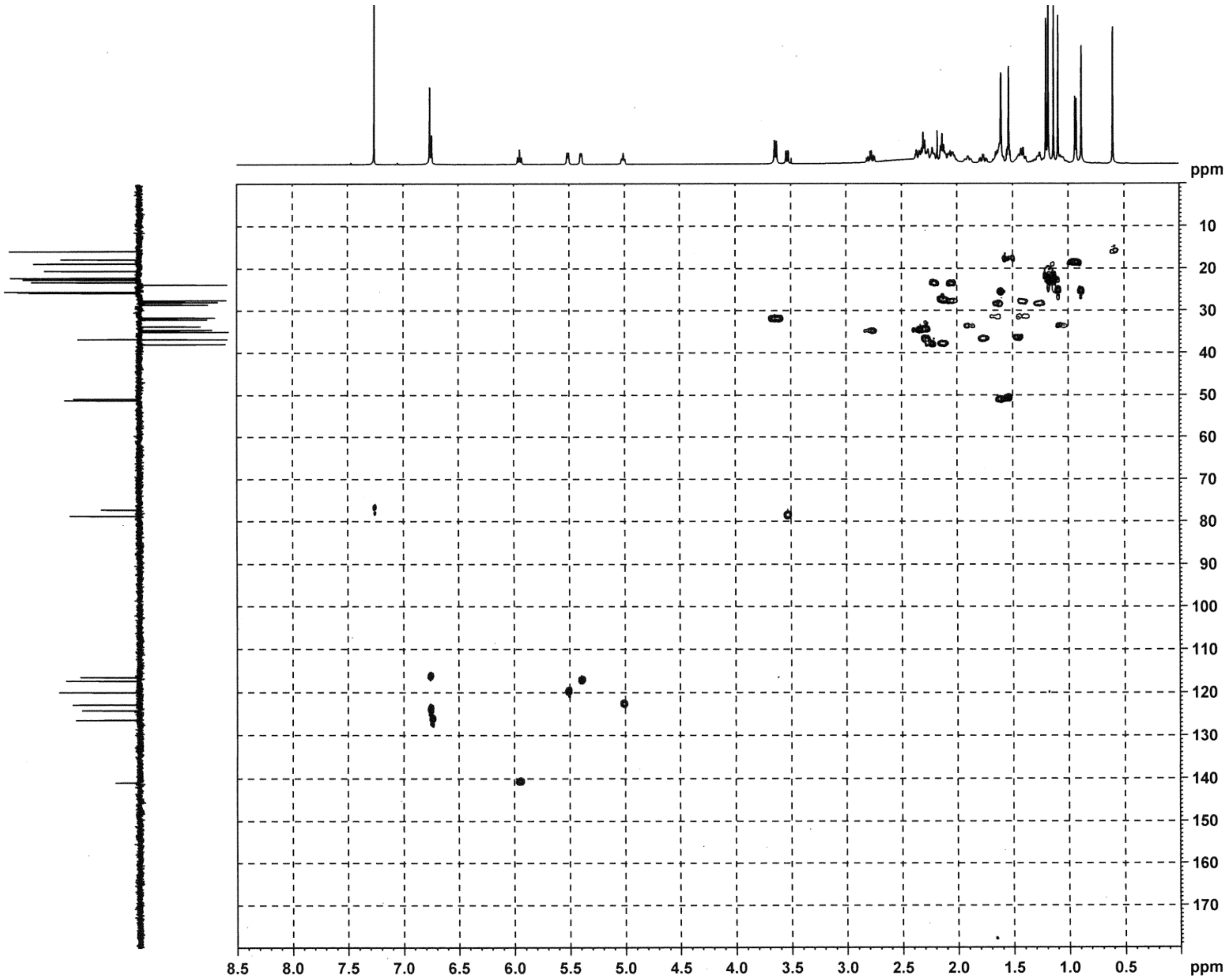

Figure S58. HMBC spectrum of ganohochimin G (**7**) ($CDCl_3$)

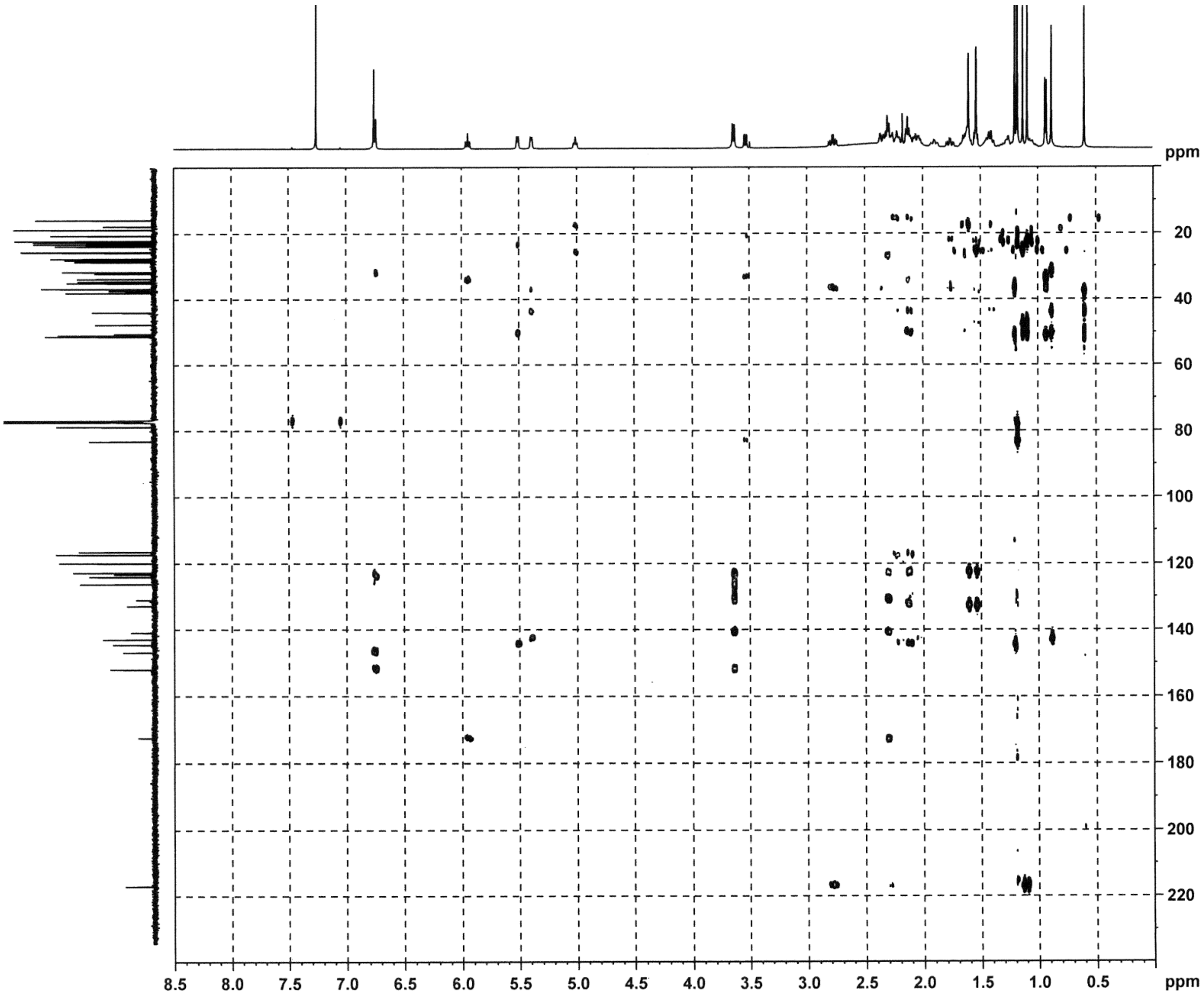

Figure S59. NOESY spectrum of ganohochimin G (**7**) ($CDCl_3$, 500 MHz)

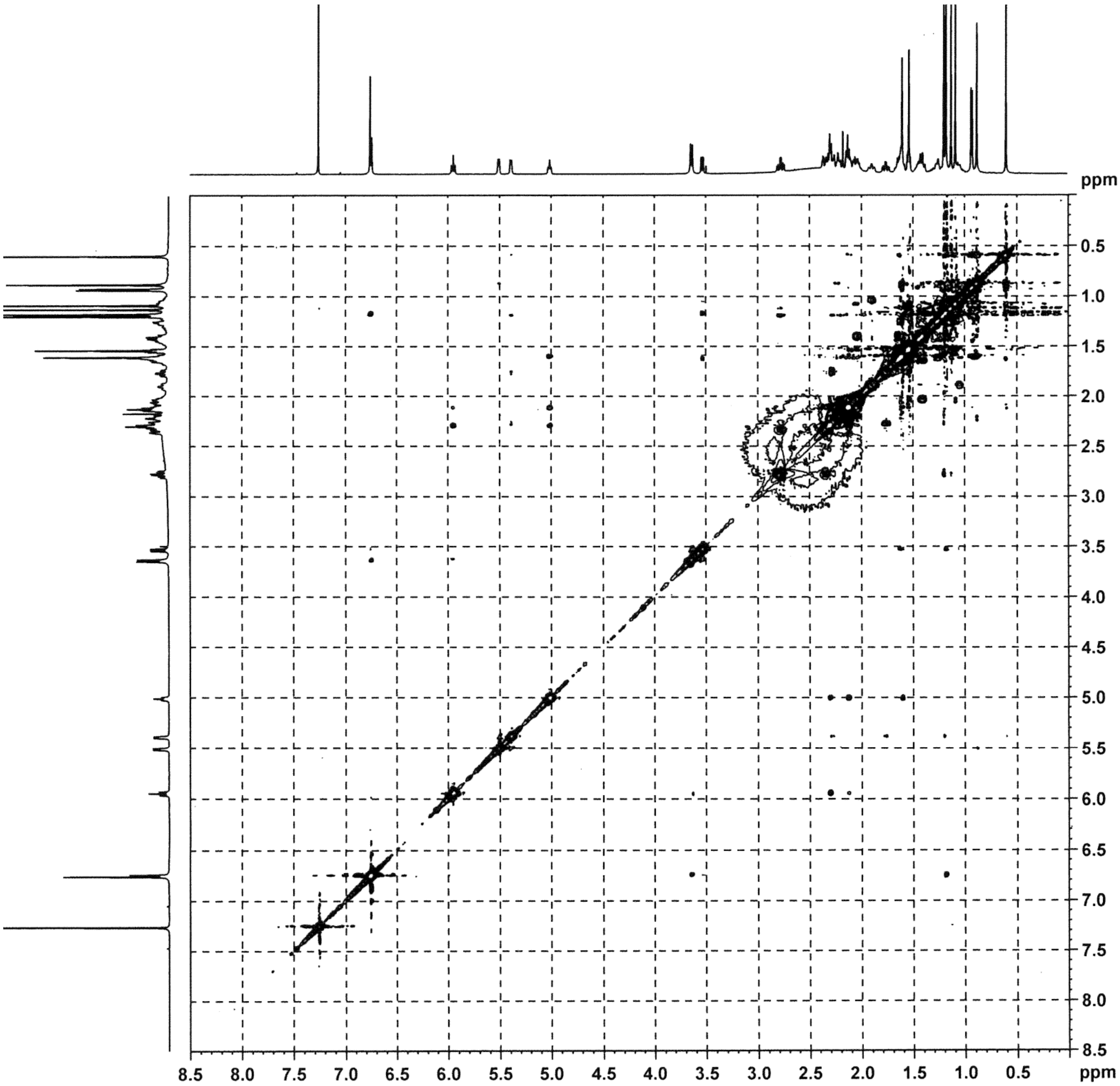

Figure S60. HRESIMS of ganohochimin G (**7**) (positive ion mode)

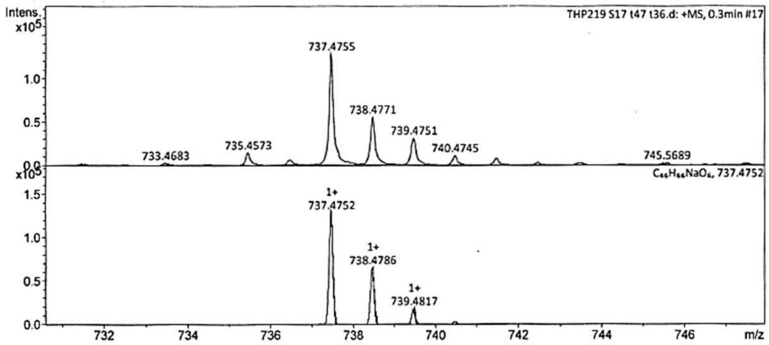

Figure S61. ^{1}H NMR spectrum of ganohochimin H (**8**) ($CDCl_3$, 500 MHz)

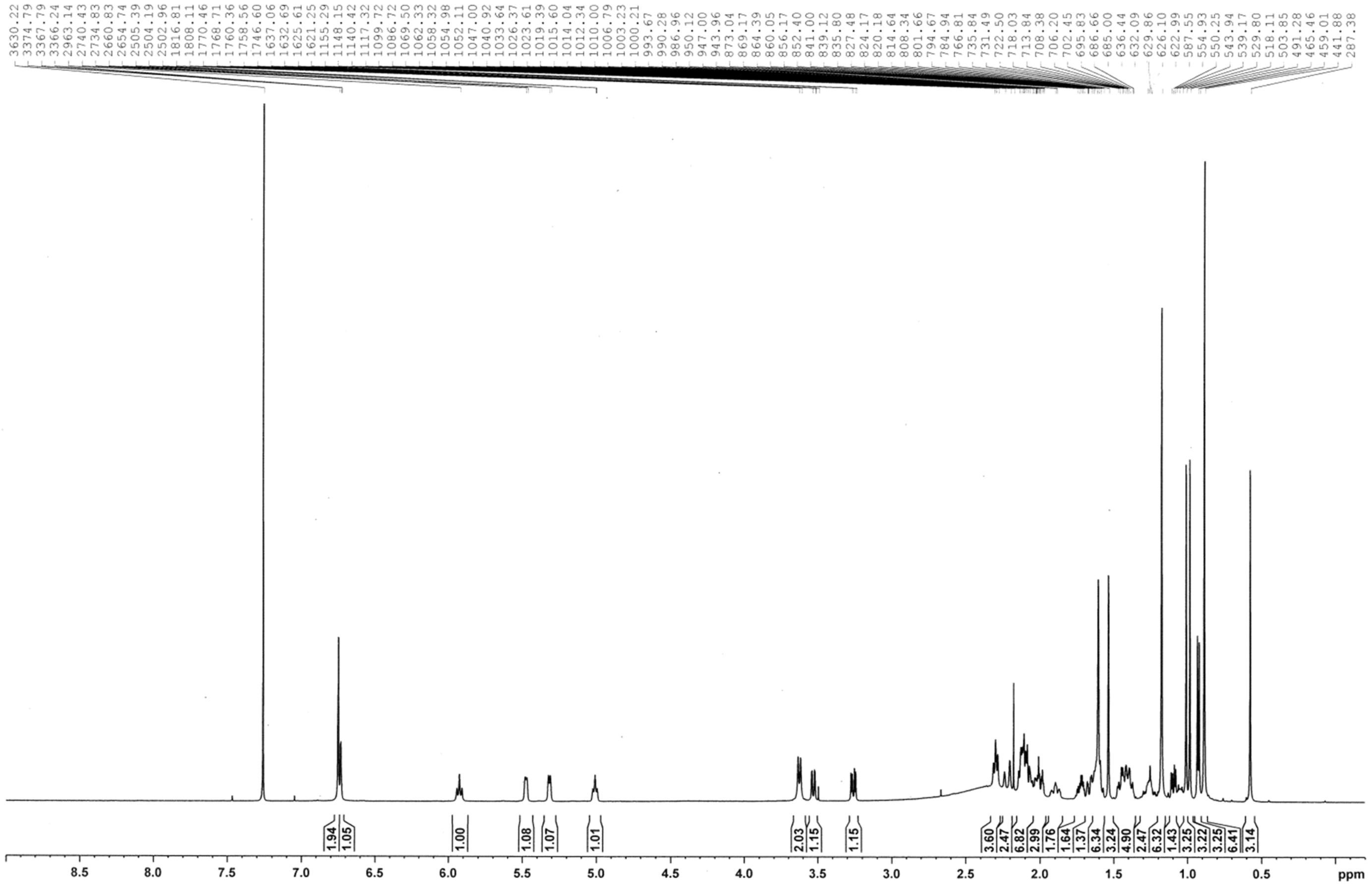

Figure S62. ^{13}C NMR spectrum of ganohochimin H (**8**) ($CDCl_3$, 125 MHz)

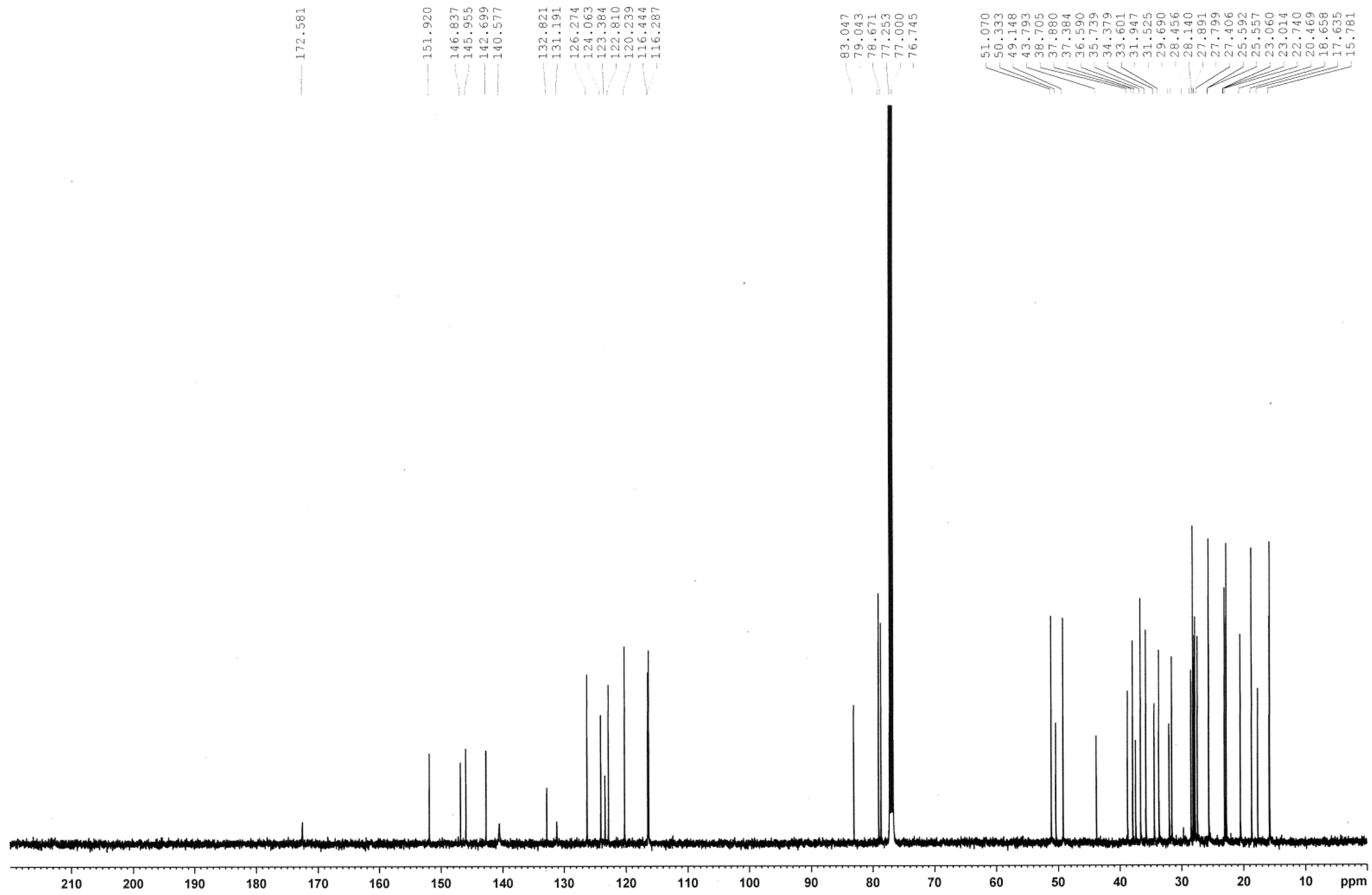

Figure S63. DEPT-135 spectrum of ganohochimin H (**8**) ($CDCl_3$, 125 MHz)

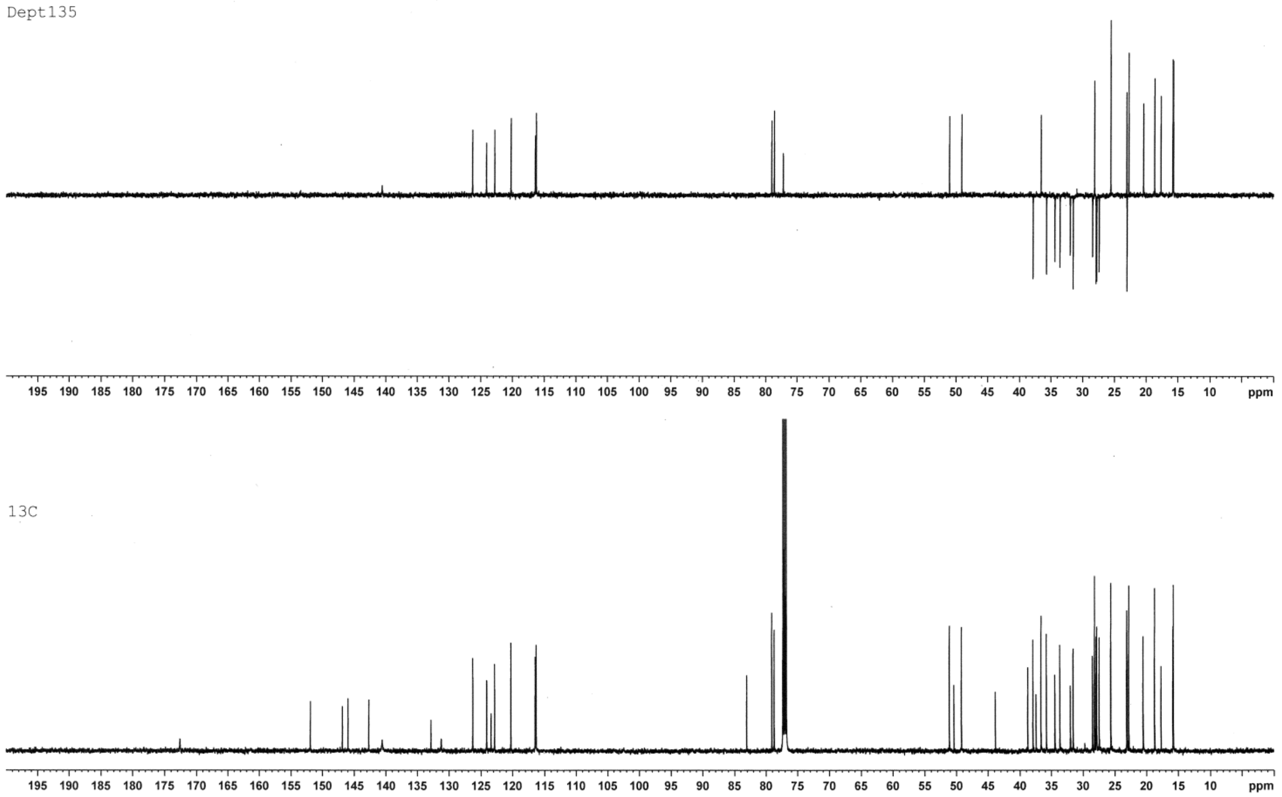

Figure S64. COSY spectrum of ganohochimin H (**8**) ($CDCl_3$, 500 MHz)

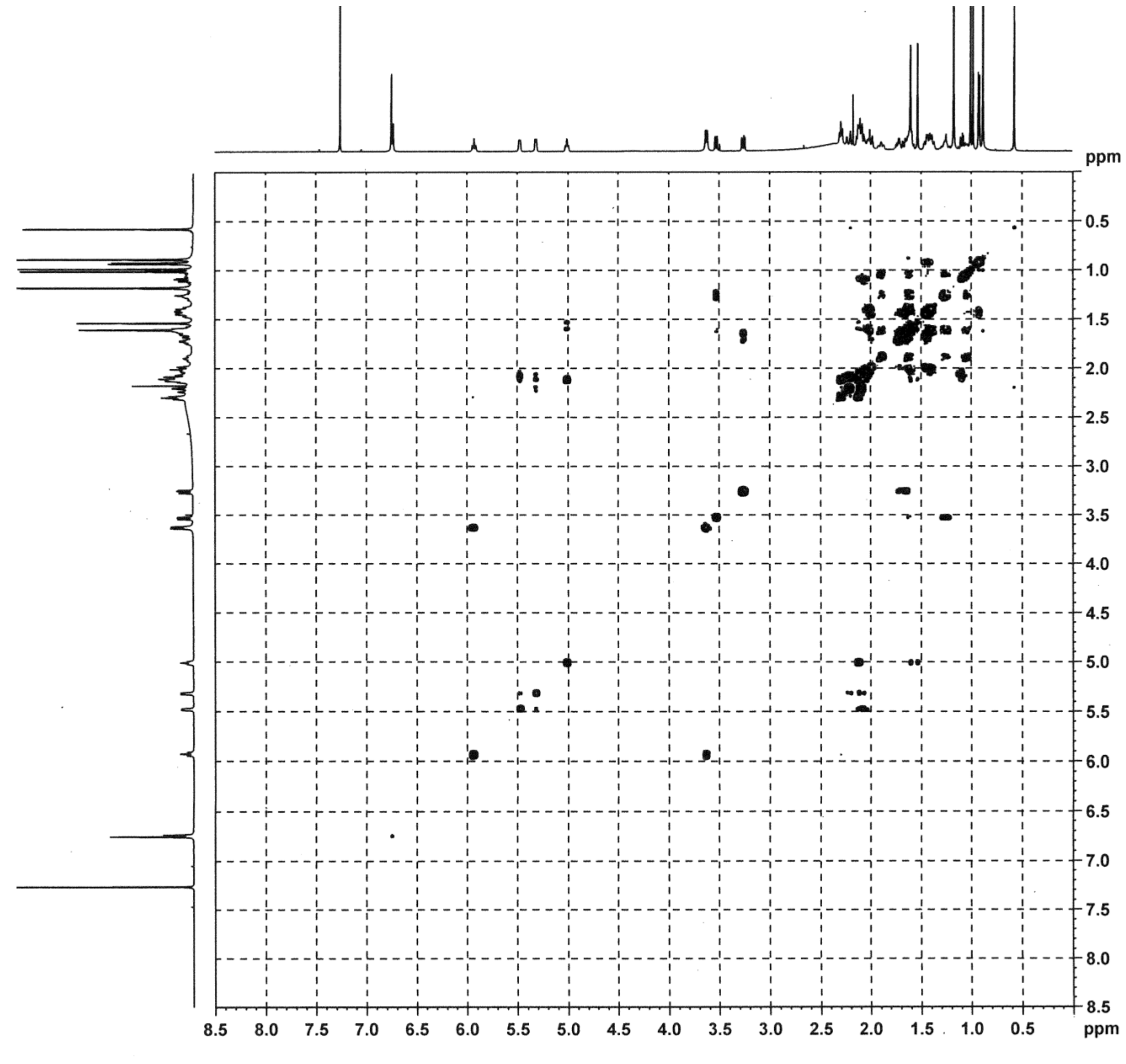

Figure S65. HSQC spectrum of ganohochimin H (**8**) ($CDCl_3$)

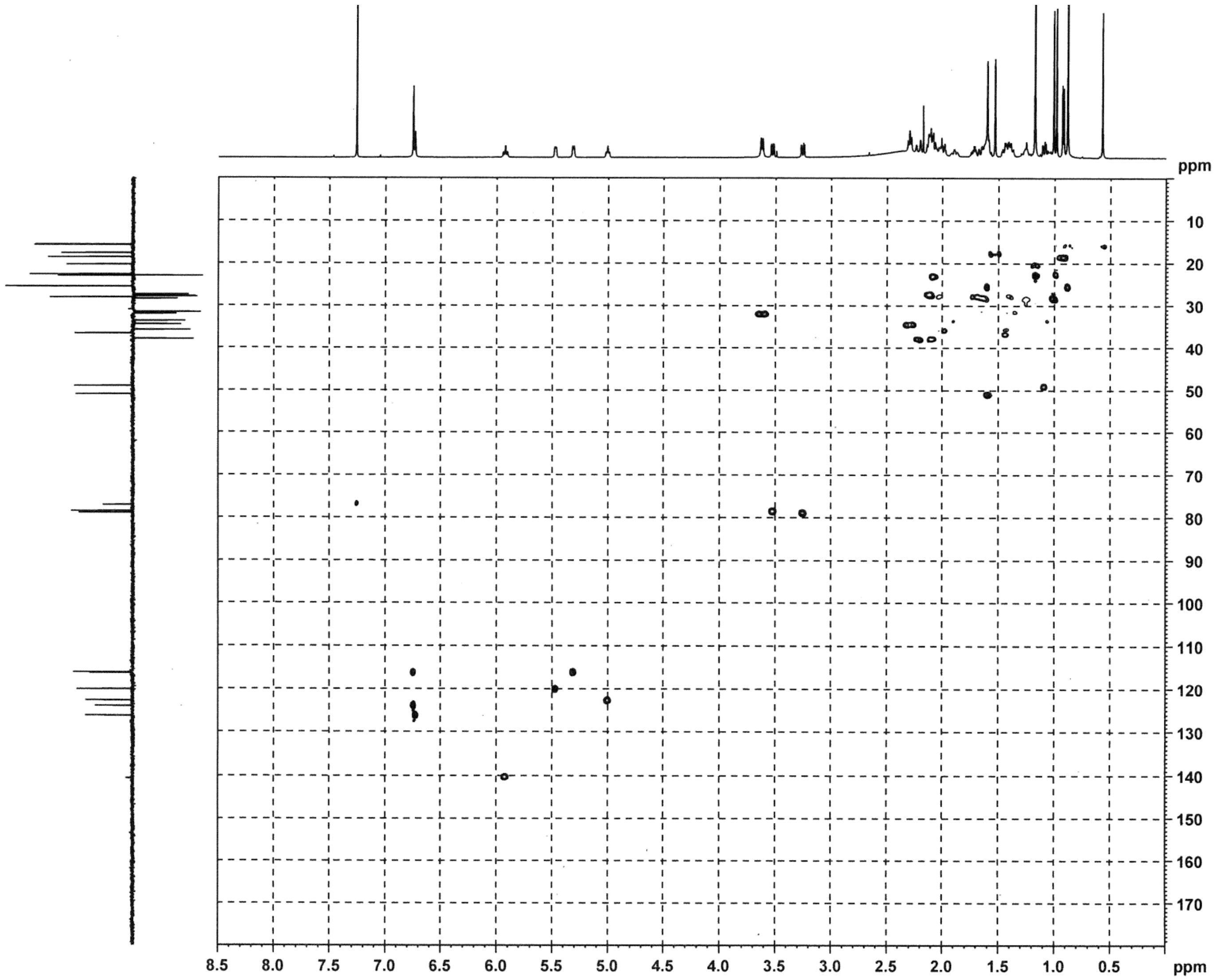

Figure S66. HMBC spectrum of ganohochimin H (**8**) ($CDCl_3$)

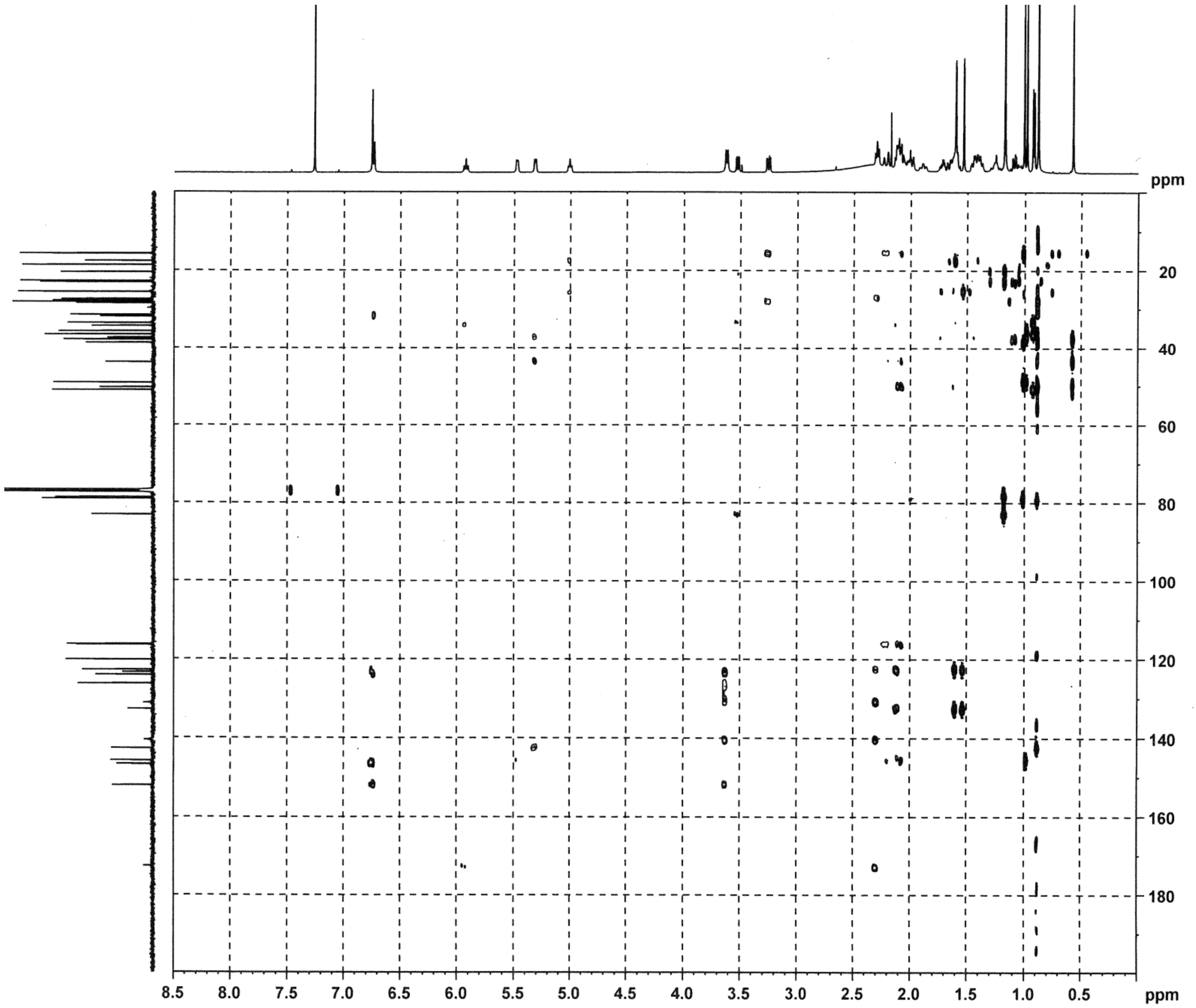

Figure S67. NOESY spectrum of ganohochimin H (**8**) ($CDCl_3$, 500 MHz)

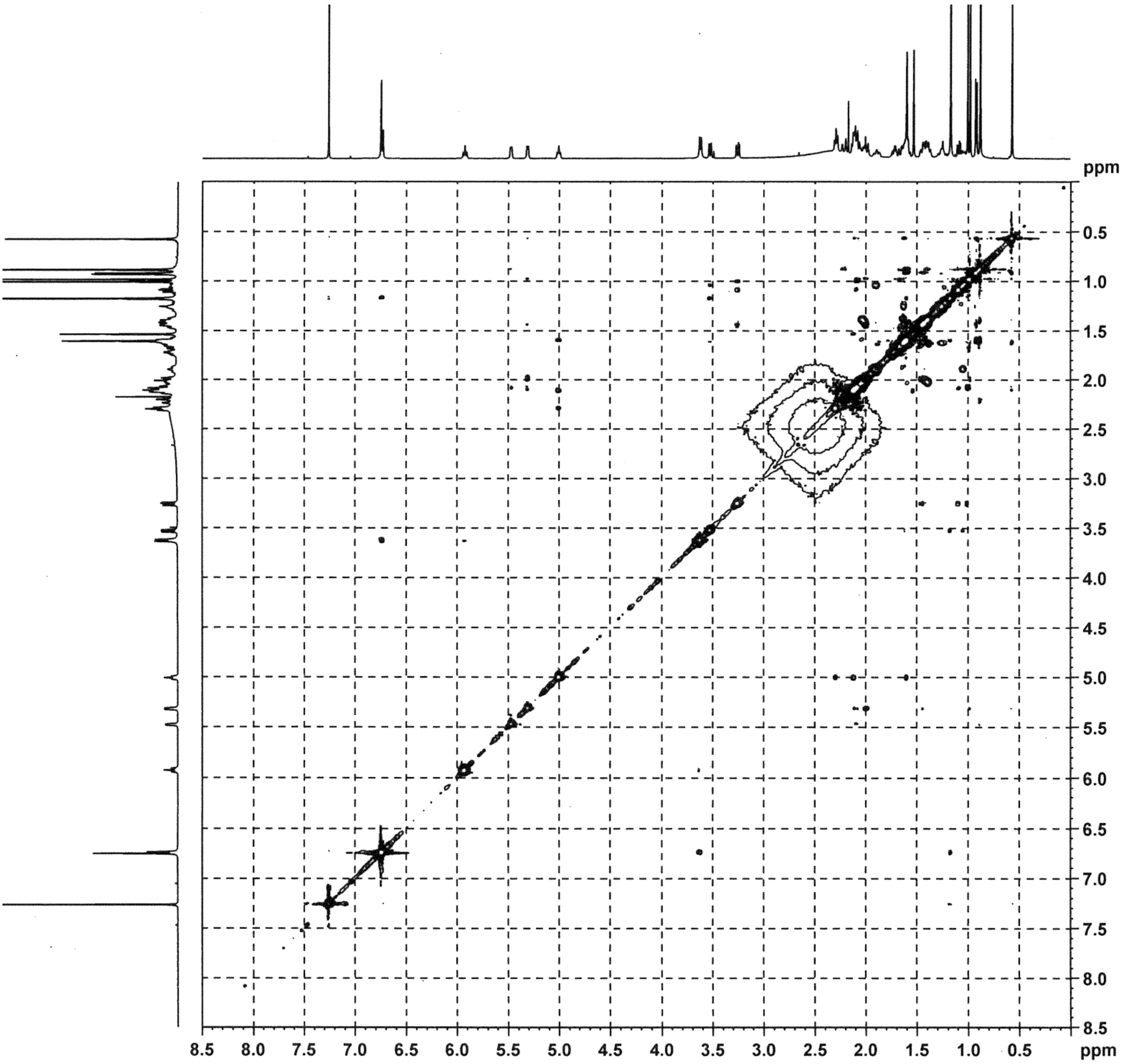

Figure S68. HRESIMS of ganohochimin H (**8**) (positive ion mode)

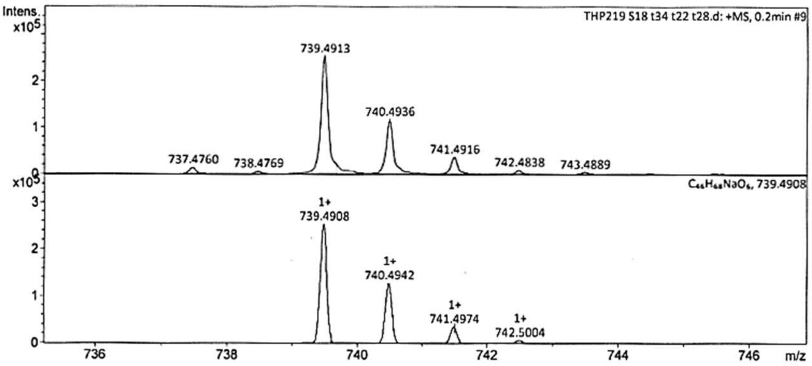

Figure S69. ^{1}H NMR spectrum of ganohochimate A (**9**) ($CDCl_3$, 500 MHz)

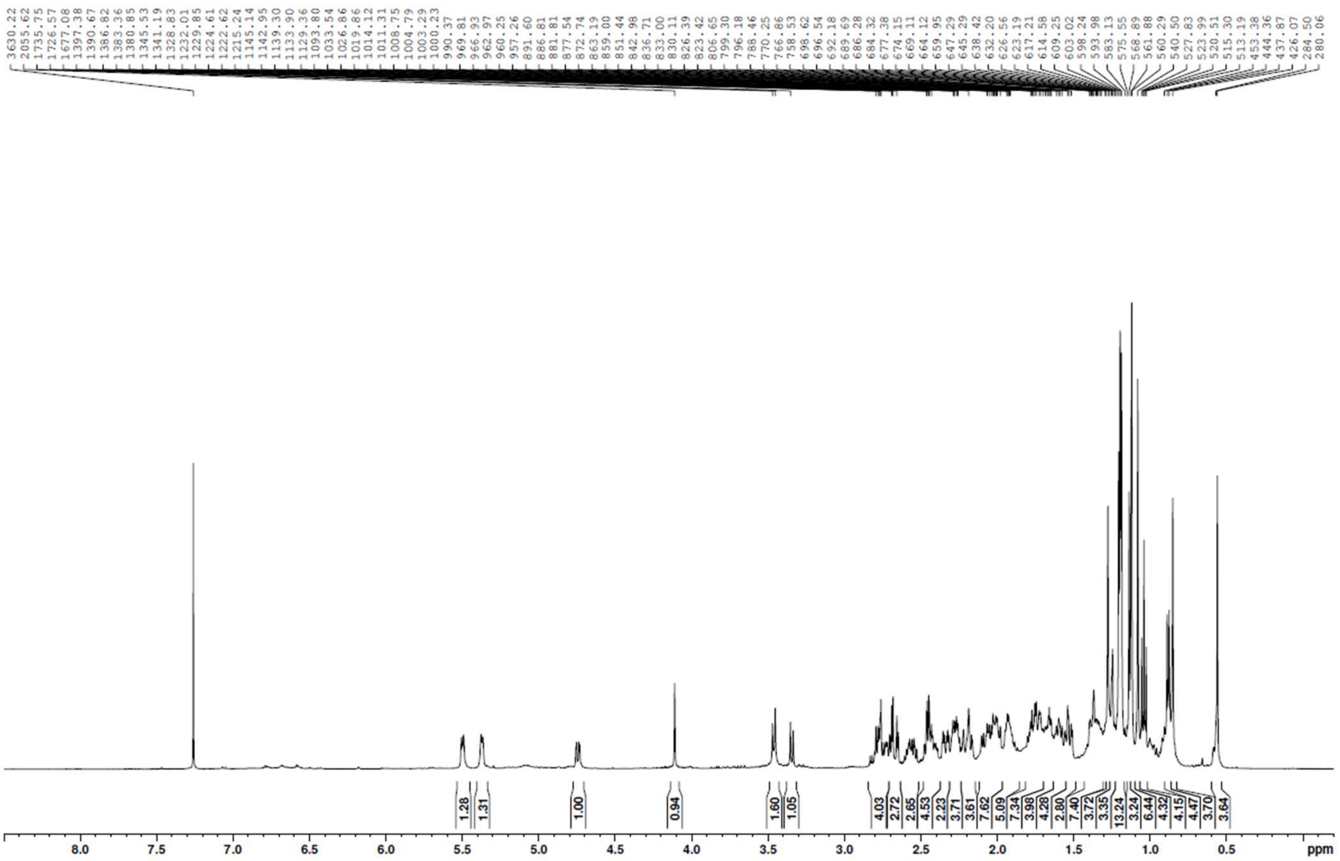

Figure S70. ^{13}C NMR spectrum of ganohochimate A (**9**) ($CDCl_3$, 125 MHz)

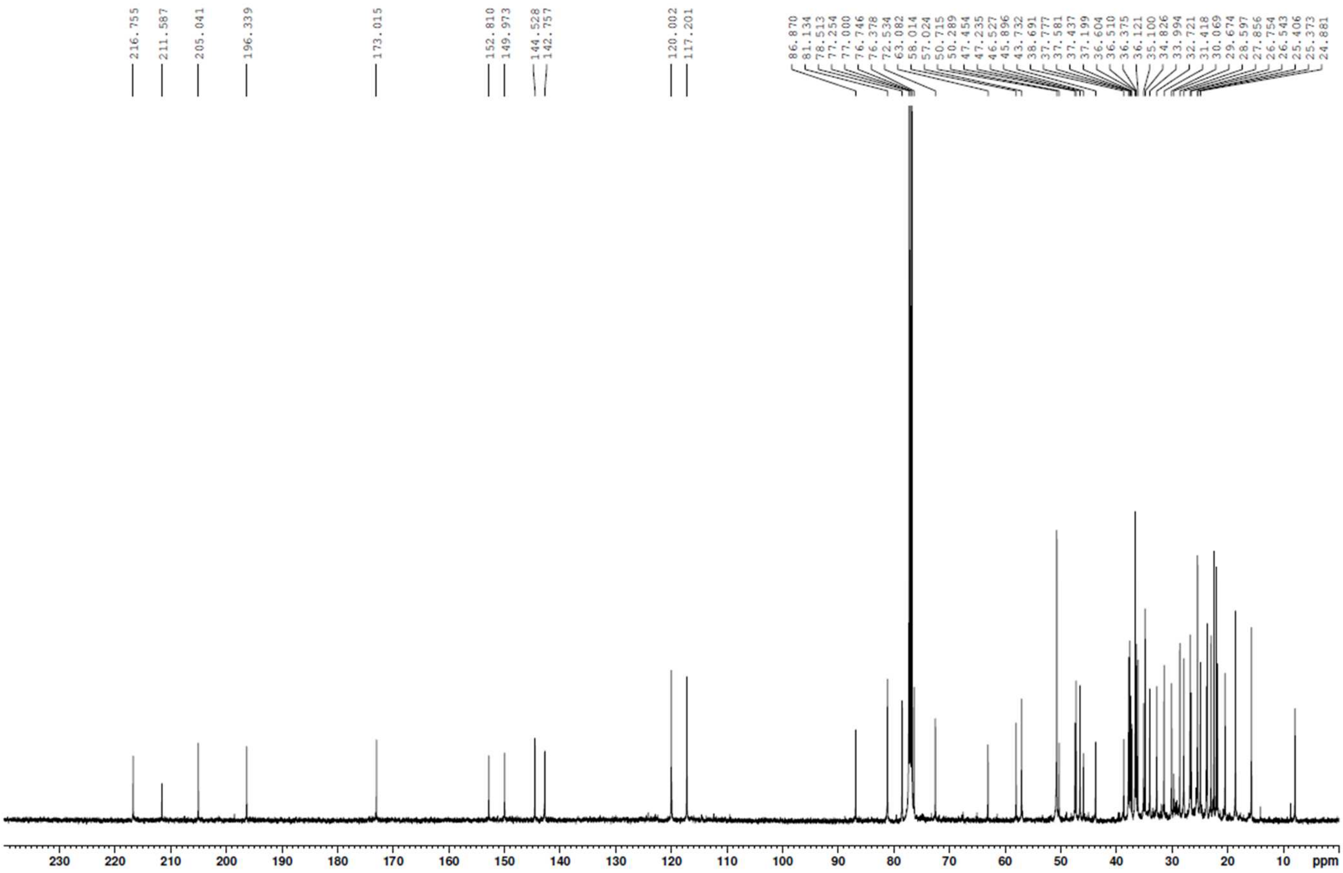

Figure S71. DEPT-135 spectrum of ganohochimate A (**9**) ($CDCl_3$, 125 MHz)

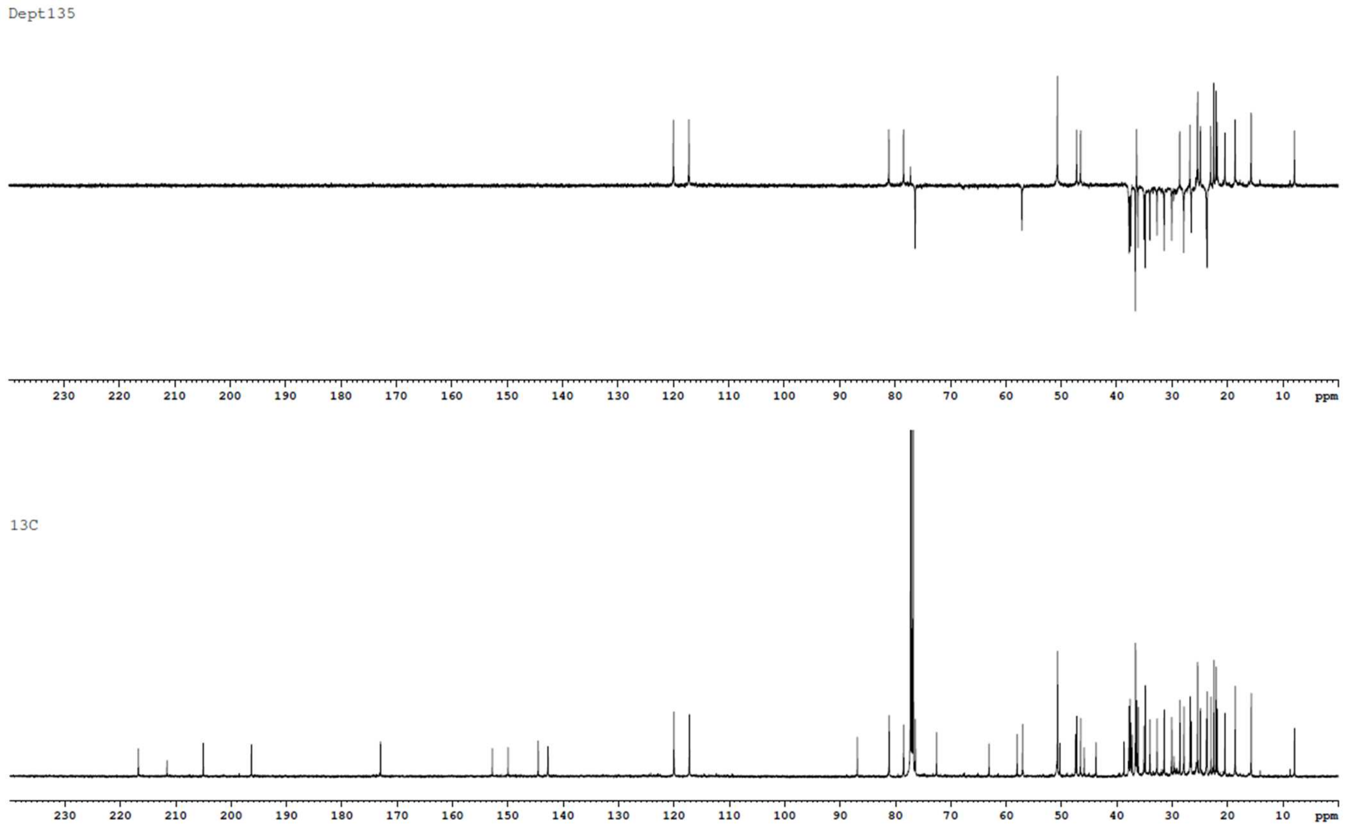

Figure S72. COSY spectrum of ganohochimate A (**9**) ($CDCl_3$, 500 MHz)

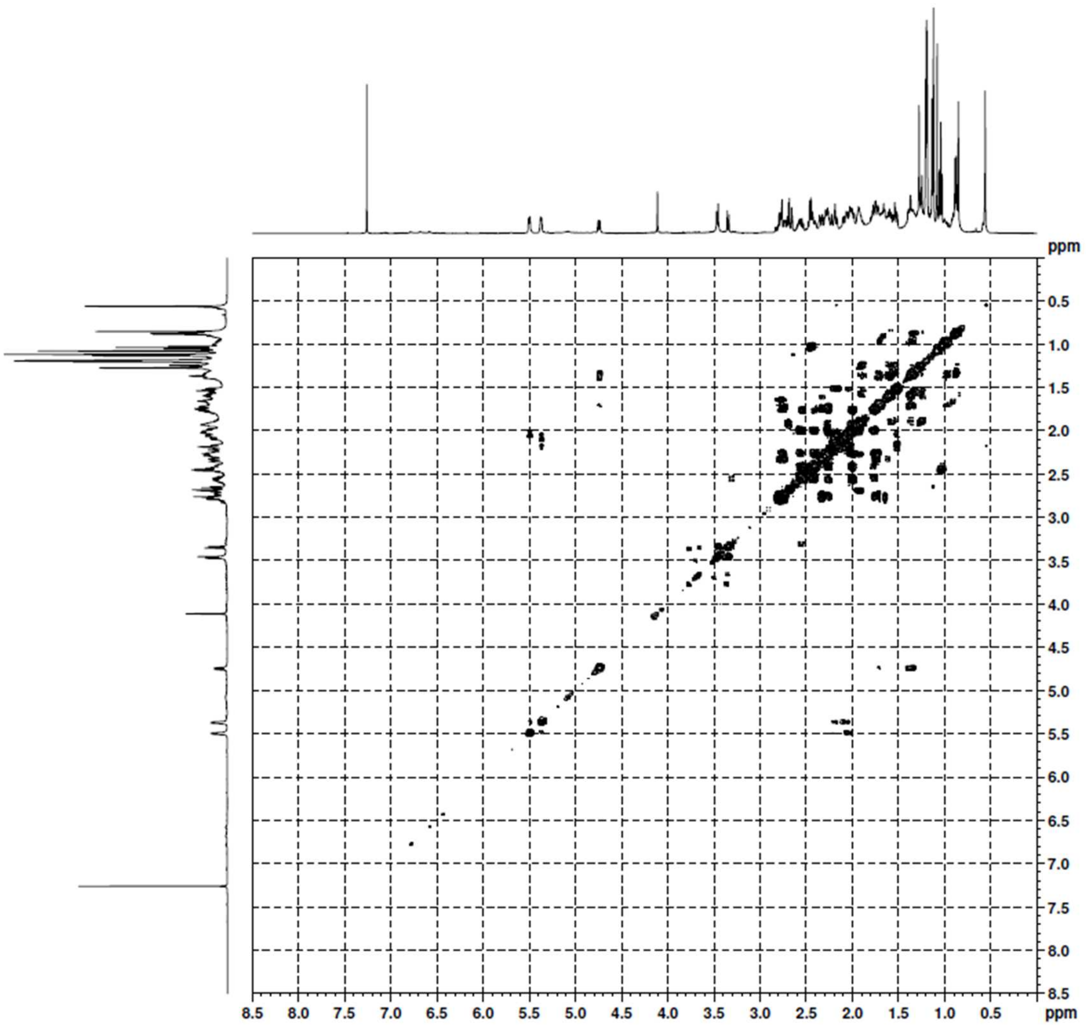

Figure S73. HSQC spectrum of ganohochimate A (**9**) ($CDCl_3$)

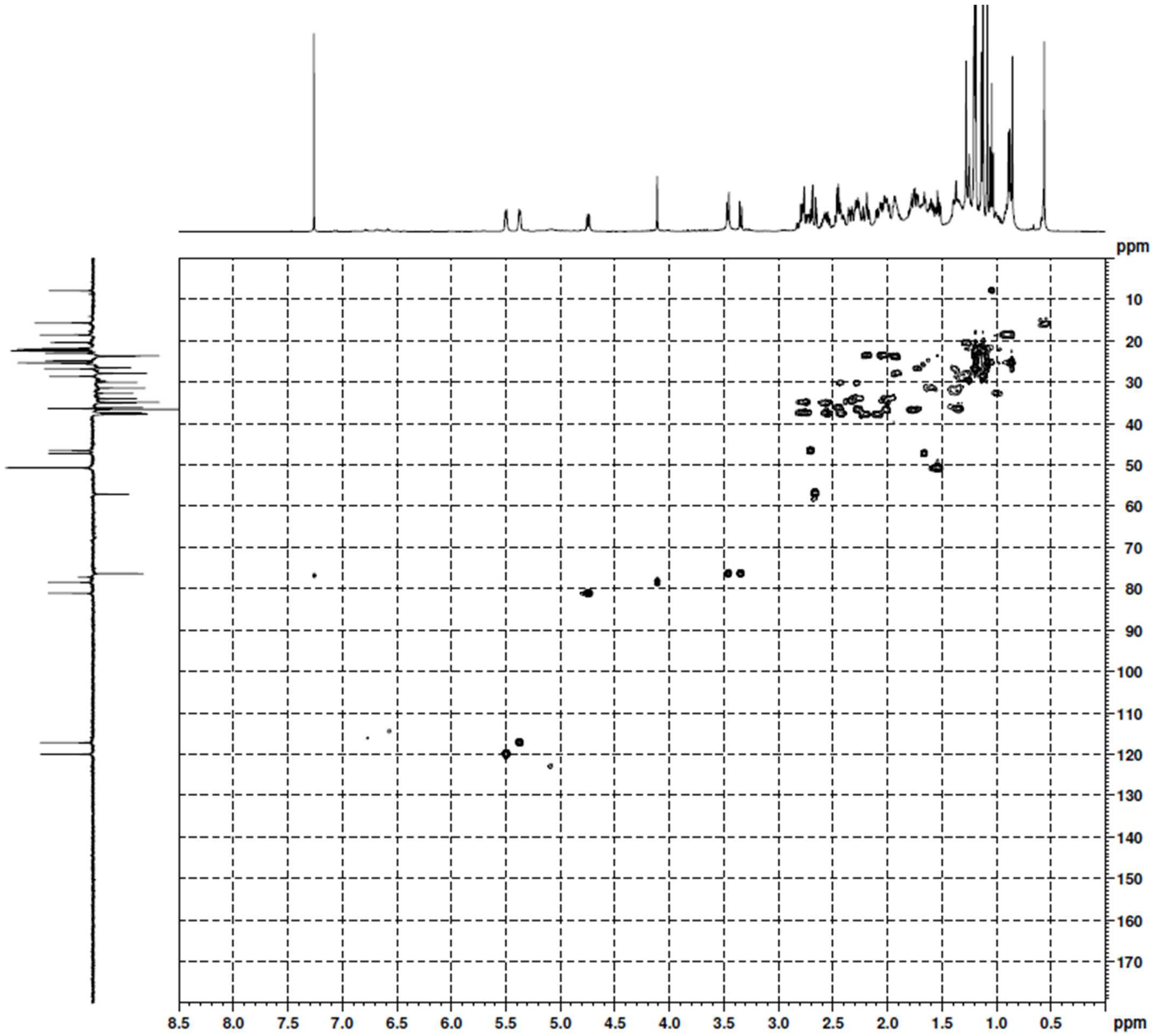

Figure S74. HMBC spectrum of ganohochimate A (**9**) ($CDCl_3$)

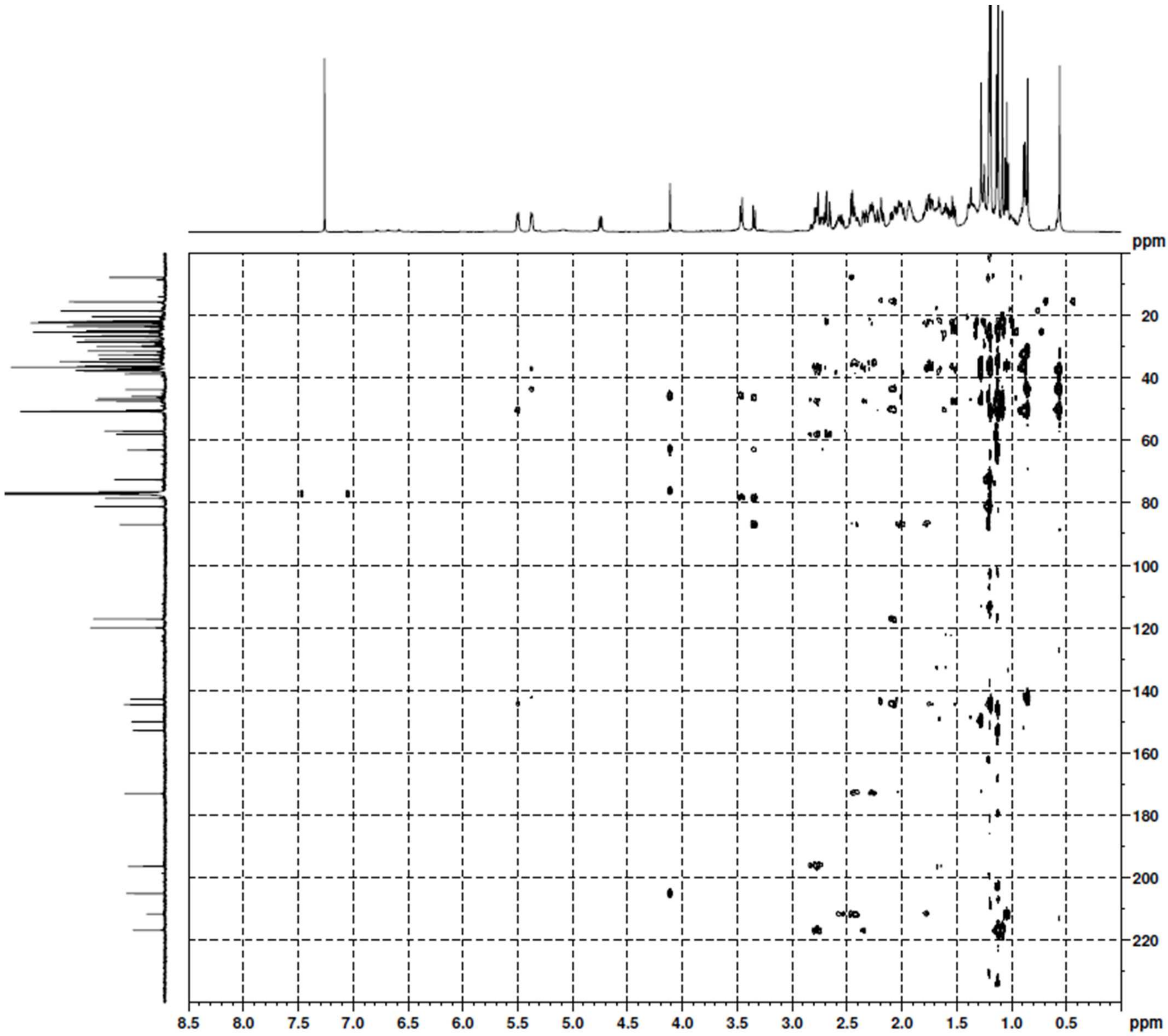

Figure S75. NOESY spectrum of ganohochimate A (**9**) ($CDCl_3$, 500 MHz)

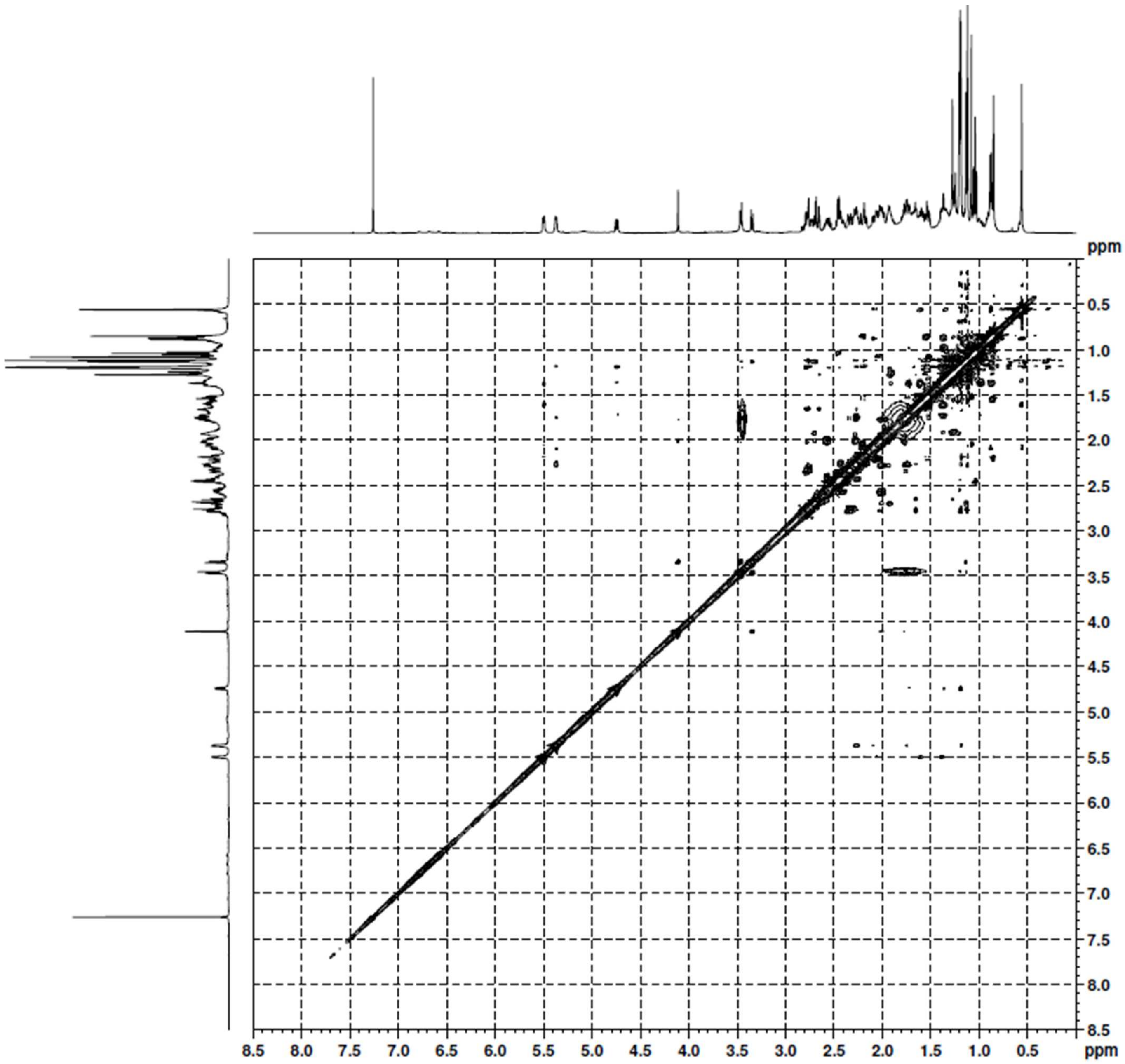

Figure S76. HRESIMS of ganohochimate A (**9**) (positive ion mode)

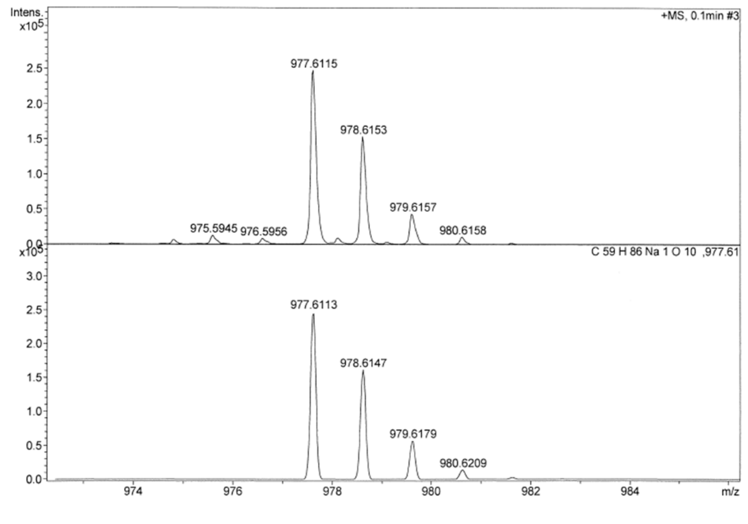

Figure S77. ^{1}H NMR spectrum of fornicin F (**10**) ($CDCl_3$, 400 MHz)

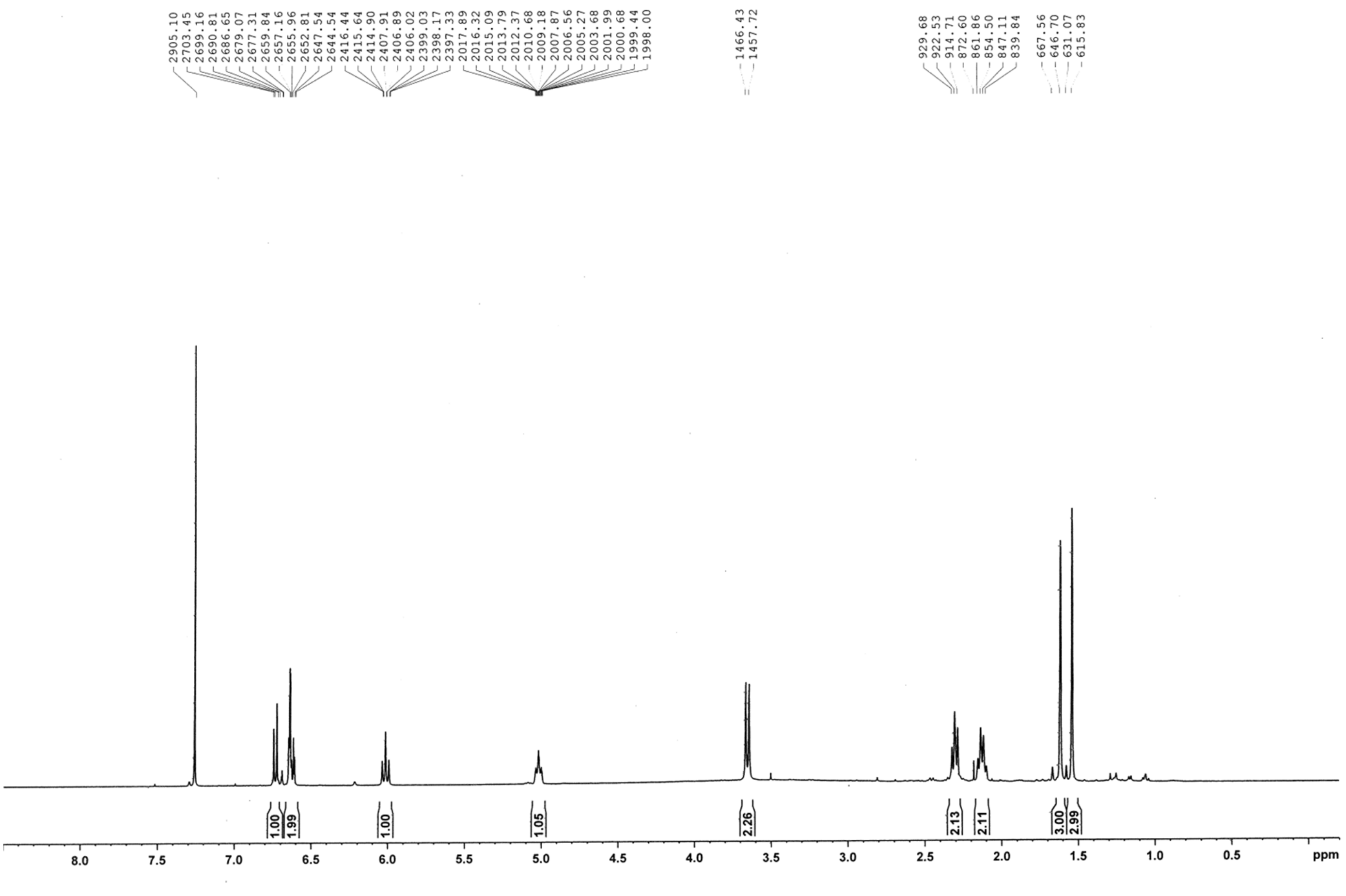

Figure S78. ^{13}C NMR spectrum of fornicin F (**10**) ($CDCl_3$, 100 MHz)

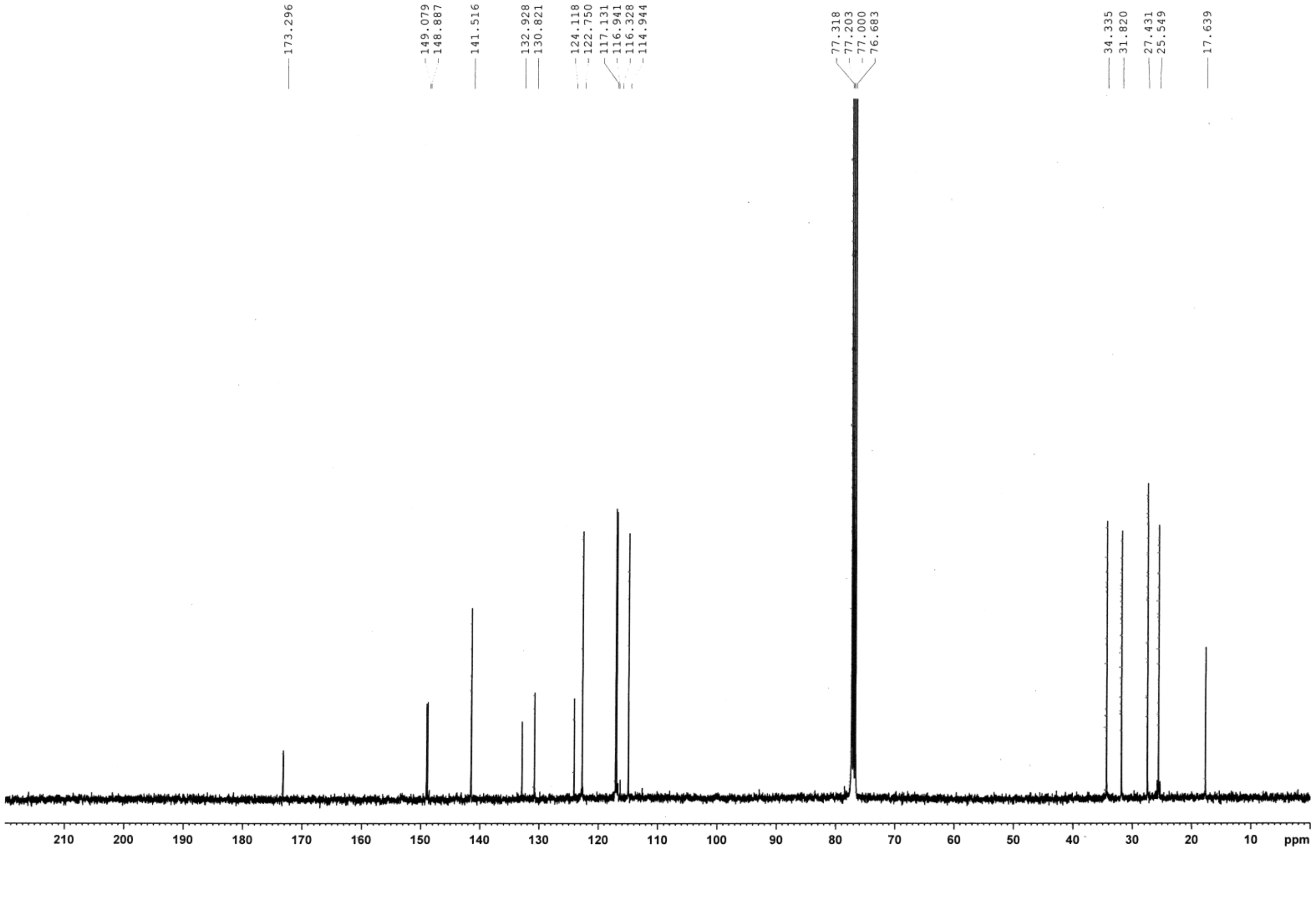

Figure S79. DEPT-135 spectrum of fornicin F (**10**) ($CDCl_3$, 100 MHz)

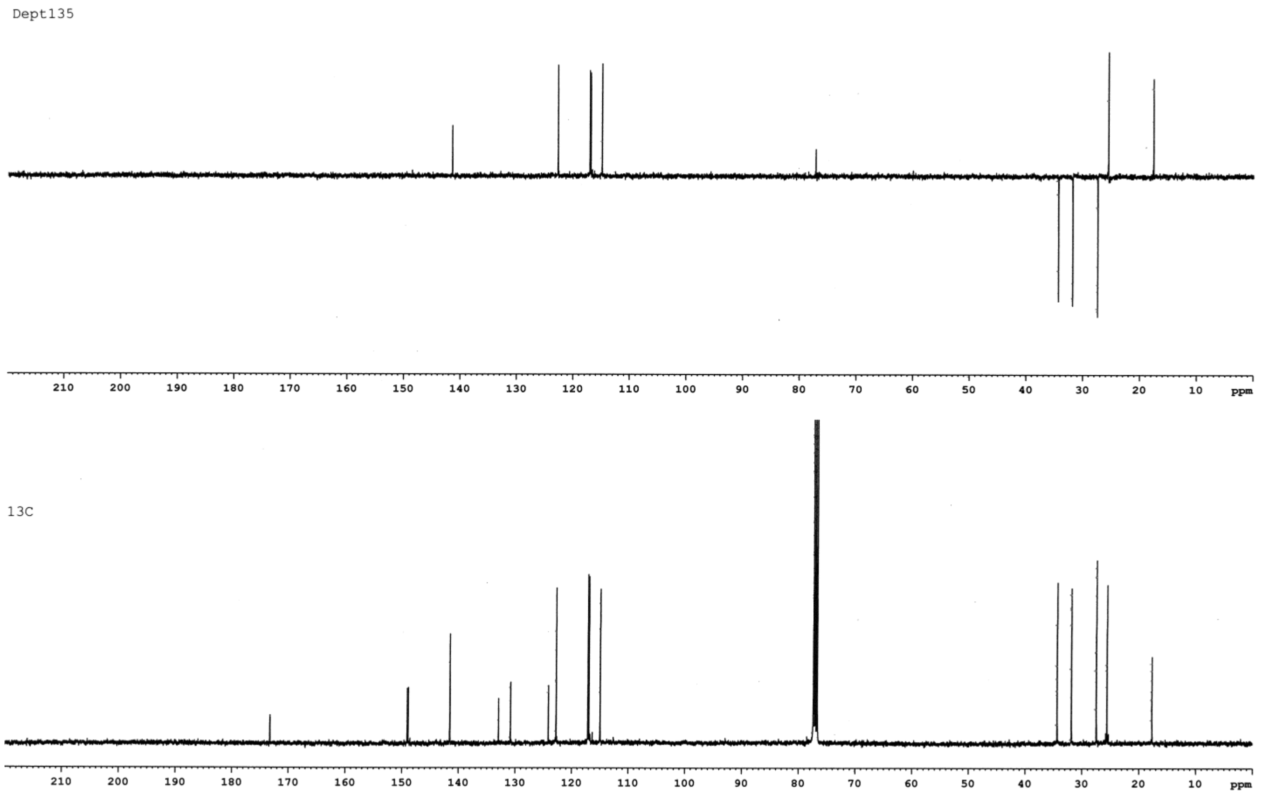

Figure S80. COSY spectrum of fornicin F (**10**) ($CDCl_3$, 400 MHz)

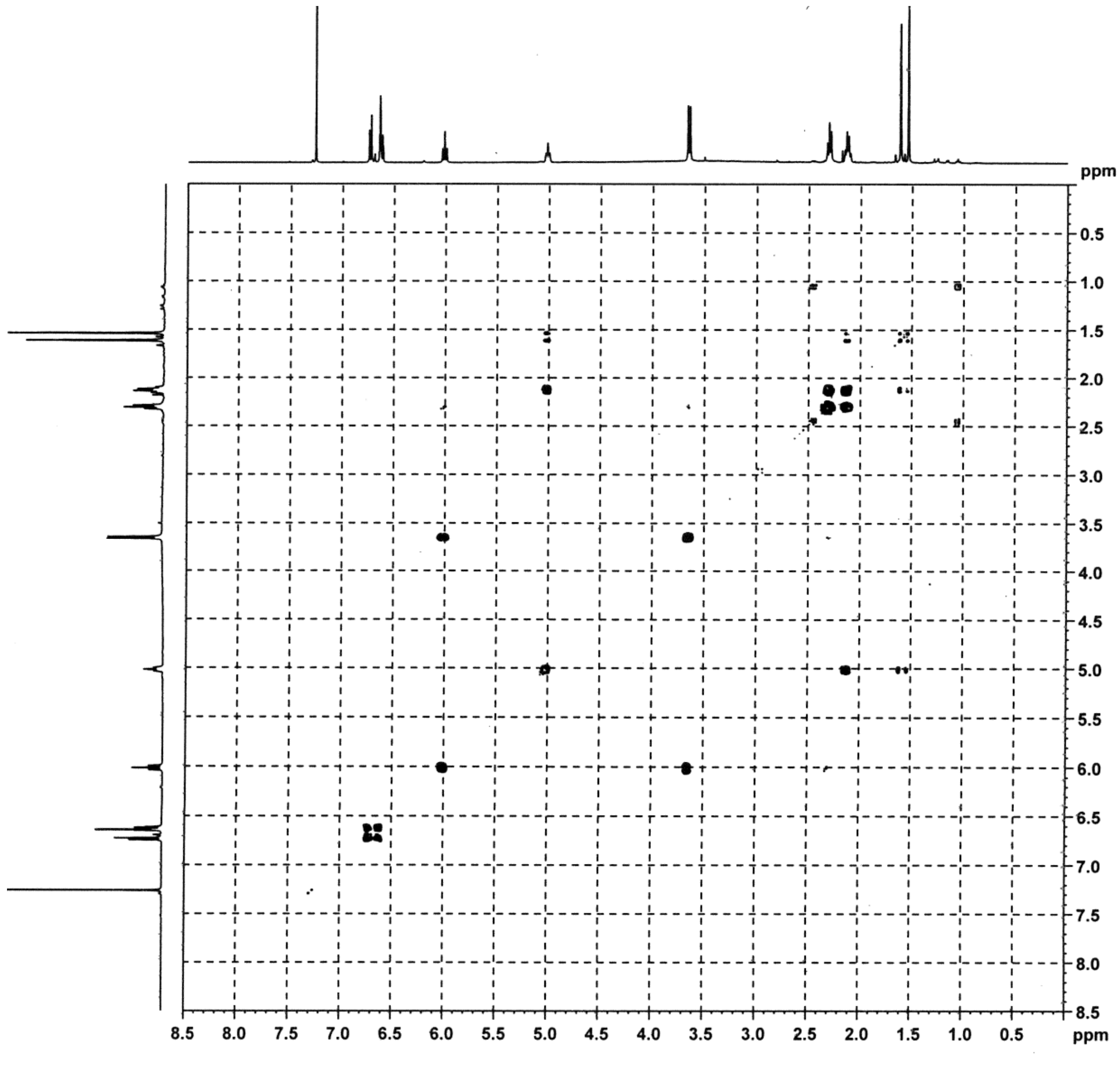

Figure S81. HMQC spectrum of fornicin F (**10**) ($CDCl_3$)

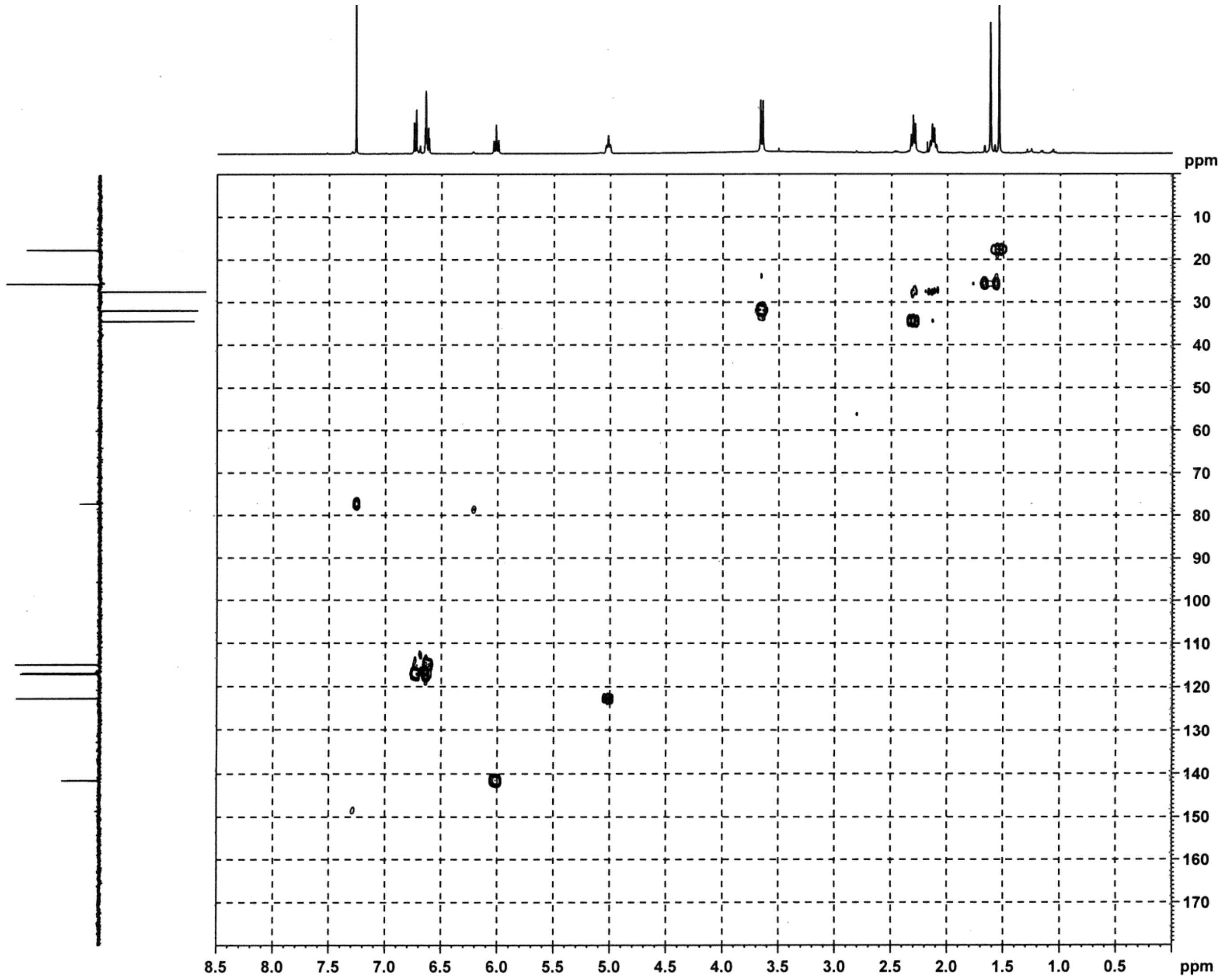

Figure S82. HMBC spectrum of fornicin F (**10**) ($CDCl_3$)

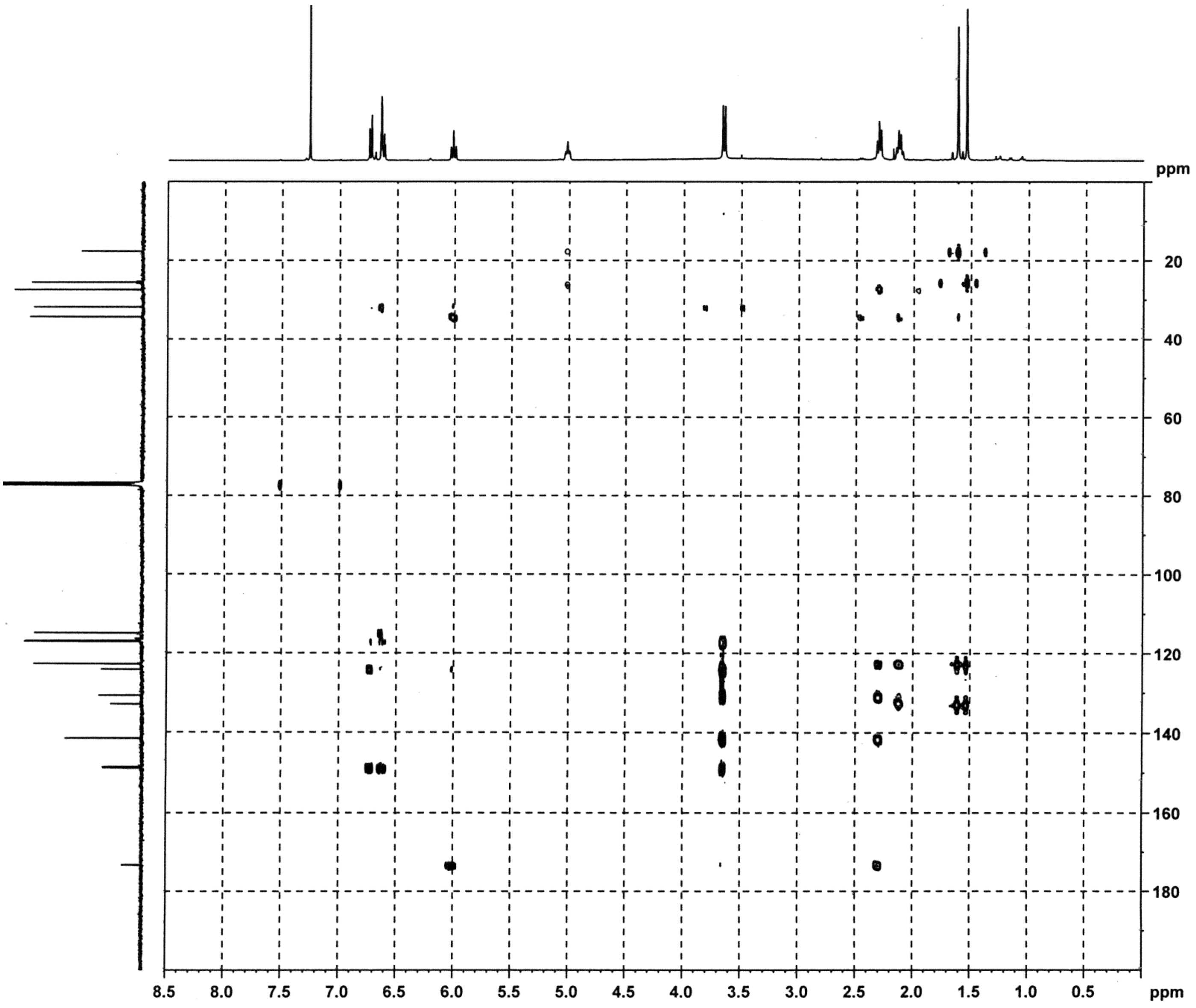

Figure S83. NOESY spectrum of fornicin F (**10**) ($CDCl_3$, 400 MHz)

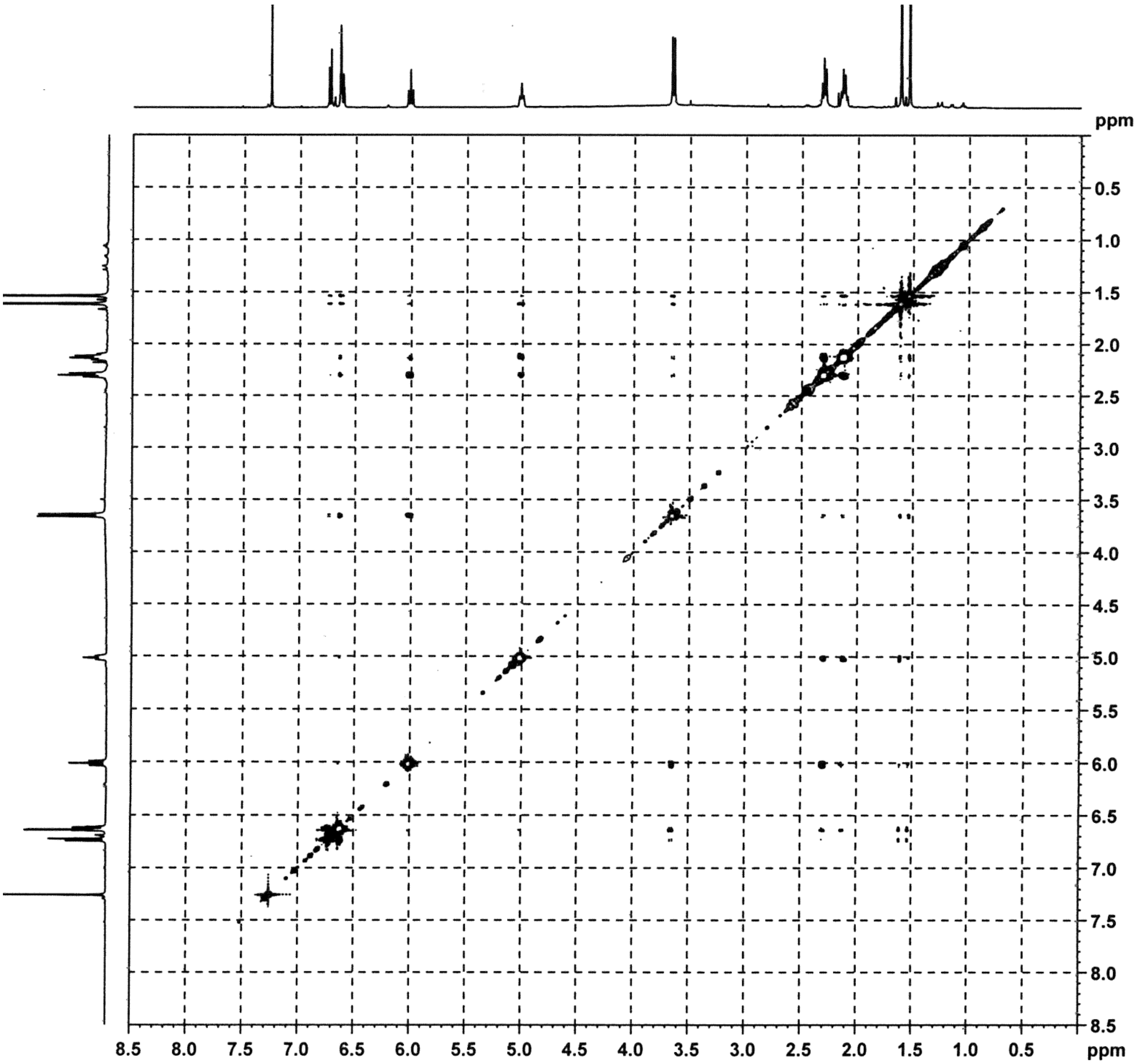

Figure S84. HRESIMS of fornicin F (**10**) (positive ion mode)

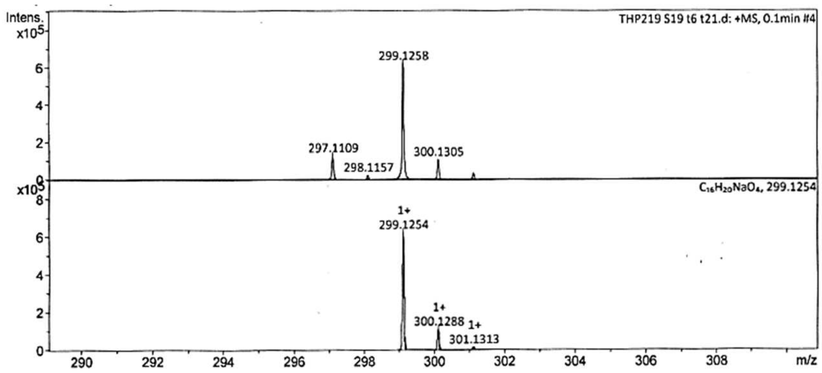

Figure S85. ^{1}H NMR spectrum of compound **37** ($CDCl_3$, 500 MHz)

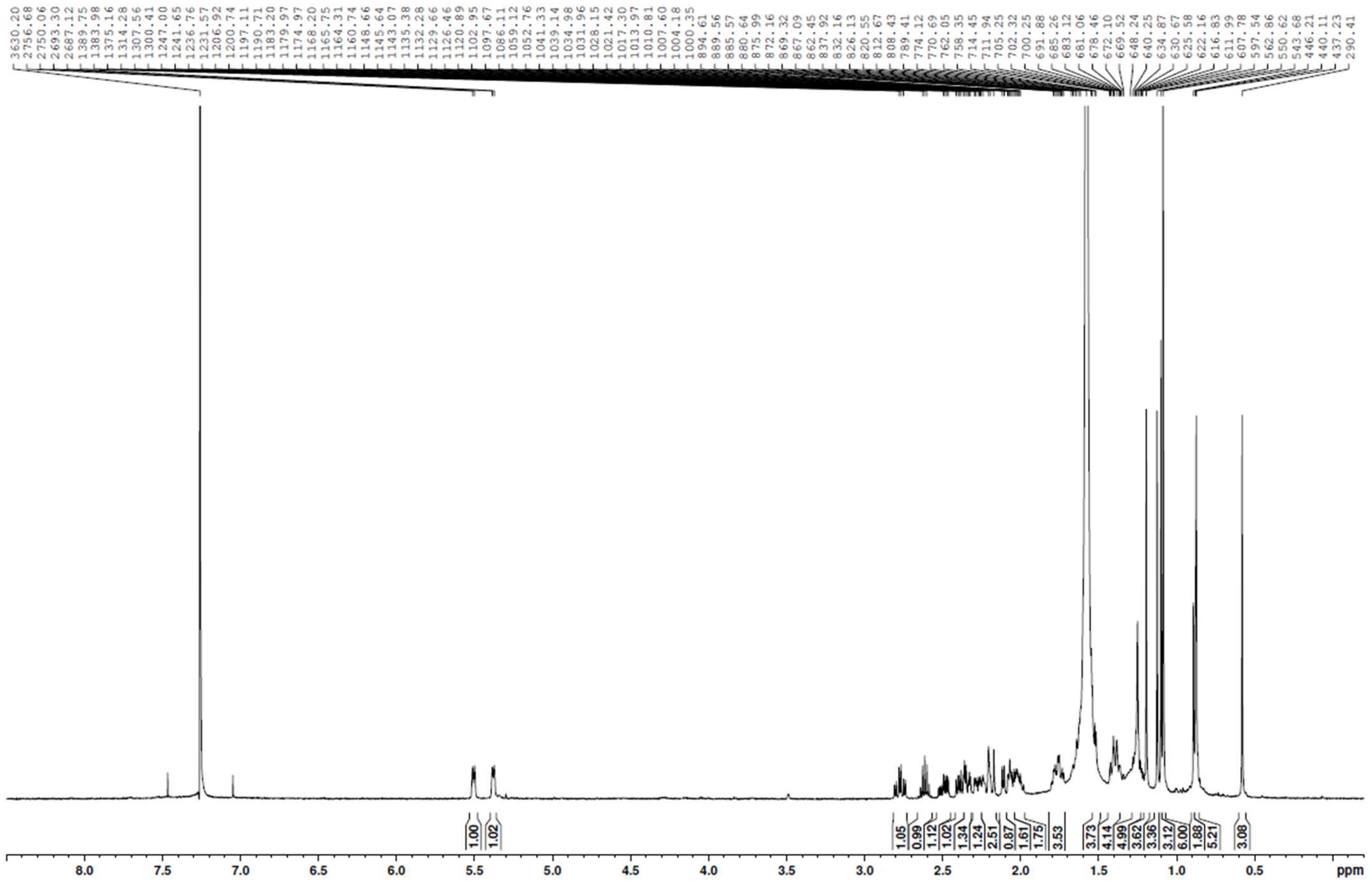

Figure S86. ^{13}C NMR spectrum of compound **37** ($CDCl_3$, 125 MHz)

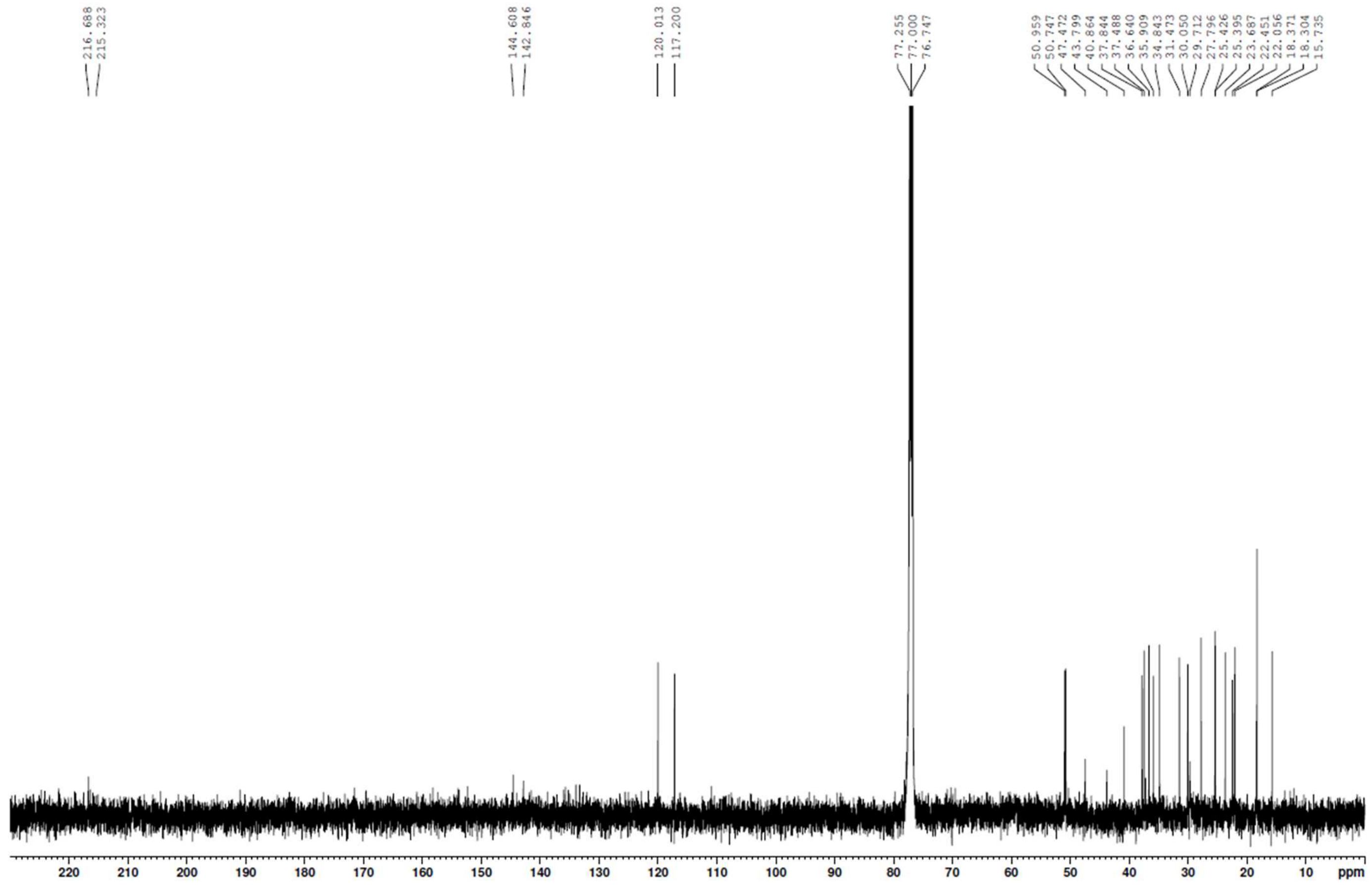

Figure S87. DEPT-135 spectrum of compound **37** ($CDCl_3$, 125 MHz)

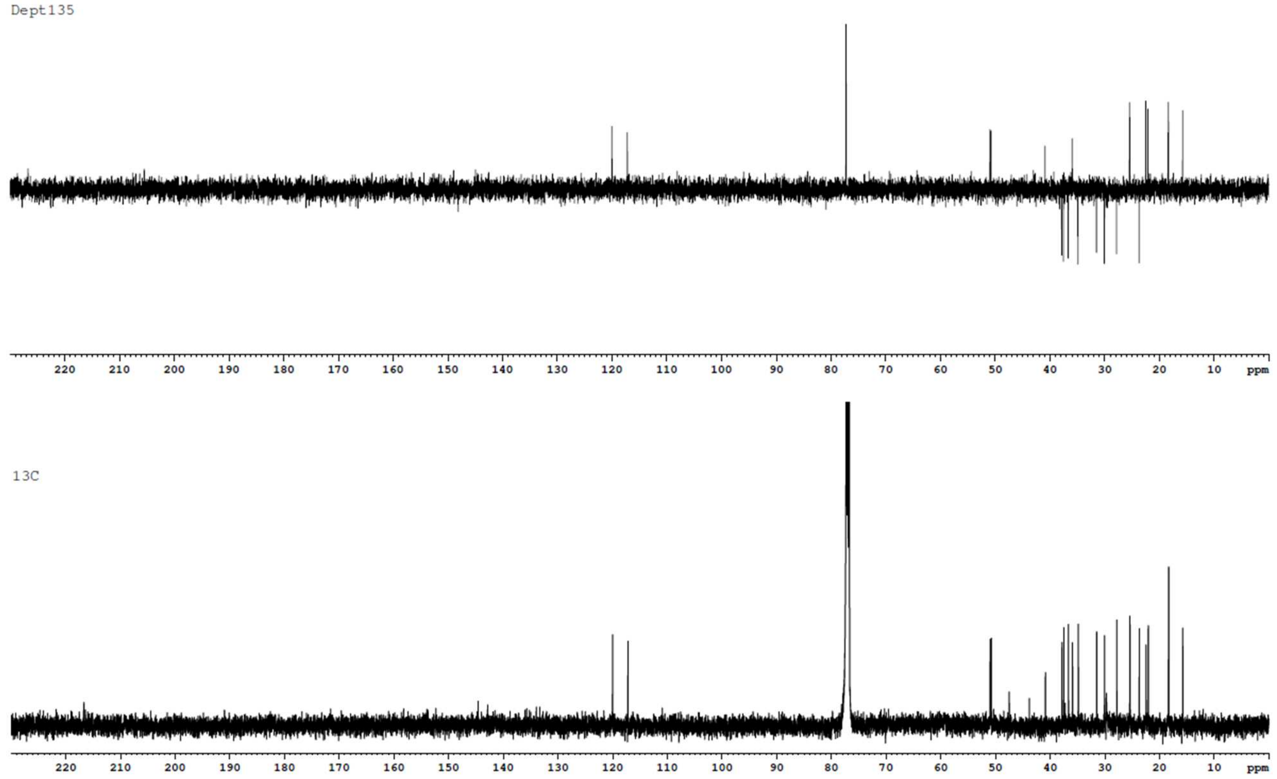

Figure S88. COSY spectrum of compound **37** ($CDCl_3$, 500 MHz)

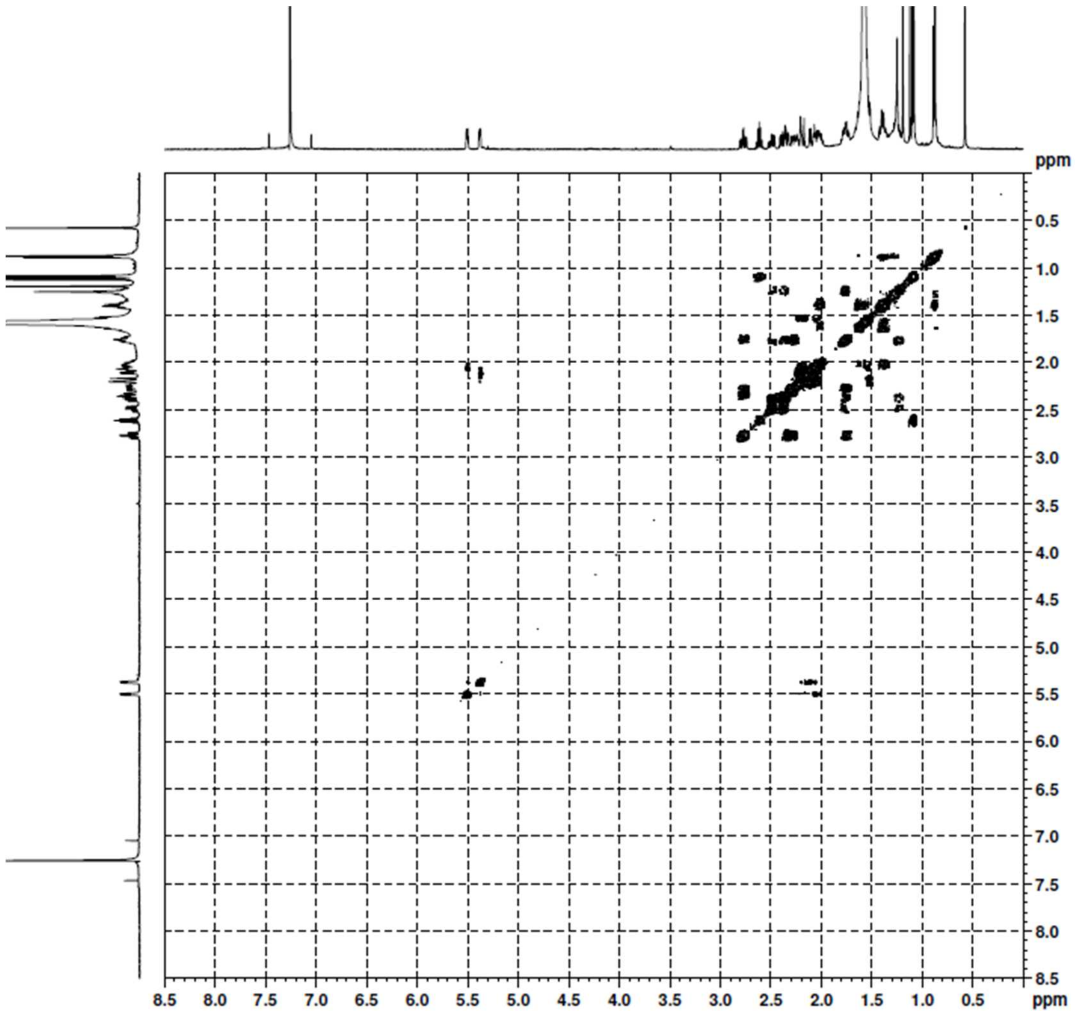

Figure S89. HSQC spectrum of compound **37** ($CDCl_3$)

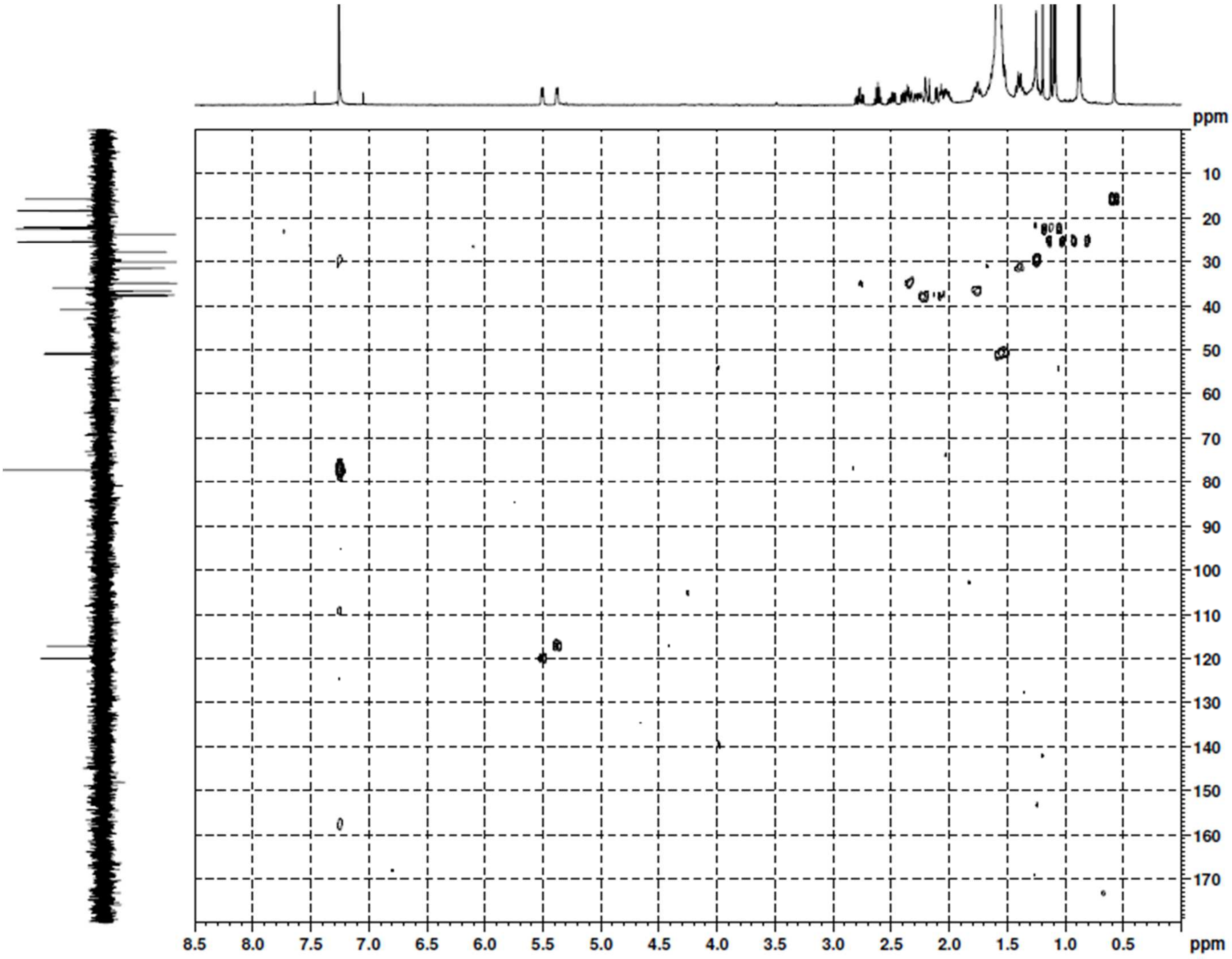

Figure S90. HMBC spectrum of compound **37** ($CDCl_3$)

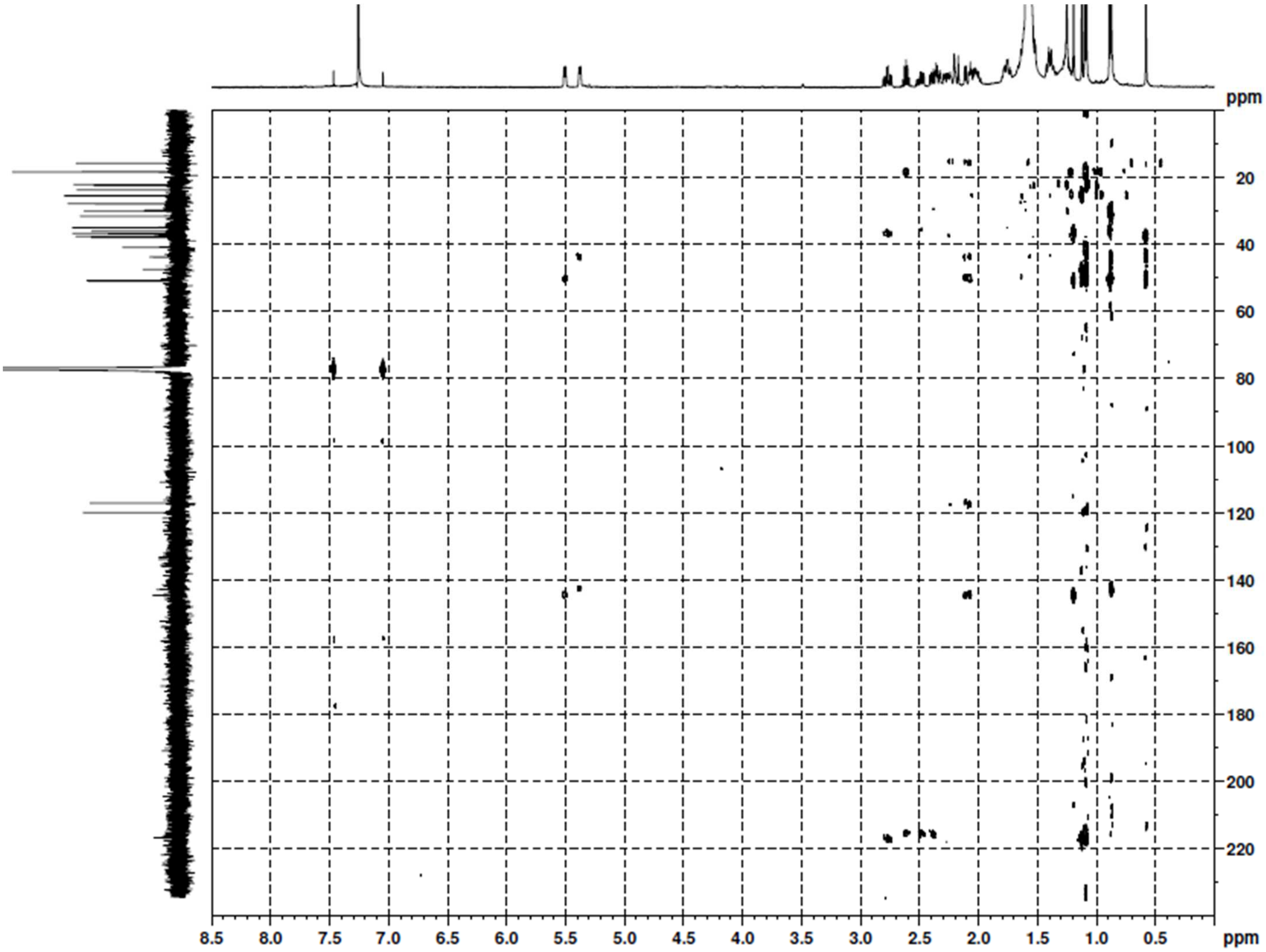

Figure S91. NOESY spectrum of compound **37** ($CDCl_3$, 500 MHz)

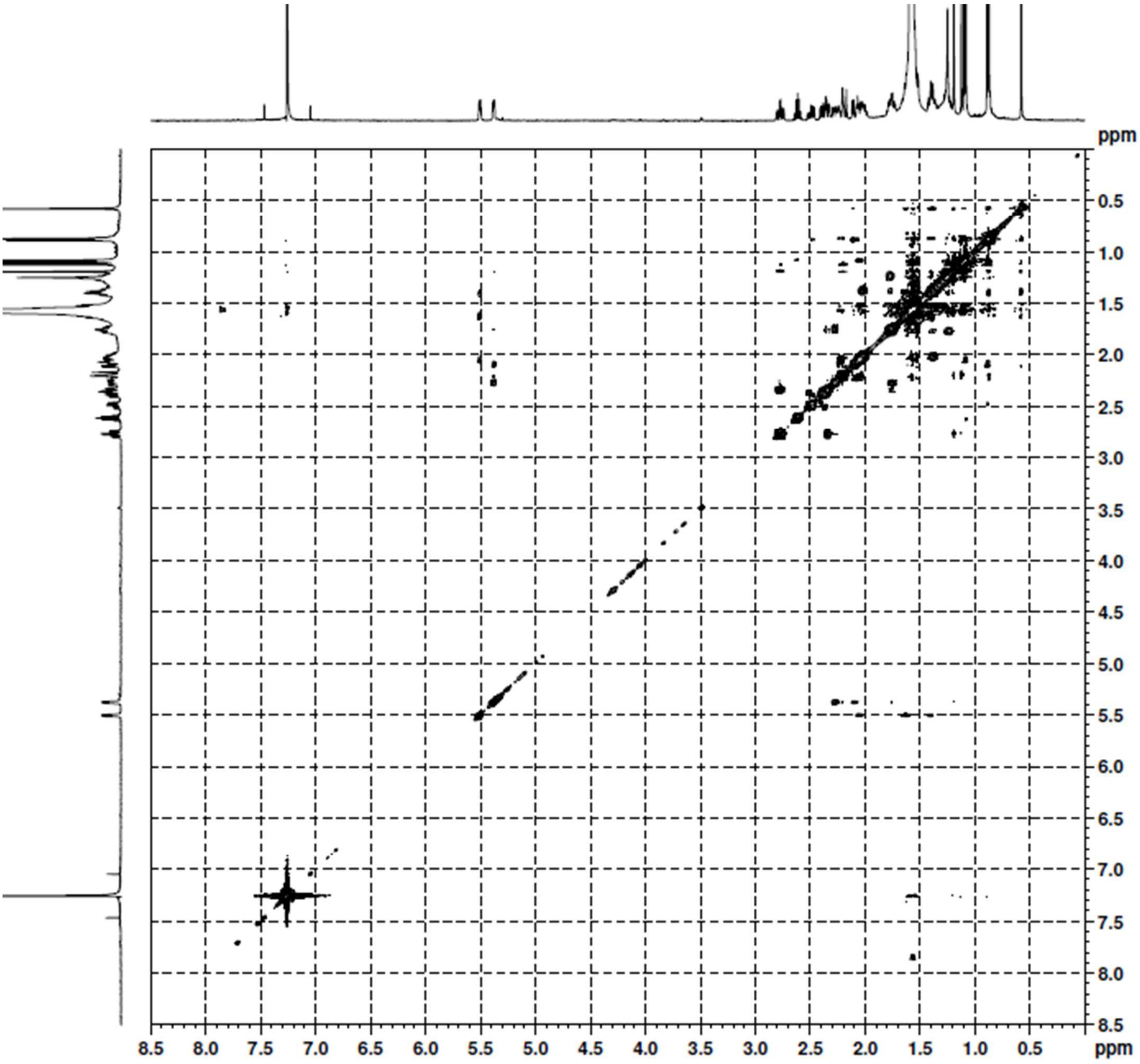

Figure S92. HRESIMS of compound **37** (positive ion mode)

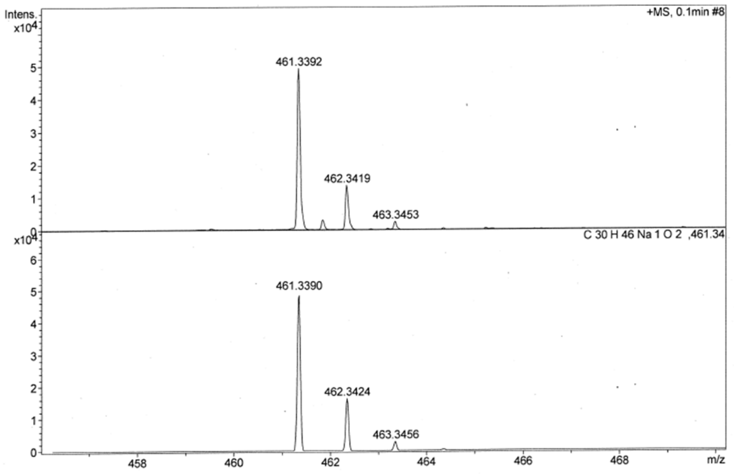

Supplement: Supplementary file 1 [file ao5c06826_si_001.pdf]
